# Diameter in ultra‐small scale‐free random graphs

**DOI:** 10.1002/rsa.20798

**Published:** 2018-11-12

**Authors:** Francesco Caravenna, Alessandro Garavaglia, Remco van der Hofstad

**Affiliations:** ^1^ Dipartimento di Matematica e Applicazioni Università degli Studi di Milano‐Bicocca Milano Italy; ^2^ Department of Mathematics and Computer Science Eindhoven University of Technology Eindhoven The Netherlands

**Keywords:** configuration model, diameter, preferential attachment model, random graphs, scale free, ultra‐small

## Abstract

It is well known that many random graphs with infinite variance degrees are ultra‐small. More precisely, for configuration models and preferential attachment models where the proportion of vertices of degree at least k is approximately k ^−(τ − 1)^ with τ ∈ (2,3), typical distances between pairs of vertices in a graph of size n are asymptotic to $\frac{2\log\log n}{\left|\log(\tau -2)\right|}$ and $\frac{4\log\log n}{\left|\log(\tau -2)\right|}$, respectively. In this paper, we investigate the behavior of the diameter in such models. We show that the diameter is of order loglogn precisely when the minimal forward degree d_fwd_ of vertices is at least 2. We identify the exact constant, which equals that of the typical distances plus $2/\log d_{\text{fwd}}$. Interestingly, the proof for both models follows identical steps, even though the models are quite different in nature.

## 1 INTRODUCTION AND RESULTS

In this paper, we study the diameter of two different random graph models: the configuration model and the preferential attachment model, when these two models have a power‐law degree distribution with exponent *τ* ∈ (2,3), so that the degrees have finite mean but infinite variance. In this first section, we give a brief introduction to the models, stating the main technical conditions required as well as the two main results proved in the paper.

Throughout the paper, we write “with high probability” to mean “with probability 1 − *o*(1) as *n*→*∞*, or as *t*→*∞*,” where *n* and *t* denote the number of vertices in the configuration model and in the preferential attachment model, respectively.

### 1.1 Configuration model and main result

The configuration model CM_*n*_ is a random graph with vertex set [*n*]: = {1,2,…,*n*} and with prescribed degrees. Let ***d***=(*d*_1_,*d*_2_,…,*d*_*n*_) be a given *degree sequence*, that is, a sequence of *n* positive integers with total degree

(1.1) $$\ell_{n}=\sum_{i\in [n]}d_{i},$$

assumed to be even. The configuration model (CM) on *n* vertices with degree sequence ***d*** is constructed as follows: Start with *n* vertices and *d*_*i*_ half‐edges adjacent to vertex *i* ∈ [*n*]. Randomly choose pairs of half‐edges and match the chosen pairs together to form edges. Although self‐loops may occur, these become rare as *n*→*∞* (see eg, [2, Theorem 2.16], 19). We denote the resulting multi‐graph on [*n*] by CM_*n*_, with corresponding edge set _*n*_. We often omit the dependence on the degree sequence ***d***, and write CM_*n*_ for CM_*n*_(***d***).

#### 1.1.1 Regularity of vertex degrees

Let us now describe our regularity assumptions. For each *n* ∈ ℕ we have a degree sequence $d^{\left(n\right)}=(d_{1}^{\left(n\right)},\text{⋯},d_{n}^{\left(n\right)})$. To lighten notation, we omit the superscript (*n*) and write ***d*** instead of ***d*** ^(*n*)^ or (***d*** ^(*n*)^)_*n* ∈ ℕ_ and *d*_*i*_ instead of $d_{i}^{\left(n\right)}$. Let (*p*_*k*_)_*k* ∈ ℕ_ be a probability mass function on N. We introduce the empirical degree distribution of the graph as

(1.2) $$p_{k}^{\left(n\right)}=\frac{1}{n}\sum_{i\in [n]}{⊮}_{\left\{d_{i}=k\right\}}.$$

We can define now the *degree regularity conditions*:

Condition 1.1 (Degree regularity conditions) Let CM_*n*_ *be a configuration model, then we say that* ***d*** *satisfies the degrees regularity conditions* (*a*), (*b*), *with respect to* (*p*_*k*_)_*k* ∈ ℕ_ if:

(a) for every *k* ∈ ℕ, *as n*→*∞*
  (1.3) $$p_{k}^{\left(n\right)}\to p_{k}.$$
(b) $\sum_{k}kp_{k}<\infty ,$ *and as n*→*∞*
  (1.4) $$\sum_{k\in N}kp_{k}^{\left(n\right)}\to \sum_{k\in N}kp_{k}.$$

As notation, we write that ***d*** satisfies the d.r.c. (*a*), (*b*).

Let *F*_***d***,*n*_ be the distribution function of ${\left(p_{k}^{\left(n\right)}\right)}_{k\in N}$, that is, for *k* ∈ ℕ,

(1.5) $$F_{d,n}(k)=\frac{1}{n}\sum_{i\in [n]}{⊮}_{\left\{d_{i}\leq k\right\}}.$$

We suppose that ***d*** satisfies the d.r.c. (*a*) and (*b*) with respect to some probability mass function (*p*_*k*_)_*k* ∈ ℕ_, corresponding to a distribution function *F*.

Condition 1.2 (Polynomial distribution condition) We say that ***d*** satisfies the polynomial distribution condition with exponent *τ* ∈ (2,3) if for all *δ* > 0 there exist $\alpha =\alpha (\delta )>\frac{1}{2},$ *c*_1_(*δ*) > 0 and *c*_2_(*δ*) > 0 such that, for every *n* ∈ ℕ, the lower bound

(1.6) $$1-F_{d,n}(x)\geq c_{1}x^{-(\tau -1+\delta )}$$

holds for all *x* ≤ *n* ^*α*^, and the upper bound

(1.7) $$1-F_{d,n}(x)\leq c_{2}x^{-(\tau -1-\delta )}$$

holds for all *x* ≥ 1.

There are two examples that explain Condition 1.2. Consider the case of i.i.d. degrees with $P\left(D_{i}>x\right)=cx^{-(\tau -1)}$, then the degree sequence satisfies Condition 1.2 a.s. A second case is when the number of vertices of degree *k* is *n*_*k*_ = ⌈*nF*(*k*)⌉ − ⌈*nF*(*k* − 1)⌉, and 1 − *F*(*x*) = *cx* ^−(*τ* − 1)^. Condition 1.2 allows for more flexible degree sequences than just these examples.

If we fix $\beta <\min\{\alpha ,\frac{1}{\tau -1+\delta }\}$, the lower bound (1.6) ensures that the number of vertices of degree higher than *x* = *n* ^*β*^ is at least *n* ^1 − *β*(*τ* − 1 + *δ*)^, which diverges as a positive power of *n*. If we take $\beta >\frac{1}{2}$, these vertices with high probability form a complete graph. This will be essential for proving our main results. The precise value of *β* is irrelevant in the sequel of this paper.

For an asymptotic degree distribution with asymptotic probability mass function (*p*_*k*_)_*k* ∈ ℕ_, we say that

(1.8) $$d_{\min}=\min\left\{k\in N:p_{k}>0\right\}$$

is the minimal degree of the probability given by (*p*_*k*_)_*k* ∈ ℕ_. With these technical requests, we can state the main result for the configuration model:

Theorem 1.3 (Diameter of CMn for τ ∈ (2,3))Let ***d*** be a sequence satisfying Condition 1.1 with asymptotic degree distribution (*p*_*k*_)_*k*_ with *d*_min_ ≥ 3. Suppose that ***d*** satisfies Condition 1.2 with *τ* ∈ (2,3) and *d*_*i*_ ≥ *d*_min_ for all *i* ∈ [*n*]. Then

(1.9) $$\frac{\text{diam}({\text{CM}}_{n})}{\log\log n}\overset{P}{\underset{\;n\to \infty \;}{\to }}\;\frac{2}{\log(d_{\min}-1)}+\frac{2}{\left|\log(\tau -2)\right|},$$

where $\overset{P}{\underset{\;n\to \infty \;}{\to }}\;$ denotes convergence in probability as *n*→*∞*.

In fact, the result turns out to be false when *p*_1_ + *p*_2_ > 0, as shown by Fernholz and Ramachandran 12 (see also van der Hofstad and coworkers 17), since then there are long strings of vertices with low degrees that are of logarithmic length.

### 1.2 Preferential attachment model and main result

The configuration model presented in the previous section is a static model, because the size *n* ∈ ℕ of the graph was fixed.

The preferential attachment model instead is a dynamic model, because, in this model, vertices are added sequentially with a number of edges connected to them. These edges are attached to a receiving vertex with a probability proportional to the degree of the receiving vertex at that time plus a constant, thus favoring vertices with high degrees.

The idea of the preferential attachment model is simple, and we start by defining it informally. We start with a single vertex with a self‐loop, which is the graph at time 1. At every time *t* ≥ 2, we add a vertex to the graph. This new vertex has an edge incident to it, and we attach this edge to a random vertex already present in the graph, with probability proportional to the degree of the receiving vertex plus a constant *δ*, which means that vertices with large degrees are favored. Clearly, at each time *t* we have a graph of size *t* with exactly *t* edges.

We can modify this model by changing the number of edges incident to each new vertex we add. If we start at time 1 with a single vertex with *m* ∈ ℕ self loops, and at every time *t* ≥ 2 we add a single vertex with *m* edges, then at time *t* we have a graph of size *t* but with *mt* edges, that we call PA_*t*_(*m*,*δ*). When no confusion can arise, we omit the arguments (*m*,*δ*) and abbreviate PA_*t*_ = PA_*t*_(*m*,*δ*). We now give the explicit expression for the attachment probabilities.

Definition 1.4 (Preferential attachment model) Fix m ∈ ℕ, δ ∈ ( − m,∞). Denote by $\left\{t\overset{j}{\to }v\right\}$ the event that the jth edge of vertex t ∈ ℕ is attached to vertex v ∈ [t] (for 1 ≤ j ≤ m). The preferential attachment model with parameters (m,δ) is defined by the attachment probabilities

(1.10) $$P\left(\left.t\overset{j}{\to }v\;\right|{\text{PA}}_{t,j-1}\right)=\left\{\begin{array}{cc} \frac{D_{t,j-1}(v)+1+j\delta /m}{c_{t,j}}\; & \text{for}\;v=t,\\ \frac{D_{t,j-1}(v)+\delta }{c_{t,j}}\; & \text{for}\;v<t, \end{array}\right.$$

where PA_t,j − 1_ is the graph after the first *j* − 1 edges of vertex t have been attached, and correspondingly *D*_*t,j* − 1_(*v*) is the degree of vertex *v*. The normalizing constant *c*_*t,j*_ in (1.10) is

(1.11) $$c_{t,j}:=\left[m(t-1)+(j-1)\right]\left(2+\delta /m\right)+1+\delta /m.$$

We refer to Section 4.1 for more details and explanations on the construction of the model (in particular, for the reason behind the factor *jδ*/*m* in the first line of (1.10)).

Consider, as in (1.2), the empirical degree distribution of the graph, which we denote by *P*_*k*_(*t*), where in this case the degrees are random variables. It is known from the literature 5, 13 that, for every *k* ≥ *m*, as *t*→*∞*,

(1.12) $$P_{k}(t)\overset{P}{\underset{\;t\to \infty \;}{\to }}\;p_{k},$$

where *p*_*k*_∼*ck* ^−*τ*^, and *τ* = 3 + *δ*/*m*. We focus on the case *δ* ∈ ( − *m*,0), so that PA_*t*_ has a power‐law degree sequence with power‐law exponent *τ* ∈ (2,3).

For the preferential attachment model, our main result is the following:

Theorem 1.5 (Diameter of the preferential attachment model) Let (PA_*t*_)_*t* ≥ 1_ be a preferential attachment model with *m* ≥ 2 and *δ* ∈ ( − *m*,0). Then

(1.13) $$\frac{\text{diam}({\text{PA}}_{t})}{\log\log t}\overset{P}{\underset{\;t\to \infty \;}{\to }}\;\frac{2}{\log m}+\frac{4}{\left|\log(\tau -2)\right|},$$

where *τ* = 3 + *δ*/*m* ∈ (2,3).

In the proof of Theorem 1.5 we are also able to identify the typical distances in PA_*t*_:

Theorem 1.6 (Typical distance in the preferential attachment model) Let $V_{1}^{t}$ and $V_{2}^{t}$ be two independent uniform random vertices in [*t*]. Denote the distance between $V_{1}^{t}$ and $V_{2}^{t}$ in PA_*t*_ by *H*_*t*_. Then

(1.14) $$\frac{H_{t}}{\log\log t}\overset{P}{\underset{\;t\to \infty \;}{\to }}\;\frac{4}{\left|\log(\tau -2)\right|}.$$

Theorems 1.5 and 1.6 prove [17, Conjecture 1.8].

### 1.3 Structure of the paper and heuristics

The proofs of our main results on the diameter in Theorems 1.3 and 1.5 have a surprisingly similar structure. We present a detailed outline in Section 2 below, where we split the proof into a lower bound (Section 2.1) and an upper bound (Section 2.2) on the diameter. Each of these bounds is then divided into 3 statements, that hold for each model. In Sections 3 and 4 we prove the lower bound for the configuration model and for the preferential attachment model, respectively, while in Sections 5 and 6 we prove the corresponding upper bounds. In Caravenna and coworkers [6, Appendix], some proofs of technical results that are minor modifications of proofs in the literature are presented in detail.

Even though the configuration and preferential attachment models are quite different in nature, they are *locally* similar, because for both models the attachment probabilities are roughly proportional to the degrees. The core of our proof is a combination of *conditioning arguments* (which are particularly subtle for the preferential attachment model), that allow to combine local estimates in order to derive bounds on *global* quantities, such as the diameter.

Let us give a heuristic explanation of the proof (see Figure 1 for a graphical representation). For a quantitative outline, we refer to Section 2. We write PA_*n*_ instead of PA_*t*_ to simplify the exposition, and denote by *d*_fwd_ the minimal *forward degree*, that is *d*_fwd_ = *d*_min_ − 1 for the configuration model and *d*_fwd_ = *m* for the preferential attachment model.

- For the *lower bound* on the diameter, we prove that there are so‐called *minimally connected* vertices. These vertices are quite special, in that their neighborhoods up to distance $k_{n}^{-}\approx \log\log n/\log d_{\text{fwd}}$ are *trees with the minimal possible degree*, given by *d*_fwd_ + 1. This explains the first term in the right hand sides of (1.9) and (1.13).
  **Figure 1:**
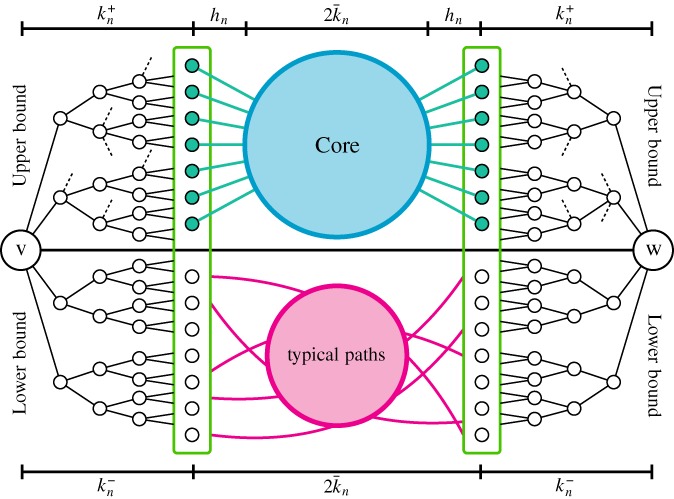
Structure of the proof in a picture [Colour figure can be viewed at wileyonlinelibrary.com]
  Pairs of minimally connected vertices are good candidates for achieving the maximal possible distance, that is, the diameter. In fact, the boundaries of their tree‐like neighborhoods turn out to be at distance equal to the *typical distance* $2{\bar{k}}_{n}$ between vertices in the graph, that is $2{\bar{k}}_{n}\approx 2c_{\text{dist}}\log\log n/|\log(\tau -2)|$, where *c*_dist_ = 1 for the configuration model and *c*_dist_ = 2 for the preferential attachment model. This leads to the second term in the right hand sides of (1.9) and (1.13).
  In the proof, we split the possible paths between the boundaries of two minimally connected vertices into bad paths, which are too short, and typical paths, which have the right number of edges in them, and then show that the contribution due to bad paths vanishes. The degrees along the path determine whether a path is bad or typical.
  The strategy for the lower bound is depicted in the bottom part of Figure 1.
- For the *upper bound* on the diameter, we perform a lazy‐exploration from every vertex in the graph and realize that the neighborhood up to a distance $k_{n}^{+}$, which is roughly the same as $k_{n}^{-}$, contains at least *as many vertices as the tree‐like neighborhood of a minimally connected vertex*. All possible other vertices in this neighborhood are ignored.
  We then show that the vertices at the boundary of these lazy neighborhoods are with high probability *quickly* connected to the core, that is by a path of $h_{n}=o(\log\log n)$ steps. By *core* we mean the set of all vertices with large degrees, which is known to be highly connected, with a diameter close to $2{\bar{k}}_{n}$, similar to the typical distances (see van der Hofstad and coworkers 17 for the configuration model and Dommers and coworkers 9 for the preferential attachment model).
  The proof strategy for the upper bound is depicted in the top part of Figure 1.

### 1.4 Links to the literature and comments

This paper studies the diameter in CM_*n*_ and PA_*t*_ when the degree power‐law exponent *τ* satisfies *τ* ∈ (2,3), which means the degrees have finite mean but infinite variance. Both in (1.9) and (1.13), the explicit constant is the sum of two terms, one depending on *τ*, and the other depending on the minimal forward degree (see (2.2)), which is *d*_min_ − 1 for CM_*n*_ and *m* for PA_*t*_. We remark that the term depending on *τ* is related to the typical distances, while the other is related to the periphery of the graph.

There are several other works that have already studied typical distances and diameters of such models. van der Hofstad and coworkers 16 analyze typical distances in CM_*n*_ for *τ* ∈ (2,3), while van der Hofstad and coworkers 15 study *τ* > 3. They prove that for *τ* ∈ (2,3) typical distances are of order loglogn, while for *τ* > 3 is of order logn, and it presents the explicit constants of asymptotic growth. Van der Hofstad and coworkers 17 shows for *τ* > 2 and when vertices of degree 1 or 2 are present, that with high probability the diameter of CM_*n*_ is bounded from below by a constant times logn, while when *τ* ∈ (2,3) and the minimal degree is 3, the diameter is bounded from above by a constant times loglogn. van der Hofstad and Komjáthy 18 investigate typical distances for configuration models and *τ* ∈ (2,3) in great generality, extending the results in van der Hofstad and coworkers 17 beyond the setting of i.i.d. degrees. Interestingly, they also investigate the effect of truncating the degrees at $n^{\beta_{n}}$ for values of *β*_*n*_→0. It would be of interest to also extend our diameter results to this setting.

We significantly improve upon the result in van der Hofstad and coworkers 17 for *τ* ∈ (2,3). We do make use of similar ideas in our proof of the upper bound on the diameter. Indeed, we again define a core consisting of vertices with high degrees, and use the fact that the diameter of this core can be computed exactly (for a definition of the core, see (2.8)). The novelty of our current approach is that we quantify precisely how far the further vertex is from this core in the configuration model. It is a pair of such remote vertices that realizes the graph diameter.

Fernholz and Ramachandran 12 prove that the diameter of CM_*n*_ is equal to an explicit constant times logn plus o(logn) when *τ* ∈ (2,3) but there are vertices of degree 1 or 2 present in the graph, by studying the longest paths in the configuration model that are not part of the 2‐core (which is the part of the graph for which all vertices have degree at least 2). Since our minimal degree is at least 3, the 2‐core is whp the entire graph, and thus this logarithmic phase vanishes. Dereich and coworkers 10 prove that typical distances in PA_*t*_ are asymptotically equal to an explicit constant times loglogt, using path counting techniques. We use such path counting techniques as well, now for the lower bound on the diameters. Van der Hofstad 14 studies the diameter of PA_*t*_ when *m* = 1, and proves that the diameter still has logarithmic growth. Dommers and coworkers 9 prove an upper bound on the diameter of PA_*t*_, but the explicit constant is not sharp.

Again, we significantly improve upon that result. Our proof uses ideas from Dommers and coworkers 9, in the sense that we again rely on an appropriately chosen core for the preferential attachment model, but our upper bound now quantifies precisely how the further vertex is from this core, as for the configuration model, but now applied to the much harder preferential attachment model. CM_*n*_ and PA_*t*_ are two different models, in the sense that CM_*n*_ is a static model while PA_*t*_ is a dynamic model. It is interesting to notice that the main strategy to prove Theorems 1.3 and 1.5 is the same. In fact, all the statements formulated in Section 2 are general and hold for both models. Also the explicit constants appearing in (1.9) and (1.13) are highly similar, which reflects the same structure of the proofs. The differences consist in a factor 2 in the terms containing *τ* and in the presence of *d*_min_ − 1 and *m* in the remaining term. The factor 2 can be understood by noting that in CM_*n*_ pairs of vertices with high degree are likely to be at distance 1, while in PA_*t*_ they are at distance 2. The difference in *d*_min_ − 1 and *m* is due to the fact that *d*_min_ − 1 and *m* play the same role in the two models, that is, they are the minimal forward degree (or “number of children”) of a vertex that is part of a tree contained in the graph. We refer to Section 2 for more details.

While the structures of the proofs for both models are identical, the details of the various steps are significantly different. Pairings in the configuration model are uniform, making explicit computations easy, even when already many edges have been paired. In the preferential attachment model, on the other hand, the edge statuses are highly dependent, so that we have no rely on delicate conditioning arguments. These conditioning arguments are arguably the most significant innovation in this paper. This is formalized in the notion of factorizable events in Definition 4.4.

Typical distances and diameters have been studied for other random graphs models as well, showing loglog behavior. Bloznelis 1 investigates the typical distance in power‐law intersection random graphs, where such distance, conditioning on being finite, is of order loglogn, while results on diameter are missing. Chung and Lu 7, 8 present results respectively for random graphs with given expected degrees and Erdős and Rényi random graphs *G*(*n*,*p*), see also van den Esker, the last author and Hooghiemstra 11 for the rank‐1 setting. The setting of the configuration model with finite‐variance degrees is studied in Fernholz and Ramachandran 12. In Chung and Lu 8, they prove that for the power‐law regime with exponent *τ* ∈ (2,3), the diameter is Θ(logn), while typical distances are of order loglogn. This can be understood from the existence of a positive proportion of vertices with degree 2, creating long, but thin, paths. In 7, the authors investigate the different behavior of the diameter according to the parameter *p*.

An interesting open problem is the study of fluctuations of the diameters in CM_*n*_ and PA_*t*_ around the asymptotic mean, that is, the study of the difference between the diameter of the graph and the asymptotic behavior (for these two models, the difference between the diameter and the right multiple of loglogn). In 16, the authors prove that in graphs with i.i.d. power‐law degrees with *τ* ∈ (2,3), the difference Δ_*n*_ between the typical distance and the asymptotic behavior 2loglogn/|log(τ−2)| does not converge in distribution, even though it is *tight* (ie, for every *ϵ* > 0 there is *M* < *∞* such that $P(|\Delta_{n}|\leq M)>1-\epsilon$ for all *n* ∈ ℕ). These results have been significantly improved in van der Hofstad and Komjáthym 18.

In the literature results on fluctuations for the diameter of random graph models are rare. Bollobás in 3, and, later, Riordan and Wormald in 20 give precise estimates on the diameter of the Erdös‐Renyi random graph. It would be of interest to investigate whether the diameter has tight fluctuations around cloglogn for the appropriate *c*.

## 2 GENERAL STRUCTURE OF THE PROOFS

We split the proof of Theorems 1.3 and 1.5 into a lower and an upper bound. Remarkably, the strategy is the same for both models despite the inherent difference in the models. In this section we explain the strategy in detail, formulating general statements that will be proved for each model separately in the next sections.

Throughout this section, we assume that the assumptions of Theorems 1.3 and 1.5 are satisfied and, to keep unified notation, we denote the size of the preferential attachment model by *n* ∈ ℕ, instead of the more usual *t* ∈ ℕ.

Throughout the paper, we treat real numbers as integers when we consider graph distances. By this, we mean that we round real numbers to the closest integer. To keep the notation light and make the paper easier to read, we omit the rounding operation.

### 2.1 Lower bound

We start with the structure of the proof of the lower bound in (1.9) and (1.13). The key notion is that of a *minimally k‐connected vertex*, defined as a vertex whose *k*‐neighborhood (ie, the neighborhood up to distance *k*) is essentially *a regular tree with the smallest possible degree*, equal to *d*_min_ for the configuration model and to *m* + 1 for the preferential attachment model. Due to technical reasons, the precise definition of minimally *k*‐connected vertex is slightly different for the two models (see Definitions 3.2 and 4.2).

Henceforth we fix *ε* > 0 and define, for *n* ∈ ℕ,

(2.1) $$k_{n}^{-}=(1-\varepsilon )\frac{\log\log n}{\log(d_{\text{fwd}})},$$

where *d*_fwd_ denotes the *forward degree*, or “number of children”:

(2.2) $$d_{\text{fwd}}=\left\{\begin{array}{cc} d_{\min}-1\; & \text{for}\;{\text{CM}}_{n};\\ m\; & \text{for}\;{\text{PA}}_{n}. \end{array}\right.$$

Our first goal is to prove that the number of minimally $k_{n}^{-}$‐connected vertices is large enough, as formulated in the following statement:

Statement 2.1 (Moments of $M_{k_{n}^{-}}$)*Denote by* $M_{k_{n}^{-}}$ the number of minimally $k_{n}^{-}$‐connected vertices in the graph (either CM_*n*_ or PA_*n*_). Then, as *n*→*∞*,

(2.3) $$E\left[M_{k_{n}^{-}}\right]\to \infty ,\mathrm{Var}\left(M_{k_{n}^{-}}\right)=o\left(E{\left[M_{k_{n}^{-}}\right]}^{2}\right),$$

where Var(*X*): = E[*X* ^2^] − E[*X*]^2^ denotes the variance of the random variable *X*.

The proof for the preferential attachment model makes use of conditioning arguments. Indeed, we describe as much information as necessary to be able to bound probabilities that vertices are minimally *k* connected. Particularly in the variance estimate, these arguments are quite delicate, and crucial for our purposes.

The bounds in (2.3) show that $M_{k_{n}^{-}}\overset{\;P\;}{\underset{}{\to }}\;\infty$ as *n*→*∞*. This will imply that *there is a pair of minimally* $k_{n}^{-}$‐connected vertices with disjoint $k_{n}^{-}$‐*neighborhoods*, hence the diameter of the graph is at least $2k_{n}^{-}$, which explains the first term in (1.9) and (1.13). Our next aim is to prove that these minimally connected trees are typically at distance $2c_{\text{dist}}\log\log n/|\log(\tau -2)|$, where *c*_dist_ = 1 for the configuration model and *c*_dist_ = 2 for the preferential attachment model.

For this, let us now define

(2.4) $${\bar{k}}_{n}=(1-\varepsilon )\frac{c_{\text{dist}}\log\log n}{\left|\log(\tau -2)\right|},$$

where

(2.5) $$c_{\text{dist}}=\left\{\begin{array}{cc} 1\; & \text{for}\;{\text{CM}}_{n};\\ 2\; & \text{for}\;{\text{PA}}_{n}. \end{array}\right.$$

The difference in the definition of *c*_dist_ is due to fact that in CM_*n*_ vertices with high degree are likely at distance 1, while in PA_*n*_ are at distance 2. We explain the origin of this effect in more detail in the proofs.

It turns out that the distance between the $k_{n}^{-}$‐neighborhoods of two minimally $k_{n}^{-}$‐connected vertices is at least $2{\bar{k}}_{n}$. More precisely, we have the following statement:

Statement 2.2 (Distance between neighborhoods)Let $W_{1}^{n}$ and $W_{2}^{n}$ be two random vertices chosen independently and uniformly among the minimally $k_{n}^{-}$‐connected ones. Denoting by ${\tilde{H}}_{n}$ the distance between the $k_{n}^{-}$‐neighborhoods of $W_{1}^{n}$ and $W_{2}^{n},$ we have ${\tilde{H}}_{n}\geq 2{\bar{k}}_{n}$ with high probability.

It follows immediately from Statement 2.2 that the distance between the vertices $W_{1}^{n}$ and $W_{2}^{n}$ is at least $2k_{n}^{-}+2{\bar{k}}_{n}$, with high probability. This proves the lower bound in (1.9) and (1.13).

It is known from the literature that $2{\bar{k}}_{n}$, see (2.4), represents the *typical distance* between two vertices chosen independently and uniformly in the graph. In order to prove Statement 2.2, we collapse the $k_{n}^{-}$‐neighborhoods of $W_{1}^{n}$ and $W_{2}^{n}$ into single vertices and show that their distance is roughly equal to the typical distance $2{\bar{k}}_{n}$. This is a delicate point, because the collapsed vertices have a relatively large degree and thus *could* be closer than the typical distance. The crucial point why they are not closer is that the degree of the boundary only grows polylogarithmically. The required justification is provided by the next statement:

Statement 2.3 (Bound on distances)*Let us introduce the set*

(2.6) $$V_{n}:=\left\{\begin{array}{cc} \{v\in [n]:\;d_{v}\leq \log n\}\; & \text{for}\;{\text{CM}}_{n};\\ \{v\in [n]:\;v\geq \frac{n}{{\left(\log n\right)}^{2}}\}\; & \text{for}\;{\text{PA}}_{n}. \end{array}\right.$$

Then, denoting the distance in the graph of size *n* by dist_*n*_,

(2.7) $$\max_{a,b\in V_{n}}P\left({\text{dist}}_{n}(a,b)\leq 2{\bar{k}}_{n}\right)=O\left(\frac{1}{{\left(\log n\right)}^{2}}\right).$$

The proof of Statement 2.3 is based on *path counting techniques*. These are different for the two models, but the idea is the same: We split the possible paths between the vertices *a* and *b* into two sets, called good paths and bad paths. Here good means that the degrees of vertices along the path increase, but not too much. We then separately and directly estimate the contribution of each set. The details are described in the proof.

### 2.2 Upper bound

We now describe the structure of the proof for the upper bound, which is based on two key concepts: the core of the graph and the *k*‐exploration graph of a vertex.

We start by introducing some notation. First of all, fix a constant *σ* ∈ (1/(3 − *τ*),*∞*). We define Core_*n*_ as the set of vertices in the graph of size *n* with degree larger than ${\left(\log n\right)}^{\sigma }$. More precisely, denoting by *D*_*t*_(*v*) = *D*_*t*,*m*_(*v*) the degree of vertex *v* in the preferential attachment model after time *t*, that is, in the graph PA_*t*_ (see the discussion after (1.10)), we let

(2.8) $${\text{Core}}_{n}:=\left\{\begin{array}{cc} \{v\in [n]:d_{v}\geq {\left(\log n\right)}^{\sigma }\}\; & \text{for}\;{\text{CM}}_{n};\\ \{v\in [n]:D_{n/2}(v)\geq {\left(\log n\right)}^{\sigma }\}\; & \text{for}\;{\text{PA}}_{n}. \end{array}\right.$$

The fact that we evaluate the degrees at time *n*/2 for the PAM is quite crucial in the proof of Statement 2.4 below. In Section 6, we also give bounds on *D*_*n*_(*v*) for *v* ∈ Core_*n*_, as well as for *v*∉Core_*n*_, that show that the degrees cannot grow too much between time *n*/2 and *n*. The first statement, that we formulate for completeness, upper bounds the diameter of Core_*n*_ and is already known from the literature for both models:

Statement 2.4 Define *c*_dist_ as in (2.5). Then, for every *ε* > 0, with high probability

(2.9) $$\frac{\text{diam}({\text{Core}}_{n})}{\log\log n}\leq (1+\varepsilon )\frac{2c_{\text{dist}}}{\left|\log(\tau -2)\right|}.$$

Statement 2.4 for CM_*n*_ is van der Hofstad and coworkers [17, Proposition 3.1], for PA_*n*_ it is Dommers and coworkers [9, Theorem 3.1].

Next we bound the distance between a vertex and Core_*n*_. We define the *k*‐exploration graph of a vertex *v* as a suitable subgraph of its *k*‐neighborhood, built as follows: We consider the usual exploration process starting at *v*, but instead of exploring all the edges incident to a vertex, we only explore a *fixed* number of them, namely *d*_fwd_ defined in (2.2). (The choice of which edges to explore is a natural one, and it will be explained in more detail in the proofs.)

We stress that it is possible to explore vertices that have already been explored, leading to what we call a collision. If there are no collisions, then the *k*‐exploration graph is a tree. In presence of collisions, the *k*‐exploration graph is not a tree, and it is clear that every collision reduces the number of vertices in the *k*‐exploration graph.

Henceforth we fix *ε* > 0 and, in analogy with (2.1), we define, for *n* ∈ ℕ,

(2.10) $$k_{n}^{+}=\left(1+\varepsilon \right)\frac{\log\log n}{\log(d_{\text{fwd}})}.$$

Our second statement for the upper bound shows that the $k_{n}^{+}$‐exploration graph of *every* vertex in the graph either intersects Core_*n*_, or it has a bounded number of collisions:

Statement 2.5 (Bound on collisions) There is a constant *c* < *∞* such that, with high probability, the $k_{n}^{+}$‐exploration graph of *every* vertex in the graph has at most *c* collisions before hitting Core_*n*_. As a consequence, for some constant *s* > 0, the $k_{n}^{+}$‐exploration graph of *every* vertex in the graph either intersects Core_*n*_, or its boundary has cardinality at least

(2.11) $$s{\left(d_{\text{fwd}}\right)}^{k_{n}^{+}}={\left(\log n\right)}^{1+\varepsilon +o(1)}.$$

With a bounded number of collisions, the $k_{n}^{+}$‐exploration graph is not far from being a tree, which explains the lower bound (2.11) on the cardinality of its boundary. Having enough vertices on its boundary, the $k_{n}^{+}$‐exploration is likely to be connected to Core_*n*_ *fast*, which for our purpose means in o(loglogn) steps. This is the content of our last statement:

Statement 2.6 There are constants *B*,*C* < *∞* such that, with high probability, the $k_{n}^{+}$‐exploration graph of every vertex in the graph is at distance at most $h_{n}=\lceil B\log\log\log n+C\rceil$ from Core_*n*_.

The proof for this is novel. For example, for the configuration model, we grow the $k_{n}^{+}+h_{n}$ neighborhood of a vertex, and then show that there are so many half‐edges at its boundary that it is very likely to connect immediately to the core. The proof for the preferential attachment model is slightly different, but the conclusion is the same. This shows that the vertex is indeed at most at distance $k_{n}^{+}+h_{n}$ away from the core.

In conclusion, with high probability, the diameter of the graph is at most

$$(k_{n}^{+}+h_{n})+\text{diam}({\text{Core}}_{n})+(k_{n}^{+}+h_{n}),$$

which gives us the expressions in (1.9) and (1.13) and completes the proof of the upper bound.

## 3 LOWER BOUND FOR CONFIGURATION MODEL

In this section we prove Statements 2.1 to2.3 for the configuration model. By the discussion in Section 2.1, this completes the proof of the lower bound in Theorem 1.3.

In our proof, it will be convenient to choose a particular order to pair the half‐edges. This is made precise in the following remark:

Remark 3.1 (Exchangeability in half‐edge pairing) Given a sequence **d**=(d_1_,…,d_n_) such that ℓ_n_ = d_1_ + ⋯ + d_n_ is even, the configuration model CM_n_ can be built iteratively as follows:

- start with d_i_ half‐edges attached to each vertex i ∈ [n] = {1,2,…,n};
- choose an *arbitrary* half‐edge and pair it to a uniformly chosen half‐edge;
- choose an *arbitrary* half‐edge, among the *ℓ*_*n*_ − 2 that are still unpaired, and pair it to a uniformly chosen half‐edge; and so on.

The *order* in which the arbitrary half‐edges are chosen does not matter in the above, by exchangeability (see also [13, Chapter 7]).

### 3.1 Proof of Statement 2.1

With a slight abuse of notation (see (1.8)), in this section we set

$$d_{\min}=\min\{d_{1},\text{⋯},d_{n}\}.$$

Given a vertex *v* ∈ [*n*] and *k* ∈ ℕ, we denote the set of vertices at distance at most *k* from *v* (in the graph CM_*n*_) by *U*_≤*k*_(*v*) and we call it the *k‐neighborhood of v*.

Definition 3.2 (Minimally k‐connected vertex) For k ∈ ℕ_0_, a vertex v ∈ [n] is called *minimally k‐connected* when all the vertices in *U*_≤*k*_(*v*) have minimal degree, that is,

$$\begin{array}{ccc} d_{i}=d_{\min} & & \text{for all}\;i\in U_{\leq k}(v), \end{array}$$

*and furthermore there are no self‐loops, multiple edges or cycles in U_≤k_(v). Equivalently, v is minimally k‐connected when the graph U_≤k_(v) is a regular tree with degree d_min_*.

We denote the (random) set of minimally k‐connected vertices by $M_{k}\subseteq [n],$ and its cardinality by $M_{k}=|M_{k}|,$ that is,  M_k_ denotes the number of minimally k‐connected vertices.

Remark 3.3 (The volume of the k‐neighborhood of k‐minimally connected vertices) For a minimally k‐connected vertex v, since U_≤k_(v) is a tree with degree d_min_, the number of *edges* inside *U*_≤*k*_(*v*) equals (assuming *d*_min_ ≥ 2)

(3.1) $$i_{k}=\sum_{l=1}^{k}d_{\min}{\left(d_{\min}-1\right)}^{l-1}=\left\{\begin{array}{cc} d_{\min}\;k\; & \text{if}\;d_{\min}=2;\\ d_{\min}\frac{{\left(d_{\min}-1\right)}^{k}-1}{d_{\min}-2}\; & \text{if}\;d_{\min}\geq 3. \end{array}\right.$$

Moreover, the number of *vertices* inside *U*_≤*k*_(*v*) equals *i*_*k*_ + 1. By (3.1), it is clear why *d*_min_ > 2, or *d*_min_ ≥ 3, is crucial. Indeed, this implies that the volume of neighborhoods of minimally *k*‐connected vertices grows exponentially in *k*.

Remark 3.4 (Collapsing minimally k connected trees) By Remarks 3.1 and 3.3, conditionally on the event $\left\{v\in M_{k}\right\}$ that a given vertex v is minimally k‐connected, the random graph obtained from CM_n_ by collapsing U_≤k_(v) to a single vertex, called a, *is still a configuration model* with *n* − *i*_*k*_ vertices and with *ℓ*_*n*_ replaced by *ℓ*_*n*_ − 2*i*_*k*_, where the new vertex *a* has degree *d*_min_(*d*_min_ − 1)^*k*^.

Analogously, conditionally on the event $\left\{v\in M_{k},w\in M_{m},U_{\leq k}(v)\cap U_{\leq m}(w)=\emptyset \right\}$ that two given vertices v and w are minimally k and minimally m‐connected with *disjoint* neighborhoods, collapsing *U*_≤*k*_(*v*) and *U*_≤*m*_(*w*) to single vertices *a* and *b* yields again a configuration model with *n* − *i*_*k*_ − *i*_*m*_ vertices, where *ℓ*_*n*_ is replaced by *ℓ*_*n*_ − 2*i*_*k*_ − 2*i*_*m*_ and where the new vertices *a* and *b* have degrees equal to *d*_min_(*d*_min_ − 1)^*k*^ and *d*_min_(*d*_min_ − 1)^*m*^, respectively.

We denote the number of vertices of degree *k* in the graph by *n*_*k*_, that is,

(3.2) $$n_{k}=\sum_{i\in [n]}{⊮}_{\left\{d_{i}=k\right\}}.$$

We now study the first two moments of *M*_*k*_, where we recall that the total degree *ℓ*_*n*_ is defined by (1.1):

Proposition 3.5 (Moments of M_k_) Let CM_*n*_ be a configuration model such that *d*_min_ ≥ 2. Then, for all *k* ∈ ℕ,

(3.3) $$E[M_{k}]=n_{d_{\min}}\prod_{i=1}^{i_{k}}\frac{d_{\min}(n_{d_{\min}}-i)}{\ell_{n}-2i+1},$$

where *i*_*k*_ is defined in (3.1). When, furthermore, *ℓ*_*n*_ > 4*i*_*k*_,

(3.4) $$E[M_{k}^{2}]\leq E{\left[M_{k}\right]}^{2}+E[M_{k}]\left((i_{k}+1)+i_{2k}\;d_{\min}\;\frac{n_{d_{\min}}}{\ell_{n}-4i_{k}}\right).$$

Before proving Proposition 3.5, let us complete the proof of Statement 2.1 subject to it. We are working under the assumptions of Theorem 1.3, hence *d*_min_ ≥ 3 and the degree sequence ***d*** satisfies the degree regularity condition, Condition 1.1, as well as the polynomial distribution condition Condition 1.2 with exponent *τ* ∈ (2,3). Recalling (1.1)‐(1.2), we can write $n_{d_{\min}}=n\;p_{d_{\min}}^{\left(n\right)}$ and $\ell_{n}=n\;\sum_{k\in N}kp_{k}^{\left(n\right)}$, so that, as *n*→*∞*,

(3.5) $$n_{d_{\min}}=n\;p_{d_{\min}}(1+o(1)),\ell_{n}=n\;\mu (1+o(1)),\text{with}\;p_{d_{\min}}>0,\mu :=\sum_{k\in N}kp_{k}<\infty .$$

Recalling the definition (2.1) of $k_{n}^{-}$ and (3.1), for $k=k_{n}^{-}$,

(3.6) $$i_{k_{n}^{-}}=d_{\min}\frac{{\left(d_{\min}-1\right)}^{k_{n}^{-}}-1}{d_{\min}-2}=\frac{d_{\min}}{d_{\min}-2}{\left(\log n\right)}^{1-\varepsilon }(1+o(1)),\text{hence}\;i_{2k_{n}^{-}}=O({\left(\log n\right)}^{2(1-\varepsilon )}).$$

Bounding E[*M*_*k*_] ≤ *n*, it follows by (3.4) that

(3.7) $$\mathrm{Var}[M_{k_{n}^{-}}]\leq E[M_{k_{n}^{-}}]\left(O(i_{k_{n}^{-}})+O(i_{2k_{n}^{-}})\right)\leq n\;O({\left(\log n\right)}^{2(1-\varepsilon )})=n^{1+o(1)}.$$

On the other hand, applying (3.3), for some *c* ∈ (0,1) one has

(3.8) $$E[M_{k_{n}^{-}}]\geq n\;p_{d_{\min}}\;{\left(\frac{d_{\min}\;p_{d_{\min}}}{\mu }+o(1)\right)}^{i_{k_{n}^{-}}}\geq n\;p_{d_{\min}}\;c^{{\left(\log n\right)}^{1-\varepsilon }}=n^{1-o(1)}.$$

Relations (3.7) and (3.8) show that (2.3) holds, completing the proof of Statement 2.1. □

Remark 3.6 (Disjoint neighborhoods) Let us show that, with high probability, there are vertices $v,w\in M_{k_{n}^{-}}$ with $U_{\leq k_{n}^{-}}(v)\cap U_{\leq k_{n}^{-}}(w)=\emptyset$. We proceed by contradiction: fix $v\in M_{k_{n}^{-}}$ and assume that, for every vertex $w\in M_{k_{n}^{-}}$, one has $U_{\leq k_{n}^{-}}(v)\cap U_{\leq k_{n}^{-}}(w)\neq \emptyset$. Then, for any $w\in M_{k_{n}^{-}}$ there must exist *a self‐avoiding path from v to w of length* $\leq 2k_{n}^{-}$ which only visits vertices with degree d_min_ (recall that $U_{\leq k_{n}^{-}}(v)$ and $U_{\leq k_{n}^{-}}(w)$ are regular trees). However, for fixed *v*, the number of such paths is $O({\left(d_{\min}-1\right)}^{2k_{n}^{-}})=O({\left(\log n\right)}^{2(1-\epsilon )})$, see (2.1), while by Statement 2.1 the number of vertices $w\in M_{k_{n}^{-}}$ is much larger, since $M_{k_{n}^{-}}∼E[M_{k_{n}^{-}}]=n^{1-o(1)}$, see (3.8).

Proof of Proposition 3.5 To prove (3.3) we write

(3.9) $$M_{k}=\sum_{v\in [n]:\;d_{v}=d_{\min}}{⊮}_{\left\{v\in M_{k}\right\}},$$

and since every vertex in the sum has the same probability of being minimally *k*‐connected,

(3.10) $$E\left[M_{k}\right]=n_{d_{\min}}P(v\in M_{k}).$$

A vertex *v* with *d*_*v*_ = *d*_min_ is in $M_{k}$ when all the half‐edges in *U*_≤*k*_(*v*) are paired to half‐edges incident to distinct vertices having minimal degree, without generating cycles. By Remark 3.1, we can start pairing a half‐edge incident to *v* to a half‐edge incident to another vertex of degree *d*_min_. Since there are *n*_*d*__min_ − 1 such vertices, this event has probability

$$\frac{d_{\min}(n_{d_{\min}}-1)}{\ell_{n}-1}$$

We iterate this procedure, and suppose that we have already successfully paired (*i* − 1) couples of half‐edges; then the next half‐edge can be paired to a distinct vertex of degree *d*_min_ with probability

(3.11) $$\frac{d_{\min}(n_{d_{\min}}-i)}{\ell_{n}-2(i-1)-1}=\frac{d_{\min}(n_{d_{\min}}-i)}{\ell_{n}-2i+1}.$$

Indeed, every time that we use a half‐edge of a vertex of degree *d*_min_, we cannot use its remaining half‐edges, and every step we make reduces the total number of possible half‐edges by two. By (3.1), exactly *i*_*k*_ couples of half‐edges need to be paired for *v* to be minimally *k*‐connected, so that

(3.12) $$E[M_{k}]=n_{d_{\min}}P(v\in M_{k})=n_{d_{\min}}\prod_{i=1}^{i_{k}}\frac{d_{\min}(n_{d_{\min}}-i)}{\ell_{n}-2i+1}.$$

which proves (3.3). If *n*_*d*min_ ≤ *i*_*k*_ the right hand side vanishes, in agreement with the fact that there cannot be any minimally *k*‐connected vertex in this case (recall (3.1)).

To prove (3.4), we notice that

(3.13) $$E[M_{k}^{2}]=\sum_{v,w\in [n]:\;d_{v}=d_{w}=d_{\min}}P(v,w\in M_{k}).$$

We distinguish different cases: the *k*‐neighborhoods of *v* and *w* might be disjoint or they may overlap, in which case *w* can be included in *U*_≤*k*_(*v*) or not. Introducing the events

(3.14) $$A_{v,w}=\left\{U_{\leq k}(v)\cap U_{\leq k}(w)\neq \emptyset \right\},B_{v,w}=\left\{w\in U_{\leq k}(v)\right\},$$

we can write the right hand side of (3.13) as

(3.15) $$\sum_{\begin{array}{c} v,w\in [n]\\ d_{v}=d_{w}=d_{\min} \end{array}}[P\left(v,w\in M_{k},A_{v,w}^{c}\right)+P\left(v,w\in M_{k},A_{v,w},B_{v,w}\right)+P\left(v,w\in M_{k},A_{v,w},B_{v,w}^{c}\right)].$$

Let us look at the first term in (3.15). By Remarks 3.3 and 3.4, conditionally on $\left\{v\in M_{k}\right\}$, the probability of $\left\{w\in M_{k},A_{v,w}^{c}\right\}$ equals the probability that *w* is minimally *k*‐connected in a new configuration model, with *ℓ*_*n*_ replaced by *ℓ*_*n*_ − 2*i*_*k*_ and with the number of vertices with minimal degree reduced from *n*_*d*min_ to *n*_*d*min_ − (*i*_*k*_ + 1). Then, by the previous analysis (see (3.12)),

(3.16) $$P\left(v,w\in M_{k},A_{v,w}^{c}\right)=\prod_{i=1}^{i_{k}}\frac{d_{\min}(n_{d_{\min}}-i-i_{k}-1)}{\ell_{n}-2i-2i_{k}+1}\;P\left(v\in M_{k}\right).$$

By direct computation, the ratio in the right hand side of (3.16) is always maximized for *i*_*k*_ = 0 (provided *ℓ*_*n*_ ≥ 2*n*_*d*min_ − 3, which is satisfied since *ℓ*_*n*_ ≥ *d*_min_ *n*_*d*min_ ≥ 3*n*_*d*min_ by assumption). Therefore, setting *i*_*k*_ = 0 in the ratio and recalling (3.12), we get the upper bound

(3.17) $$P\left(v,w\in M_{k},A_{v,w}^{c}\right)\leq \left[\prod_{i=1}^{i_{k}}\frac{d_{\min}(n_{d_{\min}}-i)}{\ell_{n}-2i+1}\right]\;P(v\in M_{k})=P{\left(v\in M_{k}\right)}^{2}.$$

Since there are at most $n_{d_{\min}}^{2}$ pairs of vertices of degree *d*_min_, it follows from (3.17) that

(3.18) $$\sum_{\begin{array}{c} v,w\in [n]\\ d_{v}=d_{w}=d_{\min} \end{array}}P\left(v,w\in M_{k},A_{v,w}^{c}\right)\leq n_{d_{\min}}^{2}\;P{\left(v\in M_{k}\right)}^{2}=E{\left[M_{k}\right]}^{2},$$

which explains the first term in (3.4).

For the second term in (3.15), *v* and *w* are minimally *k*‐connected with overlapping neighborhoods, and *w* ∈ *U*_≤*k*_(*v*). Since $\left\{v,w\in M_{k}\right\}\cap A_{v,w}\cap B_{v,w}\subseteq \left\{v\in M_{k}\right\}\cap B_{v,w}$, we can bound

(3.19) $$\sum_{\begin{array}{c} v,w\in [n]\\ d_{v}=d_{w}=d_{\min} \end{array}}P\left(v,w\in M_{k},A_{v,w},B_{v,w}\right)\leq E[\sum_{v\in [n]:\;d_{v}=d_{\min}}{⊮}_{\left\{v\in M_{k}\right\}}\sum_{w\in [n]:\;d_{w}=d_{\min}}{⊮}_{B_{v,w}}],$$

and note that $\sum_{w\in [n]}{⊮}_{B_{v,w}}=\left|U_{\leq k}(v)\right|=i_{k}+1$, by Remark 3.3. Therefore

(3.20) $$\sum_{\begin{array}{c} v,w\in [n]\\ d_{v}=d_{w}=d_{\min} \end{array}}P\left(v,w\in M_{k},A_{v,w},B_{v,w}\right)\leq E[M_{k}]\;(i_{k}+1),$$

which explains the second term in (3.4).

For the third term in (3.15), *v* and *w* are minimally *k*‐connected vertices with overlapping neighborhoods, but *w*∉*U*_≤*k*_(*v*). This means that dist(*v*,*w*) = *l* + 1 for some *l* ∈ {*k*,…,2*k* − 1}, so that $U_{\leq k}(v)\cap U_{\leq l-k}(w)=\emptyset$ and, moreover, a half‐edge on the boundary of *U*_≤(*l* − *k*)_(*w*) is paired to a half‐edge on the boundary of *U*_≤*k*_(*v*), an event that we call *F*_*v*,*w*;*l*,*k*_. Therefore

(3.21) $$\left\{w\in M_{k}\right\}\cap A_{v,w}\cap B_{v,w}^{c}\subseteq \overset{2k-1}{\underset{l=k}{\cup }}\left\{w\in M_{l-k}\right\}\cap \{U_{\leq k}(v)\cap U_{\leq l-k}(w)=\emptyset \}\cap F_{v,w;l,k}.$$

and we stress that in the right hand side *w* is only minimally (*l* − *k*)‐connected (in case *l* = *k* this just means that *d*_*w*_ = *d*_min_). Then

(3.22) $$P\left(v,w\in M_{k},A_{v,w},B_{v,w}^{c}\right)\leq \sum_{l=k}^{2k-1}E\left[{⊮}_{\left\{v\in M_{k},w\in M_{l-k},U_{\leq k}(v)\cap U_{\leq l-k}(w)=\emptyset \right\}}{⊮}_{F_{v,w;l,k}}\right].$$

By Remark 3.4, conditionally on $\left\{v\in M_{k},w\in M_{l-k},U_{\leq k}(v)\cap U_{\leq l-k}(w)=\emptyset \right\}$, we can collapse *U*_≤*k*_(*v*) and *U*_≤*l* − *k*_(*w*) to single vertices *a* and *b* with degrees respectively *d*_min_(*d*_min_ − 1)^*k*^ and *d*_min_(*d*_min_ − 1)^*l* − *k*^, getting a new configuration model with *ℓ*_*n*_ replaced by *ℓ*_*n*_ − 2*i*_*k*_ − 2*i*_*l* − *k*_. Bounding the probability that a half‐edge of *a* is paired to a half‐edge of *b*, we get

(3.23) $$\begin{array}{ll} P(F_{v,w;l,k}\;|\;v\in M_{k}, & w\in M_{l-k},U_{\leq k}(v)\cap U_{\leq l-k}(w)=\emptyset )\;\\ & \leq \frac{d_{\min}{\left(d_{\min}-1\right)}^{k}d_{\min}{\left(d_{\min}-1\right)}^{l-k}}{\ell_{n}-2i_{k}-2i_{l-k}-1}\leq \frac{d_{\min}^{2}{\left(d_{\min}-1\right)}^{l}}{\ell_{n}-4i_{k}}, \end{array}$$

because *l* ≤ 2*k* − 1 and, consequently, *i*_*l* − *k*_ ≤ *i*_*k* − 1_ ≤ *i*_*k*_ − 1. Plugging (3.23) into (3.22), and then forgetting the event $\left\{w\in M_{l-k},U_{\leq k}(v)\cap U_{\leq l-k}(w)=\emptyset \right\}$, leads to

(3.24) $$\begin{array}{ll} \sum_{\begin{array}{c} v,w\in [n]\\ d_{v}=d_{w}=d_{\min} \end{array}}P\left(v,w\in M_{k},A_{v,w},B_{v,w}^{c}\right) & \leq \left(\sum_{l=k}^{2k-1}\frac{d_{\min}^{2}{\left(d_{\min}-1\right)}^{l}}{\ell_{n}-4i_{k}}\right)\sum_{\begin{array}{c} v,w\in [n]\\ d_{v}=d_{w}=d_{\min} \end{array}}P(v\in M_{k})\;\\ & \leq \frac{d_{\min}(d_{\min}-1)}{\ell_{n}-4i_{k}}\;i_{2k-1}\;n_{d_{\min}}\;E[M_{k}], \end{array}$$

where we have used the definition (3.1) of *i*_2*k* − 1_. Since (*d*_min_ − 1)*i*_2*k* − 1_ ≤ *i*_2*k*_, again by (3.1), we have obtained the third term in (3.4).

### 3.2 Proof of Statement 2.2

We recall that $W_{1}^{n}$ and $W_{2}^{n}$ are two independent random vertices chosen uniformly in $M_{k_{n}^{-}}$ (the set of minimally $k_{n}^{-}$‐connected vertices), assuming that $M_{k_{n}^{-}}\neq \emptyset$ (which, as we have shown, occurs with high probability). Our goal is to show that

(3.25) $$\lim_{n\to \infty }P(E_{n})=0,$$

where we set

(3.26) $$E_{n}:=\left\{\text{dist}(U_{\leq k_{n}^{-}}(W_{1}^{n}),U_{\leq k_{n}^{-}}(W_{2}^{n}))\leq 2{\bar{k}}_{n}\right\}=\left\{\text{dist}(W_{1}^{n},W_{2}^{n})\leq 2k_{n}^{-}+2{\bar{k}}_{n}\right\}.$$

We know from Statement 2.1 that as *n*→*∞*

(3.27) $$P\left(M_{k_{n}^{-}}\leq \frac{1}{2}E[M_{k_{n}^{-}}]\right)\leq P\left(\left|M_{k_{n}^{-}}-E[M_{k_{n}^{-}}]|>\frac{1}{2}E[M_{k_{n}^{-}}\right]\right)\leq \frac{\mathrm{Var}[M_{k_{n}^{-}}]}{\frac{1}{4}E{\left[M_{k_{n}^{-}}\right]}^{2}}=o(1).$$

Therefore,

(3.28) $$\begin{array}{ll} P(E_{n}) & =P\left(E_{n}\cap \{M_{k_{n}^{-}}>\frac{1}{2}E[M_{k_{n}^{-}}]\}\right)+o(1)\;\\ & =E\left[\sum_{v_{1},v_{2}\in [n]}{⊮}_{\left\{W_{1}^{n}=v_{1},W_{2}^{n}=v_{2}\right\}}{⊮}_{\left\{\text{dist}(v_{1},v_{2})\leq 2k_{n}^{-}+2{\bar{k}}_{n}\right\}}{⊮}_{\left\{M_{k_{n}^{-}}>\frac{1}{2}E[M_{k_{n}^{-}}]\right\}}\right]+o(1)\;\\ & \leq E\left[\sum_{v_{1},v_{2}\in [n]}\frac{{⊮}_{\left\{v_{1}\in M_{k_{n}^{-}},v_{2}\in M_{k_{n}^{-}}\right\}}}{M_{k_{n}^{-}}^{2}}{⊮}_{\left\{\text{dist}(v_{1},v_{2})\leq 2k_{n}^{-}+2{\bar{k}}_{n}\right\}}{⊮}_{\left\{M_{k_{n}^{-}}>\frac{1}{2}E[M_{k_{n}^{-}}]\right\}}\right]+o(1)\;\\ & \leq \sum_{v_{1},v_{2}\in [n]}\frac{P\left(v_{1},v_{2}\in M_{k_{n}^{-}},\text{dist}(v_{1},v_{2})\leq 2k_{n}^{-}+2{\bar{k}}_{n}\right)}{\frac{1}{4}E{\left[M_{k_{n}^{-}}\right]}^{2}}+o(1).\; \end{array}$$

In analogy with (3.14), we introduce the event

$$A_{v_{1},v_{2}}:=\{U_{\leq k_{n}^{-}}(v_{1})\cap U_{\leq k_{n}^{-}}(v_{2})\neq \emptyset \},$$

and show that it gives a negligible contribution. Recalling the proof of Proposition 3.5, in particular (3.20) and (3.24), the sum restricted to $A_{v_{1},v_{2}}$ leads precisely to the second term in the right hand side of (3.4):

(3.29) $$\begin{array}{ll} \sum_{v_{1},v_{2}\in [n]}\frac{P\left(v_{1},v_{2}\in M_{k_{n}^{-}},A_{v_{1},v_{2}}\right)}{\frac{1}{4}E{\left[M_{k_{n}^{-}}\right]}^{2}} & \leq \frac{E[M_{k_{n}^{-}}]\left((i_{k_{n}^{-}}+1)+i_{2k_{n}^{-}}\frac{d_{\min}\;n_{d_{\min}}}{\ell_{n}-4i_{k_{n}^{-}}}\right)}{\frac{1}{4}E{\left[M_{k_{n}^{-}}\right]}^{2}}\;\\ & =\frac{O(i_{k_{n}^{-}})+O(i_{2k_{n}^{-}})}{E[M_{k_{n}^{-}}]}=\frac{O({\left(\log n\right)}^{2})}{n^{1-o(1)}}=o(1), \end{array}$$

where we have used (3.6) and (3.8) (see also (3.5)).

We can thus focus on the event $A_{v_{1},v_{2}}^{c}=\{U_{\leq k_{n}^{-}}(v_{1})\cap U_{\leq k_{n}^{-}}(v_{2})=\emptyset \}$. By Remark 3.4,

(3.30) $$P\left(\text{dist}(v_{1},v_{2})\leq 2k_{n}^{-}+2{\bar{k}}_{n}|v_{1},v_{2}\in M_{k_{n}^{-}},A_{v_{1},v_{2}}^{c}\right)=\hat{P}\left(\text{dist}(a,b)\leq 2{\bar{k}}_{n}\right),$$

where $\hat{P}$ is the law of the new configuration model which results from collapsing the neighborhoods $U_{\leq k_{n}^{-}}(v_{1})$ and $U_{\leq k_{n}^{-}}(v_{2})$ to single vertices *a* and *b*, with degrees $d_{\min}{\left(d_{\min}-1\right)}^{k_{n}^{-}}=O(\log n)$ (recall (2.1)‐(2.2)). The degree sequence $\hat{d}$ of this new configuration model is a slight modification of the original degree sequence ***d***: two new vertices of degree O(logn) have been added, while $2(i_{k_{n}^{-}}+1)=O(\log n)$ vertices with degree *d*_min_ have been removed (recall (3.6)). Consequently $\hat{d}$ still satisfies the assumptions of Theorem 1.3, hence Statement 2.3 (to be proved in Section 3.3) holds for $\hat{P}$ and we obtain

(3.31) $$\hat{P}\left(\text{dist}(a,b)\leq 2{\bar{k}}_{n}\right)=o(1).$$

We are ready to conclude the proof of Statement 2.2. By (3.28)‐(3.29)‐(3.30),

$$\begin{array}{ll} P(E_{n}) & =\sum_{v_{1},v_{2}\in [n]}\frac{P\left(v_{1},v_{2}\in M_{k_{n}^{-}},\text{dist}(v_{1},v_{2})\leq 2k_{n}^{-}+2{\bar{k}}_{n},A_{v_{1},v_{2}}^{c}\right)}{\frac{1}{4}E{\left[M_{k_{n}^{-}}\right]}^{2}}+o(1)\;\\ & \leq \hat{P}\left(\text{dist}(a,b)\leq 2{\bar{k}}_{n}\right)\sum_{v_{1},v_{2}\in [n]}\frac{P\left(v_{1},v_{2}\in M_{k_{n}^{-}}\right)}{\frac{1}{4}E{\left[M_{k_{n}^{-}}\right]}^{2}}+o(1)\;\\ & =\hat{P}\left(\text{dist}(a,b)\leq 2{\bar{k}}_{n}\right)\frac{E[{\left(M_{k_{n}^{-}}\right)}^{2}]}{\frac{1}{4}E{\left[M_{k_{n}^{-}}\right]}^{2}}+o(1).\; \end{array}$$

Observe that $E[{\left(M_{k_{n}^{-}}\right)}^{2}]=E{\left[M_{k_{n}^{-}}\right]}^{2}+\mathrm{Var}(M_{k_{n}^{-}})=O(E{\left[M_{k_{n}^{-}}\right]}^{2})$, by the second relation in (2.3). Applying (3.31), it follows that $P(E_{n})=o(1)$, completing the proof of Statement 2.2. □

### 3.3 Proof of Statement 2.3

In this section, we give a self‐contained proof of Statement 2.3 for CM_*n*_, as used in the proof of Statement 2.2.

Given two vertices *a*,*b* ∈ [*n*], let $P_{k}(a,b)$ be the set of all self‐avoiding paths of length *k* from *a* to *b*, that is of all sequences (*π*_0_,*π*_1_,…,*π*_*k*_) ∈ [*n*]^*k* + 1^ with *π*_0_ = *a*, *π*_*k*_ = *b* and such that (*π*_*i* − 1_,*π*_*i*_) is an edge in the graph, for all *i* = 1,…,*k*. Analogously, let $P_{k}(a)=\cup_{b\in [n]}P_{k}(a,b)$ denote the set of all paths of length *k* starting at *a*.

Let us fix an arbitrary increasing sequence ${\left(g_{l}\right)}_{l\in N_{0}}$ (that will be specified later). Define, for *a*,*b* ∈ R, a∧b:=min{a,b}. We say that a path $\pi \in P_{k}(a,b)$ is good when $d_{\pi_{l}}\leq g_{l}\wedge g_{k-l}$ for every *l* = 0,…,*k*, and bad otherwise. In other words, a path is good when the degrees along the path do not increase too much from *π*_0_ to *π*_*k*/2_, and similarly they do not increase too much in the backward direction, from *π*_*k*_ to *π*_*k*/2_.

For *k* ∈ ℕ_0_, we introduce the event

(3.32) $$E_{k}(a,b)=\left\{\exists \pi \in P_{k}(a,b):\;\pi \;\text{is a good path}\right\}.$$

To deal with bad paths, we define

(3.33) $$F_{k}(a)=\left\{\exists \pi \in P_{k}(a):\;d_{\pi_{k}}>g_{k}\;\text{but}\;d_{\pi_{i}}\leq g_{i}\;\forall i\leq k-1\right\}.$$

If ${\text{dist}}_{{\text{CM}}_{n}}(a,b)\leq 2\bar{k}$, then there must be a path in $P_{k}(a,b)$ for some $k\leq \bar{k}$, and this path might be good or bad. This leads to the simple bound

(3.34) $$P({\text{dist}}_{{\text{CM}}_{n}}(a,b)\leq 2\bar{k})\leq \sum_{k=0}^{2\bar{k}}P(E_{k}(a,b))+\sum_{k=0}^{\bar{k}}\left[P(F_{k}(a))+P(F_{k}(b))\right].$$

We give explicit estimates for the two sums in the right hand side. We introduce the *size‐biased distribution function* $F_{n}^{*}$ associated to the degree sequence ***d***=(*d*_1_,…,*d*_*n*_) by

(3.35) $$F_{n}^{*}(t)=\frac{1}{\ell_{n}}\sum_{v\in [n]}d_{v}\;{⊮}_{\left\{d_{v}\leq t\right\}}.$$

If we choose uniformly one of the *ℓ*_*n*_ half‐edges in the graph, and call $D_{n}^{*}$ the degree of the vertex incident to this half‐edge, then $F_{n}^{*}(t)=P(D_{n}^{*}\leq t)$. We also define the truncated mean

(3.36) $$\nu_{n}(t)=E[(D_{n}^{*}-1){⊮}_{\left\{D_{n}^{*}\leq t\right\}}]=\frac{1}{\ell_{n}}\sum_{v\in [n]}d_{v}(d_{v}-1){⊮}_{\left\{d_{v}\leq t\right\}}.$$

Now we are ready to bound (3.34).

Proposition 3.7 (Path counting for configuration model) Fix ***d***=(*d*_1_,…,*d*_*n*_) (such that *ℓ*_*n*_ = *d*_1_ + … + *d*_*n*_ is even) and an increasing sequence ${\left(g_{l}\right)}_{l\in N_{0}}$. For all distinct vertices *a*,*b* ∈ [*n*] with *d*_*a*_ ≤ *g*_0_, *d*_*b*_ ≤ *g*_0_, and for all $\bar{k}\in N,$

(3.37) $$\begin{array}{ll} P\left({\text{dist}}_{{\text{CM}}_{n}}(a,b)\leq 2\bar{k}\right)\leq & \frac{d_{a}d_{b}}{\ell_{n}}\sum_{k=1}^{2\bar{k}}(1-\frac{2k}{\ell_{n}}{\;)}^{-k}\;\prod_{l=1}^{k-1}\nu_{n}(g_{l}\wedge g_{h-l})\;\\ & +(d_{a}+d_{b})\sum_{k=1}^{\bar{k}}(1-\frac{2k}{\ell_{n}}{\;)}^{-k}\;(1-F_{n}^{*}(g_{k}))\prod_{l=1}^{k-1}\nu_{n}(g_{l}).\; \end{array}$$

Fix an arbitrary sequence of vertices *π* = (*π*_*i*_)_0 ≤ *i* ≤ *k*_ ∈ [*n*]^*k* + 1^. The probability that vertex *π*_0_ is connected to *π*_1_ is at most

$$\frac{d_{\pi_{0}}d_{\pi_{1}}}{\ell_{n}-1},$$

because there are $d_{\pi_{0}}d_{\pi_{1}}$ ordered couples of half‐edges, each of which can be paired with probability 1/(*ℓ*_*n*_ − 1) (recall Remark 3.1), and we use the union bound. By similar arguments, conditionally on a specific half‐edge incident to *π*_0_ being paired to a specific half‐edge incident to *π*_1_, the probability that another half‐edge incident to *π*_1_ is paired to a half‐edge incident to *π*_2_ is by the union bound bounded from above by

$$\frac{(d_{\pi_{1}}-1)d_{\pi_{2}}}{\ell_{n}-3}.$$

Iterating the argument, the probability that *π* is a path in CM_*n*_ is at most

(3.38) $$\frac{d_{\pi_{0}}d_{\pi_{1}}}{\ell_{n}-1}\frac{(d_{\pi_{1}}-1)d_{\pi_{2}}}{\ell_{n}-3}\frac{(d_{\pi_{2}}-1)d_{\pi_{3}}}{\ell_{n}-5}\text{⋯}\frac{(d_{\pi_{k-1}}-1)d_{\pi_{k}}}{\ell_{n}-(2k-1)}.$$

Let us now fix *a*,*b* ∈ [*n*] with *a* ≠ *b*. Recalling (3.32)‐(3.36), choosing *π*_0_ = *a*, *π*_*k*_ = *b* and summing (3.38) over all vertices *π*_1_,…,*π*_*k* − 1_ satisfying $d_{\pi_{i}}\leq g_{i}\wedge g_{k-i}$ yields

(3.39) $$P(E_{k}(a,b))\leq d_{a}d_{b}\frac{(\ell_{n}-2k-1)!!}{(\ell_{n}-1)!!}\left(\prod_{i=1}^{k-1}\ell_{n}\;\nu_{n}(g_{i}\wedge g_{k-i})\right).$$

Bounding (*ℓ*_*n*_ − 2*k* − 1)!!/(*ℓ*_*n*_ − 1)!! ≤ (*ℓ*_*n*_ − 2*k*)^−*k*^ yields the first term in the right hand side of (3.37). The bound for $P(F_{k}(a))$ is similar. Recalling (3.33)‐(3.35), choosing *π*_0_ = *a* and summing (3.38) over vertices *π*_1_,…,*π*_*k* − 1_,*π*_*k*_ such that $d_{\pi_{i}}\leq g_{i}$ for *i* ≤ *k* − 1 while $d_{\pi_{k}}>g_{k}$ gives

(3.40) $$P(F_{k}(a))\leq d_{a}\frac{(\ell_{n}-2k-1)!!}{(\ell_{n}-1)!!}\left(\prod_{i=1}^{k-1}\ell_{n}\;\nu_{n}(g_{i})\right)\{\ell_{n}\;(1-F_{n}^{*}(g_{k}))\},$$

and the same holds for $P(F_{k}(b))$. Plugging (3.39) and (3.40) into (3.34) proves (3.37).

In order to exploit (3.37), we need estimates on $F_{n}^{*}$ and *ν*_*n*_, provided by the next lemma:

Lemma 3.8 (Tail and truncated mean bounds for $F_{n}^{*}$) Assume that Condition 1.2 holds. Fix *η* > 0, then there exist two constants *C*_1_ = *C*_1_(*η*) and *C*_2_ = *C*_2_(*η*) such that, for every *x* ≥ 0,

(3.41) $$1-F_{n}^{*}(x)\leq C_{1}x^{-(\tau -2-\eta )},\;\nu_{n}(x)\leq C_{2}x^{\left(3-\tau +\eta \right)}.$$

For every *x* ≥ 0 and *t* ≥ 0 we can see that

(3.42) $$1-F_{n}^{*}(x)=\frac{1}{\ell_{n}}\sum_{v\in [n]}d_{v}{⊮}_{\left\{d_{v}>x\right\}}=\frac{n}{\ell_{n}}\left[\frac{1}{n}\sum_{v\in [n]}d_{v}{⊮}_{\left\{d_{v}>x\right\}}\right]=\frac{n}{\ell_{n}}E\left[D_{n}{⊮}_{\left\{D_{n}>x\right\}}\right],$$

where we recall that *D*_*n*_ is the degree of a uniformly chosen vertex. This means that

(3.43) $$\begin{array}{ll} \frac{n}{\ell_{n}}E[D_{n}{⊮}_{\left\{D_{n}>x\right\}}]= & \frac{n}{\ell_{n}}\sum_{j=0}^{\infty }P\left(D_{n}{⊮}_{\left\{D_{n}>x\right\}}>j\right)=\frac{n}{\ell_{n}}\sum_{j=0}^{\infty }P\left(D_{n}>j,D_{n}>x\right)\;\\ & =\frac{n}{\ell_{n}}\sum_{j=0}^{\infty }P\left(D_{n}>j\vee x\right)=\frac{n}{\ell_{n}}\sum_{j=0}^{\infty }(1-F_{d,n}(j\vee x))\;\\ & =\frac{n}{\ell_{n}}\left[x(1-F_{d,n}(x))+\sum_{j=x}^{\infty }(1-F_{d,n}(j))\right]\;\\ & \leq \frac{n}{\ell_{n}}C\left[x^{-(\tau -2-\eta )}+\sum_{j=x}^{\infty }j^{-(\tau -1-\eta )}\right]\leq C_{1}x^{-(\tau -2-\eta )}, \end{array}$$

where we have used Condition 1.2 in the second last step (recall that 2 < *τ* < 3).

For *ν*_*n*_, we can instead write

(3.44) $$\begin{array}{ll} \nu_{n}(x) & =\frac{1}{\ell_{n}}\sum_{v\in [n]}d_{v}(d_{v}-1){⊮}_{\left\{d_{v}\leq x\right\}}=\frac{n}{\ell_{n}}\left[\frac{1}{n}\sum_{v\in [n]}d_{v}(d_{v}-1){⊮}_{\left\{d_{v}\leq x\right\}}\right]\;\\ & =\frac{n}{\ell_{n}}E\left[D_{n}(D_{n}-1){⊮}_{\left\{D_{n}\leq x\right\}}\right]\leq \frac{n}{\ell_{n}}E\left[D_{n}^{2}{⊮}_{\left\{D_{n}\leq x\right\}}\right], \end{array}$$

where *D*_*n*_ is again the degree of a uniformly chosen vertex. The claim now follows from

(3.45) $$\begin{array}{ll} \frac{n}{\ell_{n}}E\left[D_{n}^{2}{⊮}_{\left\{D_{n}\leq x\right\}}\right] & =\frac{n}{\ell_{n}}\sum_{j=0}^{\infty }(2j+1)P\left(D_{n}{⊮}_{\left\{D_{n}\leq x\right\}}>j\right)\;\\ & =\frac{n}{\ell_{n}}\sum_{j=0}^{\infty }(2j+1)P\left(D_{n}>j,D_{n}\leq x\right)\leq \frac{n}{\ell_{n}}\sum_{j=0}^{x-1}(2j+1)P\left(D_{n}>j\right)\;\\ & =\frac{n}{\ell_{n}}\sum_{j=0}^{x-1}(2j+1)[1-F_{d,n}(j)]\leq \frac{n}{\ell_{n}}\sum_{j=0}^{x-1}Cj^{-(\tau -2-\eta )}\leq \frac{n}{\ell_{n}}C_{2}x^{3-\tau +\eta }.\; \end{array}$$

We are finally ready to complete the proof of Statement 2.3:

Proof of Statement 2.3 As in (2.4), we take

(3.46) $${\bar{k}}_{n}=(1-\varepsilon )\frac{\log\log n}{\left|\log(\tau -2)\right|},$$

and our goal is to show that, as *n*→*∞*,

(3.47) $$\max_{a,b\in [n]:\;d_{a},d_{b}\leq \log n}P\left({\text{dist}}_{{\text{CM}}_{n}}(a,b)\leq 2{\bar{k}}_{n}\right)\to 0.$$

We stress that *τ* ∈ (2,3) and *ε* > 0 are fixed. Then we choose *η* > 0 so small that

(3.48) $$2\eta <\tau -2\;\text{and}\;\frac{\left|\log(\tau -2-2\eta )\right|}{\left|\log|\log(\tau -2)\right|}\leq \frac{1-\varepsilon /2}{1-\varepsilon }.$$

We use the inequality (3.37) given by Proposition 3.7, with the following choice of ${\left(g_{k}\right)}_{k\in N_{0}}$:

(3.49) $$g_{k}:={\left(g_{0}\right)}^{p^{k}},\text{where}\;\left\{\begin{array}{c} g_{0}:={\left(\log n\right)}^{\log\log n};\;\\ p:=\frac{1}{\tau -2-2\eta }>1.\; \end{array}\right.$$

Let us focus on the first term in the right hand side of (3.37), that is

(3.50) $$\frac{d_{a}d_{b}}{\ell_{n}}\sum_{k=1}^{2\bar{k}}(1-\frac{2k}{\ell_{n}}{\;)}^{-k}\;\prod_{l=1}^{k-1}\nu_{n}(g_{l}\wedge g_{h-l}).$$

Since *ℓ*_*n*_ = *μn*(1 + *o*(1)) by (3.5), for $k\leq 2{\bar{k}}_{n}$ we have

(3.51) $$(1-\frac{2k}{\ell_{n}}{\;)}^{-k}\leq (1-\frac{4{\bar{k}}_{n}}{\ell_{n}}{\;)}^{-2{\bar{k}}_{n}}=1+O(\frac{\bar{k_{n}^{2}}}{\ell_{n}})=1+O(\frac{{\left(\log\log n\right)}^{2}}{n})=1+o(1).$$

Then observe that, by Lemma 3.8 and (3.49), for $k\leq 2{\bar{k}}_{n}$

(3.52) $$\begin{array}{ll} \prod_{l=1}^{k-1}\nu_{n}(g_{l}\wedge g_{k-l}) & =\prod_{l=1}^{k/2}\nu_{n}{\left(g_{l}\right)}^{2}\leq C_{2}^{k/2}\prod_{l=1}^{k/2}{\left(g_{l}\right)}^{2(3-\tau +\eta )}=C_{2}^{k/2}{\left(g_{0}\right)}^{2(3-\tau +\eta )\sum_{l=1}^{k/2}p^{l}}\;\\ & \leq C_{2}^{{\bar{k}}_{n}}{\left(g_{0}\right)}^{2(3-\tau +\eta )C\;p^{{\bar{k}}_{n}}}, \end{array}$$

with $C=\frac{p}{p-1}$. Note that $C_{2}^{{\bar{k}}_{n}}=O({\left(\log n\right)}^{c})$ for some *c* ∈ (0,*∞*), see (3.46), while by (3.48)

(3.53) $$p^{{\bar{k}}_{n}}=\exp(|\log(\tau -2-2\eta )|(1-\varepsilon )\frac{\log\log n}{\left|\log(\tau -2)|\right)})={\left(\log n\right)}^{(1-\varepsilon )\frac{\left|\log(\tau -2-2\eta )\right|}{\left|\log(\tau -2)\right|}}\leq {\left(\log n\right)}^{\left(1-\varepsilon /2\right)},$$

hence the right hand side of (3.52) is *n* ^*o*(1)^ (since $g_{0}={\left(\log n\right)}^{\log\log n}$). Then, for $d_{a},d_{b}\leq \log n$,

$$(3.63)\leq \frac{{\left(\log n\right)}^{2}}{\ell_{n}}(2{\bar{k}}_{n})\;(1+o(1))\;n^{o(1)}=O(\frac{{\left(\log n\right)}^{2}}{n}\;(\log\log n)\;n^{o(1)})=o(1).$$

It remains to look at the second sum in (3.37):

(3.54) $$(d_{a}+d_{b})\sum_{k=1}^{{\bar{k}}_{n}}(1-\frac{2k}{\ell_{n}}{\;)}^{-k}(1-F_{n}^{*}(g_{k}))\prod_{l=1}^{k-1}\nu_{n}(g_{l}).$$

By Lemma 3.8 ,we can bound $1-F_{n}^{*}(g_{k})\leq C_{1}{\left(g_{k}\right)}^{-(\tau -2-\eta )}$. By (3.51) and $C_{1}^{{\bar{k}}_{n}}=O({\left(\log n\right)}^{c})$ for some *c* ∈ (0,*∞*), see (3.46), bounding the product in (3.54) like we did in (3.52) yields

(3.55) $$O({\left(\log n\right)}^{c})\;(d_{a}+d_{b})\;\sum_{k=1}^{{\bar{k}}_{n}}{\left(g_{k}\right)}^{-(\tau -2-\eta )}{\left(g_{0}\right)}^{(3-\tau +\eta )Cp^{k-1}},$$

where *p* = 1/(*τ* − 2 − 2*η*) and $C=\frac{p}{p-1}$. By (3.49)

(3.56) $${\left(g_{k}\right)}^{-(\tau -2-\eta )}{\left(g_{0}\right)}^{-\frac{p}{p-1}(3-\tau +\eta )p^{k-1}}={\left(g_{k-1}\right)}^{-p(\tau -2-\eta )}{\left(g_{k-1}\right)}^{\frac{p}{p-1}(3-\tau +\eta )},$$

where

(3.57) $$p(\tau -2-\eta )=\frac{\tau -2-\eta }{\tau -2-2\eta }>1,\text{and}\;\frac{p}{p-1}(3-\tau +\eta )=\frac{3-\tau +\eta }{3-\tau +2\eta }<1.$$

This means that, setting $D\;:=\;p(\tau -2-\eta )-\frac{p}{p-1}(3-\tau +\eta )>0$, by (3.49),

(3.58) $$(3.69)=O({\left(\log n\right)}^{c})\;(d_{a}+d_{b})\;\sum_{k=1}^{{\bar{k}}_{n}}{\left(g_{0}\right)}^{-Dp^{k-1}}\leq O({\left(\log n\right)}^{c})\;\;\frac{d_{a}+d_{b}}{{\left(g_{0}\right)}^{D}}.$$

Since $g_{0}={\left(\log n\right)}^{\log\log n}$ while $d_{a},d_{b}\leq \log n$, the right hand side of (3.58) is *o*(1).

## 4 LOWER BOUND FOR PREFERENTIAL ATTACHMENT MODEL

In this section we prove Statements 2.1, 2.2 and 2.3 for the preferential attachment model. By the discussion in Section 2.1, this completes the proof of the lower bound in Theorem 1.5.

We recall that, given *m* ∈ ℕ and *δ* ∈ ( − *m*,*∞*), the preferential attachment model PA_*t*_ is a random graph with vertex set [*t*] = {1,2,…,*t*}, where each vertex *w* has *m* outgoing edges, which are attached to vertices *v* ∈ [*w*] with probabilities given in (1.10). In the next subsection we give a more detailed construction using random variables. This equivalent reformulation will be used in a few places, when we need to describe carefully some complicated events. However, for most of the exposition we will stick to the intuitive description given in Section 1.2.

### 4.1 Alternative construction of the preferential attachment model

We introduce random variables *ξ*_*w*,*j*_ to represent the vertex to which the *j*th edge of vertex *w* is attached, that is

(4.1) $$\xi_{w,j}=v\;\;\Leftrightarrow \;\;w\overset{j}{\to }v.$$

The graph PA_*t*_ is a *deterministic* function of these random variables: two vertices *v*,*w* ∈ [*t*] with *v* ≤ *w* are connected in PA_*t*_ if and only if *ξ*_*w*,*j*_ = *v* for some *j* ∈ [*m*]. In particular, the degree of a vertex *v* after the *k*th edge of vertex *t* has been attached, denoted by *D*_*t*,*k*_(*v*), is

(4.2) $$D_{t,k}(v):=\sum_{\left(s,i)\leq (t,k\right)}({⊮}_{\left\{\xi_{s,i}=v\right\}}+{⊮}_{\left\{s=v\right\}}),$$

where we use the natural order relation

(s,i)≤(t,j)⇔s<tors=t,i≤j.

Defining the preferential attachment model amounts to giving a joint law for the sequence *ξ* = (*ξ*_*w*,*j*_)_(*w*,*j*) ∈ ℕ × [*m*]_. In agreement with (1.10), we set *ξ*_1,*j*_ = 1 for all *j* ∈ [*m*], and for *t* ≥ 2

(4.3) $$P\left(\left.\xi_{t,j}=v\;\right|\xi_{\leq (t,j-1)}\right)=\left\{\begin{array}{cc} \frac{D_{t,j-1}(v)+1+j\delta /m}{c_{t,j}}\; & \text{if}\;v=t;\\ \frac{D_{t,j-1}(v)+\delta }{c_{t,j}}\; & \text{if}\;v<t, \end{array}\right.$$

where *ξ*_≤(*t*,*i* − 1)_ is a shorthand for the vector (*ξ*_*s*,*i*_)_(*s*,*i*) ≤ (*t*,*i* − 1)_ (and we agree that (*t*,0): = (*t* − 1,*m*)). The normalizing constant *c*_*t*,*j*_ in (4.3) is indeed given by (1.11), because by (4.2),

$$\sum_{v\in [t]}D_{t,j-1}(v)=\sum_{\left(s,i)\leq (t,j-1\right)}(1+1)=2((t-1)m+(j-1)).$$

The factor *jδ*/*m* in the first line of (4.3) is commonly used in the literature (instead of the possibly more natural *δ*). The reason is that, with such a definition, the graph PA_*t*_(*m*,*δ*) can be obtained from the special case *m* = 1, where every vertex has only one outgoing edge: one first generates the random graph PA_*mt*_(1,*δ*/*m*), whose vertex set is [*mt*], and then collapses the block of vertices [*m*(*i* − 1) + 1,*mi*) into a single vertex *i* ∈ [*t*] (see also [13, Chapter 8]).

Remark 4.1 It is clear from the construction that PA_t_ is a *labeled directed graph*, because any edge connecting sites *v*,*w*, say with *v* ≤ *w*, carries a label *j* ∈ [*m*] and a direction, from the newer vertex *w* to the older one *v* (see (4.1)). Even though our final result, the asymptotic behavior of the diameter, only depends on the underlying undirected graph, it will be convenient to exploit the labeled directed structure of the graph in the proofs.

### 4.2 Proof of Statement 2.1

We denote by *U*_≤*k*_(*v*) the *k*‐neighborhood in PA_*t*_ of a vertex *v* ∈ [*t*], that is the set of vertices at distance at most *k* from *v*, viewed as a labeled directed subgraph (see Remark 4.1). We denote by *D*_*t*_(*v*) = *D*_*t*,*m*_(*v*) the degree of vertex *v* after time *t*, that is, in the graph PA_*t*_ (recall (4.2)).

We define the notion of *minimally k‐connected vertex* in analogy with the configuration model (see Definition 3.2), up to minor technical restrictions made for later convenience.

Definition 4.2 (Minimally k‐connected vertex) For *k* ∈ ℕ_0_, a vertex v ∈ [t]∖[t/2] is called *minimally k‐connected* when *D*_*t*_(*v*) = *m*, all the other vertices *i* ∈ *U*_≤*k*_(*v*) are in [*t*/2]∖[*t*/4] and have degree *D*_*t*_(*i*) = *m* + 1, and there are no self‐loops, multiple edges or cycles in *U*_≤*k*_(*v*). The graph *U*_≤*k*_(*v*) is thus a tree with degree *m* + 1, except for the root *v* which has degree *m*.

We denote the (random) set of minimally *k*‐connected vertices by $M_{k}\subseteq [t]\setminus [t/2],$ and its cardinality by $M_{k}=|M_{k}|$.

For the construction of a minimally *k*‐connected neighborhood in the preferential attachment model we remind that the vertices are added to the graph at different times, so that the vertex degrees change while the graph grows. The relevant degree for Definition 4.2 is the one at the final time *t*. To build a minimally *k*‐connected neighborhood, we need

(4.4) $$i_{k}=1+\sum_{i=1}^{k}m^{i}=\frac{m^{k+1}-1}{m-1}$$

many vertices. The center *v* of the neighborhood is the youngest vertex in *U*_≤*k*_(*v*), and it has degree *m*, while all the other vertices have degree *m* + 1.

Our first goal is to evaluate the probability $P(v\in M_{k})$ that a given vertex *v* ∈ [*t*]∖[*t*/2] is minimally *k*‐connected. The analogous question for the configuration model could be answered quite easily in Proposition 3.5, because the configuration model can be built exploring its vertices in an arbitrary order, in particular starting from *v*, see Remark 3.1. This is no longer true for the preferential attachment model, whose vertices have an order, the chronological one, along which the conditional probabilities take the explicit form (1.10) or (4.3). This is why the proofs for the preferential attachment model are harder than for the configuration model.

As it will be clear in a moment, to get explicit formulas it is convenient to evaluate the probability $P(v\in M_{k},U_{\leq k}(v)=H)$, where *H* is a fixed *labeled directed* subgraph, that is, it comes with the specification of which edges are attached to which vertices. To avoid trivialities, we restrict to those *H* for which the probability does not vanish, that is, which satisfy the constraints in Definition 4.2, and we call them *admissible*.

Let us denote by *H* ^*o*^: = *H*∖*∂H* the set of vertices in *H* that are not on the boundary (ie, they are at distance at most *k* − 1 from *v*). With this notation, we have the following result:

Lemma 4.3 Let {PA_*t*_}_*t* ∈ ℕ_ be a preferential attachment model. For any vertex *v* ∈ [*t*]∖[*t*/2] and any directed labeled graph *H* which is admissible,

(4.5) $$P\left(v\in M_{k},U_{\leq k}(v)=H\right)=L_{1}(H)\;L_{2}(H),$$

where

(4.6) $$L_{1}(H):=\prod_{u\in H^{o}}\prod_{j=1}^{m}\frac{m+\delta }{c_{u,j}},$$

(4.7) $$L_{2}(H):=\prod_{u\notin H^{o}}\;\prod_{j=1}^{m}\left[1-\frac{D_{u-1}(H)+|H\cap [u-1]|\delta }{c_{u,j}}\right],$$

and $D_{u-1}(H)=\sum_{w\in H}D_{u-1,m}(w)$ is the total degree of *H* before vertex *u* is added to the graph, and the normalization constant *c*_*u*,*j*_ is defined in (1.11).

We recall that $\left\{a\overset{i}{\to }b\right\}$ denotes the event that the *i*th edge of *a* is attached to *b* (see (4.1)). Since *H* is an admissible labeled directed subgraph, for all *u* ∈ *H* ^*o*^ and *j* ∈ [*m*], the *j*th edge of *u* is connected to a vertex in *H*, that we denote by $\theta_{j}^{H}(u)$. We can then write

(4.8) $$\{v\in M_{k},U_{\leq k}(v)=H\}=(\underset{u\in H^{o}}{\cap }\overset{m}{\underset{j=1}{\cap }}\{u\overset{j}{\to }\theta_{j}^{H}(u)\})\cap (\underset{u\notin H^{o}}{\cap }\overset{m}{\underset{j=1}{\cap }}\{u\overset{j}{\to /}H\}),$$

where of course $\left\{u\overset{j}{\to /}H\}:=\cup_{w\notin H}\{u\overset{j}{\to }w\right\}$. The first term in (4.8) is exactly the event that the edges present in *H* are connected in PA_*t*_ as they should be. The second term is the event that the vertices *u*∉*H* ^*o*^ are not attached to *H*, so that *U*_≤*k*_(*v*) = *H*. Notice that in (4.8) every vertex and every edge of the graph appears. For a vertex *u* ∈ *H* ^*o*^, by (1.10)

(4.9) $$P(u\overset{j}{\to }\theta_{j}^{H}(u)|{\text{PA}}_{u,j-1})=\frac{m+\delta }{c_{u,j}},$$

because the vertex $\theta_{j}^{H}(u)$ has degree precisely *m* (when *u* is not already present in the graph). For *u*∉*H* ^*o*^, we have to evaluate the probability that its edges do no attach to *H*, which is

(4.10) $$P(u\overset{j}{\to /}H|{\text{PA}}_{u-1,j-1})=1-\frac{D_{u-1}(H)+|H\cap [u-1]|\delta }{c_{u,j}}.$$

Using conditional expectation iteratively, we obtain (4.9) or (4.10) for every edge in the graph, depending on whether the edge is part of *H* or not. This proves (4.6) and (4.7).

The event $\left\{v\in M_{k},U_{\leq k}(v)=H\right\}$ is an example of a class of events, called *factorizable*, that will be used throughout this section and Section 6. For this reason we define it precisely.

It is convenient to use the random variable *ξ*_*w*,*j*_, introduced in Section 4.1, to denote the vertex to which the *j*th edge of vertex *w* is attached (see (4.1)). Any event *A* for PA_*t*_ can be characterized iteratively, specifying a set *A*_*s*,*i*_⊆[*s*] of values for *ξ*_*s*,*i*_, for all (*s*,*i*) ≤ (*t*,*m*):

$$A=\underset{\left(s,i)\leq (t,m\right)}{\cap }\{\xi_{s,i}\in A_{s,i}\}.$$

Of course, the set *A*_*s*,*i*_ is allowed to depend on the “past,” that is,  *A*_*s*,*i*_ = *A*_*s*,*i*_(*ξ*_≤(*s*,*i* − 1)_), or equivalently *A*_*s*,*i*_ = *A*_*s*,*i*_(PA_*s*,*i* − 1_). Let us set $A_{\leq (s,i)}:=\cap_{\left(u,j)\leq (s,i\right)}A_{u,j}$.

Definition 4.4 (Factorizable events) An event A for PA_t_ is called *factorizable* when the conditional probabilities of the events {*ξ*_*s*,*i*_ ∈ *A*_*s*,*i*_}, given the past, are deterministic. More precisely, for any (*s*,*i*) there is a (non‐random) *p*_*s*,*i*_ ∈ [0,1] such that

(4.11) $$P\left(\left.\xi_{s,i}\in A_{s,i}\;\right|\xi_{\leq (s,i-1)}\right)=p_{s,i}$$

on the event ξ_≤(*s,i* − 1)_ ∈ A_≤(*s,i* − 1)_. As a consequence, the chain rule for probabilities yields

$$P(A)=\prod_{\left(s,i)\leq (t,m\right)}p_{s,i}.$$

Remark 4.5 Relations (4.9) and (4.10) show that $A=\{v\in M_{k},U_{\leq k}(v)=H\}$ is a factorizable event. In fact, A_*s,i*_ is either the single vertex $\theta_{i}^{H}(s)$ (if s ∈ H^o^) or the set [s − 1]∖H (if s∉H^o^). In both cases, the set A_*s,i*_⊆[s − 1] has *a fixed total degree and a fixed cardinality*, hence the conditional probabilities (4.11) are specified in a deterministic way (recall (4.3)).

Note that the event $\left\{v\in M_{k}\right\}$ is not factorizable. This is the reason for specifying the realization of the *k*‐neighborhood U_≤k_(v) = H.

Henceforth we fix *ε* > 0. We recall that $k_{n}^{-}$ was defined in (2.1). Using the more customary *t* instead of *n*, we have

(4.12) $$k_{t}^{-}=(1-\varepsilon )\frac{\log\log t}{\log m}.$$

We recall that $M_{k_{t}^{-}}=|M_{k_{t}^{-}}|$ denotes the number of minimally $k_{t}^{-}$‐connected vertices in PA_*t*_ (see Definition 4.2). We can now prove half of Statement 2.1 for the preferential attachment model, more precisely the first relation in Equation (2.3).

Proposition 4.6 First moment of $M_{k_{t}^{-}}$) Let (PA_*t*_)_*t* ≥ 1_ be a preferential attachment model, with *m* ≥ 2 and *δ* ∈ ( − *m*,0). Then, for $k_{t}^{-}$ as in (4.12), as *t*→*∞*,

(4.13) $$E[M_{k_{t}^{-}}]\to \infty .$$

Similarly to the proof of (3.3), we write

(4.14) $$E[M_{k}]=\sum_{v\in [t]\setminus [t(2]}P\left(v\in M_{k}\right)=\sum_{v\in [t]\setminus [t/2]}\sum_{H\subseteq [t]\setminus [t/4]}P(v\in M_{k},U_{\leq k}(v)=H),$$

where the sum is implicitly restricted to admissible *H* (ie, to *H* that are possible realizations of *U*_≤*k*_(*v*)).

Since we will use (4.5), we need a lower bound on (4.6) and (4.7). Recalling (1.11), it is easy to show, since the number of vertices in *H* ^*o*^ equals *i*_*k*_ − *m* ^*k*^ = *i*_*k* − 1_, and *u* ≤ *v* for *u* ∈ *H* ^*o*^,

(4.15) $$L_{1}(H)\geq {\left[\frac{m+\delta }{v(2m+\delta )+1+\delta /m}\right]}^{mi_{k-1}}.$$

Note that for *u* ≤ *t*/4 all the factors in the product in (4.7) equal 1, because *H*⊆[*t*]∖[*t*/4]. Restricting to *u* > *t*/4 and bounding $D_{u-1}(H)+|H\cap [u-1]|\delta \leq (m+1+\delta )i_{k}$, we get

(4.16) $$L_{2}(H)\geq {\left[1-\frac{(m+1+\delta )i_{k}}{\frac{t}{4}(2m+\delta )}\right]}^{3mt/4}.$$

Let us write $H=\{v\}\cup H^{\prime }$ where *H* ^*′*^ is a subset of [*t*/2]∖[*t*/4] with |*H* ^*′*^| = *i*_*k*_ − 1. Clearly, for any such subset there is at least one way to order the vertices to generate an admissible *H*. The number of possible subsets in [*t*/2]∖[*t*/4] is at least t/4ik−1. Then, we obtain

(4.17) E[Mk]≥∑v∈[t]∖[t/2]t/4ik−1m+δv(2m+δ)+1+δ/mmik−11−(m+1+δ)ikt4(2m+δ)3mt/4.

Recalling that

(4.18) t/4ik−1=tik4ik(ik−1)!(1+o(1)),

since *mi*_*k* − 1_ ≤ *i*_*k*_, we obtain

(4.19) $$E[M_{k}]\geq \frac{t}{2}\frac{t^{i_{k}}}{4^{i_{k}}(i_{k}-1)!}{\left[\frac{m+\delta }{t(2m+\delta )+1+\delta /m}\right]}^{i_{k}}{\left[1-\frac{(m+1+\delta )i_{k}}{\frac{t}{4}(2m+\delta )}\right]}^{3mt/4}.$$

Choosing $k=k_{t}^{-}$ as in (4.12) and bounding 1 − *x* ≥ e^−2*x*^ for *x* small, as well as *m* + 1 ≤ 2*m*, we obtain

(4.20) $$E[M_{k_{t}^{-}}]\geq \frac{t}{2}\;\frac{t^{i_{k_{t}^{-}}}}{4^{i_{k_{t}^{-}}}\;i_{k_{t}^{-}}!}{\left(\frac{m}{C\;t}\right)}^{i_{k_{t}^{-}}}\exp\left(-3\;c\;m\;i_{k_{t}^{-}}\right)\geq \frac{1}{{\left(C^{\prime }\right)}^{i_{k_{t}^{-}}}}\;\frac{t}{2\;i_{k_{t}^{-}}!}\;\exp\left(-3\;c\;m\;i_{k_{t}^{-}}\right),$$

where *C* is a constant and *C* ^*′*^ = 4*C*/*m*. Recalling that *i*_*k*_ is given by (4.4), and $k_{t}^{-}$ by (4.12), hence $i_{k_{t}^{-}}=\frac{m}{m-1}m^{k_{t}^{-}}(1+o(1))\leq 2{\left(\log t\right)}^{1-\varepsilon }$, hence

(4.21) $$i_{k_{t}^{-}}!\leq \lfloor 2{\left(\log t\right)}^{1-\varepsilon }\rfloor !\leq {\left[2{\left(\log t\right)}^{1-\varepsilon }\right]}^{2{\left(\log t\right)}^{1-\varepsilon }}=t^{o(1)},$$

and also ${\left(C^{\prime }e^{3\;c\;m}\right)}^{i_{k_{t}^{-}}}=t^{o(1)}$. This implies that 𝔼[*M*_*k*_]→*∞*, as required.

Remark 4.7 (Disjoint neighborhoods for minimally k‐connected pairs) We observe that, on the event $\left\{v,w\in M_{k}\right\}$ with v ≠ w, necessarily

$$U_{\leq k}(v)\cap U_{\leq k}(w)=\emptyset ,$$

because if a vertex x is in $U_{\leq k}(v)\cap U_{\leq k}(w)$ and x ≠ v,w, this means that *D*_*x*_(*t*) = *m* + 2, because in addition to its original m outgoing edges, vertex x has one incident edge from a younger vertex in *U*_≤k_(*v*) and one incident edge from a younger vertex in *U*_≤k_(*u*), which gives a contradiction. Similar arguments apply when *x* = *v* or *x* = *w*.

We use the previous remark to prove the second relation in Statement 2.1 for the preferential attachment model.

Proposition 4.8 Second moment of $M_{k_{t}^{-}}$) Let (PA_*t*_)_*t* ≥ 1_ be a preferential attachment model, with *m* ≥ 2 and *δ* ∈ ( − *m*,0). Then, for *k* ∈ ℕ,

(4.22) $$E[M_{k}^{2}]\leq \exp\left(32mi_{k}^{2}/t\right)E{\left[M_{k}\right]}^{2}+E[M_{k}].$$

Consequently, for $k=k_{t}^{-}$ as in (4.12), as *t*→*∞*,

(4.23) $$E[M_{k_{t}^{-}}^{2}]\leq (1+o(1))\;E{\left[M_{k_{t}^{-}}\right]}^{2}.$$

We write

(4.24) $$E\left[M_{k}^{2}\right]=\sum_{v,w\in [t]\setminus [t/2]}P\left(v,w\in M_{k}\right)=\sum_{v\neq w}P\left(v,w\in M_{k}\right)+E[M_{k}].$$

By Remark 4.7, for *v* ≠ *w* we can write

(4.25) $$P\left(v,w\in M_{k}\right)=\sum_{H_{v}\cap H_{w}=\emptyset }P\left(v,w\in M_{k},U_{\leq k}(v)=H_{v},U_{\leq k}(w)=H_{w}\right).$$

The crucial observation is that the event $\left\{v,w\in M_{k},U_{\leq k}(v)=H_{v},U_{\leq k}(w)=H_{w}\right\}$ is factorizable (recall Definition 4.4 and Remark 4.5). More precisely, in analogy with (4.6) and (4.7):

(4.26) $$P\left(v,w\in M_{k},U_{\leq k}(v)=H_{v},U_{\leq k}(w)=H_{w}\right)=L_{1}(H_{v},H_{w})L_{2}(H_{v},H_{w}),$$

where now

(4.27) $$L_{1}(H_{v},H_{w})=\prod_{x\in H_{v}^{o}\cup H_{w}^{o}}\prod_{j=1}^{m}\frac{m+\delta }{c_{x,j}},$$

(4.28) $$L_{2}(H_{v},H_{w})=\prod_{x\notin H_{v}^{o}\cup H_{w}^{o}}\prod_{j=1}^{m}\left[1-\frac{D_{x-1}(H_{v}\cup H_{w})+|(H_{v}\cup H_{w})\cap [x-1]|\delta }{c_{x,j}}\right].$$

To prove (4.26), notice that in (4.27) and (4.28) every edge and every vertex of the graph appear. Further, (4.27) is the probability of the event {*U*_≤*k*_(*v*) = *H*_*v*_,*U*_≤*k*_(*w*) = *H*_*w*_}, while (4.28) is the probability that all vertices not in the two neighborhoods do not attach to the two trees.

A look at (4.6) shows that *L*_1_(*H*_*v*_,*H*_*w*_) = *L*_1_(*H*_*v*_)*L*_1_(*H*_*w*_). We now show that analogous factorization holds approximately also for *L*_2_. Since, for every *a*,*b* ∈ [0,1], with *a* + *b* < 1, it is true that 1 − (*a* + *b*) ≤ (1 − *a*)(1 − *b*), we can bound

(4.29) $$\begin{array}{ll} & \left[1-\frac{D_{x-1}(H_{v}\cup H_{w})+|(H_{v}\cup H_{w})\cap [x-1]|\delta }{c_{x,j}}\right]\;\\ & \;\leq \left[1-\frac{D_{x-1}(H_{v})+|H_{v}\cap [x-1]|\delta }{c_{x,j}}\right]\left[1-\frac{D_{x-1}(H_{w})+|H_{w}\cap [x-1]|\delta }{c_{x,j}}\right].\; \end{array}$$

When we plug (4.29) into (4.28), we obtain *L*_2_(*H*_*v*_)*L*_2_(*H*_*w*_) (recall (4.7)) times the following terms:

(4.30) $${\left(\prod_{x\in H_{w}^{o}}\left[1-\frac{D_{x-1}(H_{v})+|H_{v}\cap [x-1]|\delta }{c_{x,j}}\right]\right)}^{-1}{\left(\prod_{x\in H_{v}^{o}}\left[1-\frac{D_{x-1}(H_{w})+|H_{w}\cap [x-1]|\delta }{c_{x,j}}\right]\right)}^{-1}.$$

We can bound $D_{x-1}(H_{v})+|H_{v}\cap [x-1]|\delta \leq D_{x-1}(H_{v})\leq (m+1)i_{k}$ (recall that *δ* < 0) and analogously for *H*_*w*_. The square brackets in (4.30) equal 1 for *x* ≤ *t*/4 (since *H*_*v*_,*H*_*w*_⊆[*t*]∖[*t*/4] by construction), and for *x* > *t*/4 we have $c_{x,j}\geq \frac{t}{4}(2m+\delta )\geq \frac{m}{4}t$ by (1.11) and *δ* > −*m*. We can thus write

(4.31) $$\begin{array}{ll} L_{2}(H_{v},H_{w}) & \leq L_{2}(H_{v})\;L_{2}(H_{w})\;\prod_{x\in H_{v}^{o}\cup H_{w}^{o}}\prod_{j=1}^{m}{\left[1-\frac{(m+1)i_{k}}{\frac{m}{4}t}\right]}^{-1}\;\\ & \leq L_{2}(H_{v})\;L_{2}(H_{w})\;\exp\left(2(2i_{k})m\frac{(m+1)i_{k}}{\frac{m}{4}t}\right), \end{array}$$

where we have used the bound 1 − *z* ≥ e^−2*z*^ for small *z* > 0. Since *m* + 1 ≤ 2*m*, we obtain

(4.32) $$\begin{array}{l} \sum_{v\neq w}\left[\sum_{H_{v}\cap H_{w}=\emptyset }P\left(v,w\in M_{k}\text{,}\;U_{\leq k}(v)=H_{v}\text{,}\;U_{\leq k}(w)=H_{w}\right)\right]\\ \;\leq \exp\left(32mi_{k}^{2}/t\right)\sum_{v\in [t]\setminus [t/2]}\sum_{H_{v}}L_{1}(H_{v})L_{2}(H_{v})\sum_{w\in [t]\setminus [t/2]}\sum_{H_{w}}L_{1}(H_{w})L_{2}(H_{w})\\ \;=\exp\left(32mi_{k}^{2}/t\right)E{\left[M_{k}\right]}^{2}. \end{array}$$

Substituting (4.32) in (4.24) completes the proof of (4.22).

Finally, for $k=k_{t}^{-}$ as in (4.12) we have $i_{k_{t}^{-}}\leq 2{\left(\log t\right)}^{1-\varepsilon }$ (recall that *i*_*k*_ is given by (4.4)). We have already shown in Proposition 4.6 that $E[M_{k_{t}^{-}}]\to \infty$, hence (4.23) follows.

Together, Propositions 4.6 and 4.8 prove Statement 2.1. This means, as for the configuration model, since $\mathrm{Var}(M_{k_{t}^{-}}^{2})=o(E{\left[M_{k_{t}^{-}}\right]}^{2})$, that $M_{k_{t}^{-}}/E[M_{k_{t}^{-}}]\overset{P}{\underset{\;t\to \infty \;}{\to }}1$, so in particular $M_{k_{t}^{-}}\overset{P}{\underset{\;t\to \infty \;}{\to }}\infty$.

### 4.3 Proof of Statement 2.3

Fix *ε* > 0 and define, as in (2.4),

(4.33) $${\bar{k}}_{t}=(1-\varepsilon )\frac{2\log\log t}{\left|\log(\tau -2)\right|}.$$

Statement 2.3 follows from the following result on distances between not too early vertices:

Proposition 4.9 (Lower bound on distances) Let (PA_*t*_)_*t* ≥ 1_ be a preferential attachment model, with *m* ≥ 2 and *δ* ∈ ( − *m*,0). Then, there exists a constant *p* > 0 such that

(4.34) $$\max_{x,y\geq \frac{t}{{\left(\log t\right)}^{2}}}P\left({\text{dist}}_{{\text{PA}}_{t}}(x,y)\leq 2{\bar{k}}_{t}\right)\leq \frac{p}{{\left(\log t\right)}^{2}}.$$

Inequality (4.34) is an adaptation of a result proved in [10, Section 4.1]. Consequently we just give a sketch of the proof (the complete proof can be found in [6, Appendix A]).

Let us denote by *u*↔*v* the event that vertices *u*,*v* are neighbors in PA_*t*_, that is

$$\{u↔v\}=\overset{m}{\underset{j=1}{\cup }}(\{u\overset{j}{\to }v\}\cup \{v\overset{j}{\to }u\}).$$

(As a matter of fact, $\left\{v\overset{j}{\to }u\right\}$ is only possible if *v* > *u*, while $\left\{u\overset{j}{\to }v\right\}$ is only possibly if *v* < *u*.) Given a sequence *π* = (*π*_0_,*π*_1_,…,*π*_*k*_) ∈ [*t*]^*k* + 1^ of distinct vertices, we denote by {*π*⊆PA_*t*_} the event that *π* is a path in PA_*t*_, that is

$$\{\pi \subseteq {\text{PA}}_{t}\}=\{\pi_{0}↔\pi_{1}↔\pi_{2}\text{⋯}↔\pi_{k}\}=\overset{k}{\underset{i=1}{\cap }}\{\pi_{i-1}↔\pi_{i}\}.$$

The proof of Proposition 4.9 requires the following bound on the probability of connection between two vertices from Dommers and coworkers [9, Lemma 2.2]: for $\gamma =m/(2m+\delta )\in (\frac{1}{2},1)$, there exists *c* ∈ (0,*∞*) such that, for all vertices *u*,*v* ∈ [*t*].

(4.35) $$P\left(u↔v\right)\leq c{\left(u\vee v\right)}^{\gamma -1}{\left(u\wedge v\right)}^{-\gamma }.$$

From Dommers and coworkers [9, Corollary 2.3] we know, for any sequence *π* = (*π*_0_,*π*_1_,…,*π*_*k*_) ∈ [*t*]^*k* + 1^ of distinct vertices,

(4.36) $$P(\pi \subseteq {\text{PA}}_{t})\leq p(\pi_{0},\pi_{1},\text{⋯},\pi_{k}):=\prod_{i=0}^{k-1}\frac{Cm}{{\left(\pi_{i}\wedge \pi_{i+1}\right)}^{\gamma }{\left(\pi_{i}\vee \pi_{i+1}\right)}^{1-\gamma }},$$

where *C* is an absolute constant. The history of (4.36) is that it was first proved by Bollobás and Riordan 4 for *δ* = 0 (so that *γ* = 1 − *γ* = 1/2), and the argument was extended to all *δ* in Dommers and coworkers [9, Corollary 2.3].

Remark 4.10 Proposition 4.9 holds for every random graphs that satisfies (4.36).

We proceed in a similar way as in Section 3.3. Given two vertices *x*,*y* ∈ [*t*], we consider paths *π* = (*π*_0_,*π*_1_,…,*π*_*k*_) between *x* = *π*_0_ and *y* = *π*_*k*_. We fix a decreasing sequence of numbers ${\left(g_{l}\right)}_{l\in N_{0}}$ that serve as truncation values for the *age* of vertices along the path (rather than the degrees as for the configuration model). We say that a path *π* is good when *π*_*l*_ ≥ *g*_*l*_∧*g*_*k* − *l*_ for every *l* = 0,…,*k*, and bad otherwise. In other words, a path is good when the age of vertices does not decrease too much from *π*_0_ to *π*_*k*/2_ and, backwards, from *π*_*k*_ to *π*_*k*/2_. Intuitively, this also means that their degrees do not grow too fast. This means that

(4.37) $$P({\text{dist}}_{{\text{PA}}_{t}}(x,y)\leq 2{\bar{k}}_{t})\leq \sum_{k=1}^{2{\bar{k}}_{t}}P(E_{k}(x,y))+\sum_{k=1}^{{\bar{k}}_{t}}\left[P(F_{k}(x))+P(F_{k}(y))\right],$$

where $E_{k}(x,y)$ is the event of there being a good path of length *k*, as in (3.32), while $F_{k}(x)$ is the event of there being a path *π* with *π*_*i*_ ≥ *g*_*i*_ for *i* ≤ *k* − 1 but *π*_*k*_ < *g*_*k*_, in analogy with (3.33).

Recalling the definition of *p*(*π*_0_,*π*_1_,…,*π*_*k*_) in (4.36), we define for *l* ∈ ℕ,

(4.38) $$f_{l,t}(x,w)={⊮}_{\left\{x\geq g_{0}\right\}}\sum_{\pi_{1}=g_{1}}^{t}\sum_{\pi_{2}=g_{2}}^{t}\text{⋯}\sum_{\pi_{l-1}=g_{l-1}}^{t}p(x,\pi_{1},\text{⋯},\pi_{l-1},w),$$

setting $f_{0,t}(x,w)={⊮}_{\left\{x\geq g_{0}\right\}}$ and $f_{1,t}(x,w)={⊮}_{\left\{x\geq g_{0}\right\}}p(x,w)$. From (4.37) we then obtain

(4.39) $$\begin{array}{cc} P({\text{dist}}_{{\text{PA}}_{t}}(x,y)\leq 2{\bar{k}}_{t})\leq & \sum_{k=1}^{2{\bar{k}}_{t}}\sum_{l=g_{\left\lfloor k/2\right\rfloor }}^{t}f_{\lfloor k/2\rfloor ,t}(x,l)f_{\lceil k/2\rceil ,t}(y,l)\\ & +\sum_{k=1}^{{\bar{k}}_{t}}\sum_{l=1}^{g_{k}-1}f_{k,t}(x,l)+\sum_{k=1}^{{\bar{k}}_{t}}\sum_{l=1}^{g_{k}-1}f_{k,t}(y,l). \end{array}$$

This is the starting point of the proof of Proposition 4.9.

We will show in [6, Appendix A] that the following recursive bound holds

(4.40) $$f_{k,t}(x,l)\leq \alpha_{k}l^{-\gamma }+{⊮}_{\left\{l>g_{k-1}\right\}}\beta_{k}l^{\gamma -1},$$

for suitable sequences (*α*_*k*_)_*k* ∈ ℕ_, (*β*_*k*_)_*k* ∈ ℕ_ and (*g*_*k*_)_*k* ∈ ℕ_ (see [6, Definition A.2]). We will prove recursive bounds on these sequences that guarantee that the sums in (4.39) satisfy the required bounds. We omit further details at this point, and refer the interested reader to [6, Appendix A].

### 4.4 Proof of Statement 2.2

Consider now two independent random vertices $W_{1}^{t}$ and $W_{2}^{t}$ that are uniformly distributed in the set of minimally $k_{t}^{-}$‐connected vertices $M_{k_{t}^{-}}$. We set

(4.41) $$E_{t}:=\left\{\text{dist}(U_{\leq k_{t}^{-}}(W_{1}^{t}),U_{\leq k_{t}^{-}}(W_{2}^{t}))\leq 2{\bar{k}}_{t}\right\}=\left\{\text{dist}(W_{1}^{t},W_{2}^{t})\leq 2k_{t}^{-}+2{\bar{k}}_{t}\right\}$$

and, in analogy with Section 3.2, our goal is to show that

(4.42) $$\lim_{t\to \infty }P(E_{t})=0.$$

We know from Statement 2.1 that, as *t*→*∞*,

(4.43) $$P\left(M_{k_{t}^{-}}\leq \frac{1}{2}E[M_{k_{t}^{-}}]\right)\leq P\left(\left|M_{k_{t}^{-}}-E[M_{k_{t}^{-}}]|>\frac{1}{2}E[M_{k_{t}^{-}}\right]\right)\leq \frac{\mathrm{Var}(M_{k_{t}^{-}})}{\frac{1}{4}E{\left[M_{k_{t}^{-}}\right]}^{2}}=o(1).$$

We also define the event

(4.44) $$B_{t}:=\left\{\max_{v\in [t]}D_{t}(v)\leq \sqrt{t}\right\}$$

and note that it is known (see [13, Theorem 8.13]) that $\lim_{t\to \infty }P(B_{t})=1$. Therefore,

(4.45) $$\begin{array}{ll} P(E_{t}) & =P\left(E_{t}\cap \{M_{k_{t}^{-}}>\frac{1}{2}E[M_{k_{t}^{-}}]\}\cap B_{t}\right)+o(1)\;\\ & =E\left[\sum_{v_{1},v_{2}\in [t]}{⊮}_{\left\{W_{1}^{t}=v_{1},W_{2}^{t}=v_{2}\right\}}{⊮}_{\left\{\text{dist}(v_{1},v_{2})\leq 2k_{t}^{-}+2{\bar{k}}_{t}\right\}}{⊮}_{\left\{M_{k_{t}^{-}}>\frac{1}{2}E[M_{k_{t}^{-}}]\right\}}{⊮}_{B_{t}}\right]+o(1)\;\\ & \leq E\left[\sum_{v_{1},v_{2}\in [t]\setminus [t/2]}\frac{{⊮}_{\left\{v_{1}\in M_{k_{t}^{-}},v_{2}\in M_{k_{t}^{-}}\right\}}}{M_{k_{t}^{-}}^{2}}{⊮}_{\left\{\text{dist}(v_{1},v_{2})\leq 2k_{t}^{-}+2{\bar{k}}_{t}\right\}}{⊮}_{\left\{M_{k_{t}^{-}}>\frac{1}{2}E[M_{k_{t}^{-}}]\right\}}{⊮}_{B_{t}}\right]+o(1)\;\\ & \leq \sum_{v_{1},v_{2}\in [t]\setminus [t/2]}\frac{P\left(v_{1},v_{2}\in M_{k_{t}^{-}},\text{dist}(v_{1},v_{2})\leq 2k_{t}^{-}+2{\bar{k}}_{t},B_{t}\right)}{\frac{1}{4}E{\left[M_{k_{t}^{-}}\right]}^{2}}+o(1).\; \end{array}$$

The contribution of the terms with *v*_1_ = *v*_2_ is negligible, since it gives

$$\frac{\sum_{v_{1}\in [t]\setminus [t/2]}P\left(v_{1}\in M_{k_{t}^{-}}\right)}{\frac{1}{4}E{\left[M_{k_{t}^{-}}\right]}^{2}}=\frac{4}{E[M_{k_{t}^{-}}]}=o(1),$$

because $E[M_{k_{t}^{-}}]\to \infty$ by Proposition 4.6. Henceforth we restrict the sum in (4.45) to *v*_1_ ≠ *v*_2_. Summing over the realizations *H*_1_ and *H*_2_ of the random neighborhoods $U_{\leq k_{t}^{-}}(v_{1})$ and $U_{\leq k_{t}^{-}}(v_{2})$, and over paths *π* from an arbitrary vertex *x* ∈ *∂H*_1_ to an arbitrary vertex *y* ∈ *∂H*_2_, we obtain

(4.46) $$\begin{array}{ll} P(E_{t})\leq \frac{4}{E{\left[M_{k_{t}^{-}}\right]}^{2}} & \sum_{\begin{array}{c} v_{1},v_{2}\in [t]\setminus [t/2]\\ v_{1}\neq v_{2} \end{array}}\;\sum_{H_{1},H_{2}\subseteq [t]\setminus [t/4]}\;\sum_{x\in \partial H_{1},y\in \partial H_{2}}\;\sum_{\begin{array}{c} \pi :x\to y\\ |\pi |\leq 2{\bar{k}}_{t} \end{array}}\;\\ & \;P\left(U_{\leq k_{t}^{-}}(v_{1})=H_{1},U_{\leq k_{t}^{-}}(v_{2})=H_{2},\pi \subseteq {\text{PA}}_{t},B_{t}\right)\;+\;o(1).\; \end{array}$$

The next proposition, proved below, decouples the probability appearing in the last expression:

Proposition 4.11 There is a constant *q* ∈ (1,*∞*) such that, for all *v*_1_,*v*_2_, *H*_1_,*H*_2_ and *π*,

(4.47) $$\begin{array}{ll} & P\left(U_{\leq k_{t}^{-}}(v_{1})=H_{1},U_{\leq k_{t}^{-}}(v_{2})=H_{2},\pi \subseteq {\text{PA}}_{t},B_{t}\right)\;\\ & \leq q\;P\left(U_{\leq k_{t}^{-}}(v_{1})=H_{1},U_{\leq k_{t}^{-}}(v_{2})=H_{2}\right)P\left(\pi \subseteq {\text{PA}}_{t}\right).\; \end{array}$$

The proof of Proposition 4.11 reveals that we can take *q* = 2 for *t* sufficiently large. Using (4.47) in (4.46), we obtain

(4.48) $$\begin{array}{ll} P(E_{t})\leq \frac{4q}{E{\left[M_{k_{t}^{-}}\right]}^{2}}\sum_{v_{1},v_{2}\in [t]\setminus [t/2]}\; & \sum_{H_{1},H_{2}\subseteq [t]\setminus [t/4]}P\left(U_{\leq k}(v_{1})=H_{1}\text{,}\;U_{\leq k}(v_{2})=H_{2}\right)\;\\ & \times \left\{\sum_{x\in \partial H_{1},y\in \partial H_{2}}\;\sum_{\begin{array}{c} \pi :x\to y\\ |\pi |\leq 2{\bar{k}}_{t} \end{array}}P\left(\pi \subseteq {\text{PA}}_{t}\right)\right\}.\; \end{array}$$

If we bound $P\left(\pi \subseteq {\text{PA}}_{t}\right)\leq p(\pi )$ in (4.48), as in (4.36), *the sum over π can be rewritten as the right hand side of* (4.39) (recall (4.37)‐(4.38)). We can thus apply Proposition 4.9 —because the proof of Proposition 4.9 really gives a bound on (4.39)— concluding that the sum over *π* is at most $p/{\left(\log t\right)}^{2}$, where the constant *p* is defined in Proposition 4.9. Since $|\partial H_{1}|=|\partial H_{2}|=m^{k_{t}^{-}}={\left(\log t\right)}^{1-\varepsilon }$ (recall (4.12)), we finally obtain

(4.49) $$P(E_{t})\leq \frac{4q}{E{\left[M_{k_{t}^{-}}\right]}^{2}}\;\frac{p{\left(\log t\right)}^{2(1-\varepsilon )}}{{\left(\log t\right)}^{2}}\;E[M_{k_{t}^{-}}^{2}]=(1+o(1))\frac{4pq}{{\left(\log t\right)}^{2\varepsilon }},$$

where the last step uses Proposition 4.8. This completes the proof that $P(E_{t})=o(1)$. □

Proof of Proposition 4.11 We recall that *H*_1_⊆[*t*]∖[*t*/4] is a labeled directed subgraph containing *v*_1_, such that it is an admissible realization of the neighborhood $U_{\leq k_{t}^{-}}(v_{1})$ of the minimally $k_{t}^{-}$‐connected vertex *v*_1_ (recall Definition 4.2); in particular, *H*_1_∖{*v*_1_}⊆[*t*/2]∖[*t*/4]. We also recall that, for all $u\in H_{1}^{o}:=H_{1}\setminus \partial H_{1}$ and *j* ∈ [*m*], the *j*th edge of *u* is connected to a well specified vertex in *H*_1_, denoted by $\theta_{j}^{H_{1}}(u)$. Analogous considerations apply to *H*_2_.

We have to bound the probability

(4.50) $$P\left(U_{\leq k_{t}^{-}}(v_{1})=H_{1},U_{\leq k_{t}^{-}}(v_{2})=H_{2},\pi \subseteq {\text{PA}}_{t},B_{t}\right),$$

where *π* = (*π*_0_,*π*_1_,…,*π*_*k*_) ∈ [*t*]^*k* + 1^ is a given sequence of vertices with *π*_0_ ∈ *∂H*_1_ and *π*_*k*_ ∈ *∂H*_2_. The event in (4.50) is not factorizable, because the degrees of the vertices in the path *π* are not specified, hence it is not easy to evaluate its probability. To get a factorizable event, we need to give more information. For a vertex *v* ∈ [*t*], define its *incoming neighborhood* N(v) by

(4.51) $$N(v):=\{(u,j)\in [t]\times [m]:\;u\overset{j}{\to }v\}.$$

The key observation is that *the knowledge of 𝒩(v) determines the degree D_s_(v) at any time s ≤ t* (for instance, at time *t* we simply have *D*_*t*_(*v*) = |𝒩(*v*)| + *m*).

We are going to fix the incoming neighborhoods 𝒩(*π*_1_) = *K*_1_, …, 𝒩(*π*_*k* − 1_) = *K*_*k* − 1_ of all vertices in the path *π*, except the extreme ones *π*_0_ and *π*_*k*_ (note that 𝒩(*π*_0_) and 𝒩(*π*_*k*_) reduce to single points in $H_{1}^{o}$ and $H_{2}^{o}$, respectively, because *π*_0_ ∈ *∂H*_1_ and *π*_*k*_ ∈ *∂H*_2_). We emphasize that such incoming neighborhoods allow us to determine whether *π* = (*π*_0_,…,*π*_*k*_) is a path in PA_*t*_. Recalling the definition of the event *B*_*t*_ in (4.44), we restrict to

(4.52) $$|K_{i}|\leq \sqrt{t},\text{for}\;i\in [k-1],$$

and simply drop *B*_*t*_ from (4.50). We will then prove the following relation: for all *v*_1_,*v*_2_, *H*_1_,*H*_2_, *π* = (*π*_0_,…,*π*_*k*_), and for all *K*_1_,…,*K*_*k* − 1_ satisfying (4.52), we have

(4.53) $$\begin{array}{ll} & P\left(U_{\leq k_{t}^{-}}(v_{1})=H_{1},U_{\leq k_{t}^{-}}(v_{2})=H_{2},\{N(\pi_{1})=K_{1},\text{⋯},N(\pi_{k-1})=K_{k-1}\}\right)\;\\ & \leq q\;P\left(U_{\leq k_{t}^{-}}(v_{1})=H_{1},U_{\leq k_{t}^{-}}(v_{2})=H_{2}\right)P\left(N(\pi_{1})=K_{1},\text{⋯},N(\pi_{k-1})=K_{k-1}\right).\; \end{array}$$

Our goal (4.47) follows by summing this relation over all *K*_1_,…,*K*_*k* − 1_ for which *π*⊆PA_*t*_.

The first line of (4.53) is the probability of a factorizable event. In fact, setting for short

$$R\;:=\;(H_{1}^{o}\times [m])\;\cup \;(H_{2}^{o}\times [m])\;\cup \;K_{1}\;\cup \;\text{⋯}\;\cup \;K_{k-1},$$

the event in the first line of (4.53) is the intersection of the following four events (see (4.8)):

$$\begin{array}{c} \underset{u\in H_{1}^{o}}{\cap }\overset{m}{\underset{j=1}{\cap }}\{u\overset{j}{\to }\theta_{j}^{H_{1}}(u)\},\underset{u\in H_{2}^{o}}{\cap }\overset{m}{\underset{j=1}{\cap }}\{u\overset{j}{\to }\theta_{j}^{H_{2}}(u)\},\overset{k-1}{\underset{i=1}{\cap }}\underset{(u,j)\in K_{i}}{\cap }\{u\overset{j}{\to }\pi_{i}\},\\ \underset{(u,j)\in [t]\times [m]\;\setminus \;R}{\cap }\{u\overset{j}{\to /}(H_{1}\cup H_{2}\cup \pi^{o})\}, \end{array}$$

where we set *π* ^*o*^: = *π*∖{*π*_0_,*π*_*k*_} = (*π*_1_,…,*π*_*k* − 1_). Generalizing (4.9)‐(4.10), we can rewrite the first line of (4.53) as follows, recalling (1.10):

(4.54) $$\begin{array}{ll} & P\left(U_{\leq k_{t}^{-}}(v_{1})=H_{1},U_{\leq k_{t}^{-}}(v_{2})=H_{2},\{N(\pi_{1})=K_{1},\text{⋯},N(\pi_{k-1})=K_{k-1}\}\right)\;\\ & \;=\{\prod_{u\in H_{1}^{o}}\prod_{j=1}^{m}\frac{m+\delta }{c_{u,j}}\}\{\prod_{u\in H_{2}^{o}}\prod_{j=1}^{m}\frac{m+\delta }{c_{u,j}}\}\left\{\prod_{i=1}^{k-1}\prod_{(u,j)\in K_{i}}\frac{D_{u,j-1}(\pi_{i})+\delta }{c_{u,j}}\right\}\;\\ & \;\;\;\;\left\{\prod_{(u,j)\in [t]\times [m]\;\setminus \;R}(1-\frac{D_{u,j-1}(H_{1}\cup H_{2}\cup \pi^{o})+|(H_{1}\cup H_{2}\cup \pi^{o})\cap [u-1]|\delta }{c_{u,j}})\right\}.\; \end{array}$$

We stress that *D*_*u*,*j* − 1_(*π*_*i*_) is *non‐random*, because it is determined by *K*_*i*_. Analogous considerations apply to $D_{u,j-1}(H_{1}\cup H_{2}\cup \pi^{o})$. We have thus obtained a factorizable event.

Next we evaluate the second line of (4.53). Looking back at (4.26)‐(4.28), we have

(4.55) $$\begin{array}{ll} & P\left(U_{\leq k_{t}^{-}}(v_{1})=H_{1},U_{\leq k_{t}^{-}}(v_{2})=H_{2}\right)=\{\prod_{u\in H_{1}^{o}}\prod_{j=1}^{m}\frac{m+\delta }{c_{u,j}}\}\{\prod_{u\in H_{2}^{o}}\prod_{j=1}^{m}\frac{m+\delta }{c_{u,j}}\}\;\\ & \;\;\{\prod_{\left(u,j)\in [t]\times [m]\;\setminus \;(H_{1}^{o}\cup H_{2}^{o})\times [m\right]}(1-\frac{D_{u,j-1}(H_{1}\cup H_{2})+|(H_{1}\cup H_{2})\cap [u-1]|\delta }{c_{u,j}})\}.\; \end{array}$$

On the other hand,

(4.56) $$\begin{array}{ll} & P\left(N(\pi_{1})=K_{1},\text{⋯},N(\pi_{k-1})=K_{k-1}\right)=\left\{\prod_{i=1}^{k-1}\prod_{(u,j)\in K_{i}}\frac{D_{u,j-1}(\pi_{i})+\delta }{c_{u,j}}\right\}\;\\ & \;\left\{\prod_{(u,j)\in [t]\times [m]\;\setminus \;K_{1}\cup \text{⋯}\cup K_{k-1}}(1-\frac{D_{u,j-1}(\pi^{o})+|\pi^{o}\cap [u-1]|\delta }{c_{u,j}})\right\}.\; \end{array}$$

Using the bound (1 − (*a* + *b*)) ≤ (1 − *a*)(1 − *b*) in the second line of (4.54), and comparing with (4.55)‐(4.56), we only need to take into account the missing terms in the product in the last lines. This shows that relation (4.53) holds if one sets *q* = *C*_1_ *C*_2_ therein, where

$$\begin{array}{c} C_{1}:={\left\{\prod_{(u,j)\in K_{1}\;\cup \;\text{⋯}\;\cup \;K_{k-1}}(1-\frac{D_{u,j-1}(H_{1}\cup H_{2})+|(H_{1}\cup H_{2})\cap [u-1]|\delta }{c_{u,j}})\right\}}^{-1},\\ C_{2}:=\{\prod_{\left(u,j)\in (H_{1}^{o}\cup H_{2}^{o})\times [m\right]}(1-\frac{D_{u,j-1}(\pi^{o})+|\pi^{o}\cap [u-1]|\delta }{c_{u,j}}){\;\}}^{-1}. \end{array}$$

To complete the proof, it is enough to give uniform upper bounds on *C*_1_ and *C*_2_, that does not depend on *H*_1_, *H*_2_, *π*. We start with *C*_1_. In the product we may assume *u* > *t*/4, because the terms with *u* ≤ *t*/4 are identically one, since *H*_1_,*H*_2_⊆[*t*]∖[*t*/4]. Moreover, for *u* > *t*/4 we have *c*_*u*,*j*_ ≥ *t*(2*m* + *δ*)/4 ≥ *mt*/4 by (1.11) and *δ* > −*m*. Since $D_{u,j-1}(H_{1}\cup H_{2})\leq 2(m+1)i_{k}$, using 1 − *x* ≥ e^−2*x*^ for *x* small and recalling that *δ* < 0, it follows that

(4.57) $$\begin{array}{ll} C_{1}^{-1} & \geq \prod_{(u,j)\in K_{1}\;\cup \;\text{⋯}\;\cup \;K_{k-1}}\left(1-\frac{2(m+1)i_{k}}{\frac{m}{4}t}\right)\;\\ & \geq e^{-\frac{8(m+1)}{tm}|K_{\left[k-1\right]}|i_{k}}, \end{array}$$

where $K_{\left[k-1\right]}=K_{1}\;\cup \;\text{⋯}\;\cup \;K_{k-1}$. Since *i*_*k*_ is given by (4.4), for $k=k_{t}^{-}$ as in (4.12) we have $i_{k}=\frac{m}{m-1}m^{k_{t}^{-}}(1+o(1))\leq 2{\left(\log t\right)}^{1-\varepsilon }$. Recalling also (4.52) and bounding *m* + 1 ≤ 2*m*, we obtain

$$C_{1}\leq e^{\frac{8(m+1)}{tm}|K_{\left[k-1\right]}|i_{k}}\leq e^{16k\;i_{k}/\sqrt{t}}=e^{O(\log t/\sqrt{t})}=1+o(1).$$

For *C*_2_, since $D_{u,j-1}(\pi^{o})\leq D_{t}(\pi^{o})=|K_{\left[k-1\right]}|\leq k\sqrt{t}$, again by (4.52), we get

(4.58) $$C_{2}^{-1}\geq \prod_{\left(u,j)\in (H_{1}^{o}\cup H_{2}^{o})\times [m\right]}\left(1-\frac{k\sqrt{t}}{\frac{m}{4}t}\right)\geq e^{-\frac{8}{m}\;\frac{k}{\sqrt{t}}\;|H_{1}^{o}\cup H_{2}^{o}|m}\geq e^{-16\;k\;i_{k}/\sqrt{t}}=1-o(1).$$

It follows that *C*_1_ *C*_2_ is bounded from above by some constant *q*. This completes the proof.

### 4.5 Proof of Theorem 1.6

Dereich and coworkers 10 have already proved the upper bound. For the lower bound we use Proposition 4.9. In fact, for ${\bar{k}}_{t}$ as in (4.33),

(4.59) $$P\left(H_{t}\leq 2{\bar{k}}_{t}\right)=\sum_{v_{1},v_{2}\in [t]}P\left(V_{1}=v_{1},V_{2}=v_{2},\text{dist}(v_{1},v_{2})\leq 2{\bar{k}}_{t}\right).$$

If *v*_1_ and *v*_2_ are both larger or equal than $g_{0}=\lceil \frac{t}{{\left(\log t\right)}^{2}}\rceil$, then we can apply Proposition 4.9. The probability that *V*_1_ < *g*_0_ or *V*_2_ < *g*_0_ is

(4.60) $$P\left(\left\{V_{1}<g_{0}\}\cup \{V_{2}<g_{0}\right\}\right)\leq 2g_{0}/t=o(1),$$

hence we get

$$\begin{array}{ll} & \frac{1}{t^{2}}\sum_{v_{1},v_{2}\in [t]\setminus [g_{0}]}P\left(\text{dist}(v_{1},v_{2})\leq 2{\bar{k}}_{t}\right)+o(1)\leq \frac{{\left(t-g_{0}\right)}^{2}}{t^{2}}\frac{p}{{\left(\log t\right)}^{2}}+o(1)=o(1), \end{array}$$

and this completes the proof of Theorem 1.6. □

## 5 UPPER BOUND FOR CONFIGURATION MODEL

In this section we prove Statements 2.5 and 2.6 for the configuration model. By the discussion in Section 2.2, this completes the proof of the upper bound in Theorem 1.3, because the proof of Statement 2.4 is already known in the literature, as explained below Statement 2.4.

Throughout this section, the assumptions of Theorem 1.3 apply. In particular, we work on a configuration model CM_*n*_, with *τ* ∈ (2,3) and *d*_min_ ≥ 3.

### 5.1 Proof of Statement 2.5

We first recall what Core_*n*_ is, and define the *k*‐exploration graph.

Recall from (2.8) that, for CM_*n*_, Core_*n*_ is defined as

$${\text{Core}}_{n}=\left\{i\in [n]\;\text{such that}\;d_{i}>{\left(\log n\right)}^{\sigma }\right\},$$

where *σ* > 1/(3 − *τ*). Since the degrees *d*_*i*_ are fixed in the configuration model, Core_*n*_ is a deterministic subset.

For any *v* ∈ [*n*], we recall that *U*_≤*k*_(*v*)⊆[*n*] denotes the subgraph of CM_*n*_ consisting of the vertices at distance at most *k* from *v*. We next consider the *k*‐exploration graph ${\hat{U}}_{\leq k}(v)$ as a modification of *U*_≤*k*_(*v*), where we only explore *d*_min_ half‐edges of the starting vertex *v*, and only *d*_min_ − 1 for the following vertices:

Definition 5.1 (k‐exploration graph in CM_n_) The *k*‐*exploration graph* of a vertex *v* is the subgraph ${\hat{U}}_{\leq k}(v)$ built iteratively as follows:

- Starting from ${\hat{U}}_{\leq 0}(v)=\{v\},$ we consider the first d_min_ half‐edges of v and we pair them, one by one, to a uniformly chosen unpaired half‐edge (see Remark ), to obtain ${\hat{U}}_{\leq 1}(v)$.
- Assume that we have built ${\hat{U}}_{\leq \ell }(v)$, for ℓ ≥ 1, and set ${\hat{U}}_{=\ell }(v):={\hat{U}}_{\leq \ell }(v)\setminus {\hat{U}}_{\leq (\ell -1)}(v)$. For each vertex in ${\hat{U}}_{=\ell }(v),$ we consider the first d_min_ − 1 *unpaired* half‐edges and we pair them, one by one, to a uniformly chosen unpaired half‐edge, to obtain ${\hat{U}}_{\leq (\ell +1)}(v)$. (Note that, by construction, each vertex in ${\hat{U}}_{=\ell }(v)$ has *at least* one already paired half‐edge.)

Definition 5.2 (Collision) In the process of building the k‐exploration graph ${\hat{U}}_{\leq k}(v),$ we say that there is a *collision* when a half‐edge is paired to a vertex already included in the *k*‐exploration graph.

We now prove Statement 2.5. Let us fix *ε* > 0 and set

(5.1) $$k_{n}^{+}=\left(1+\varepsilon \right)\frac{\log\log n}{\log(d_{\min}-1)}.$$

Proposition 5.3 (At most one collision) Under the assumption of Theorem , the following holds with high probability: the $k_{n}^{+}$‐exploration graph of *every* vertex either intersects Core_*n*_, or it has at most one collision.

Let us fix a vertex *v* ∈ [*n*]. We are going to estimate the probability

$$q_{n}(v):=P(\text{there are at least 2 collisions in}\;{\hat{U}}_{\leq k_{n}^{+}}(v)\;\text{and}\;{\hat{U}}_{\leq k_{n}^{+}}(v)\cap {\text{Core}}_{n}=\emptyset ).$$

If we show that $\sup_{v\in [n]}q_{n}(v)=o(1/n)$, then it follows that $\sum_{v\in [n]}q_{n}(v)=o(1)$, completing the proof.

Starting from the vertex *v*, we pair successively one half‐edge after the other, as described in Definition 5.1 (recall also Remark 3.1). In order to build ${\hat{U}}_{\leq k_{n}^{+}}(v)$, we need to make a number of pairings, denoted by ℕ, which is *random*, because collisions may occur. In fact, when there are no collisions, ℕ is deterministic and takes its maximal value given by $i_{k_{n}^{+}}$ in (3.1), therefore

(5.2) $$N\leq i_{k_{n}^{+}}\leq \frac{d_{\min}}{d_{\min}-2}{\left(d_{\min}-1\right)}^{k_{n}^{+}}\leq 3\;{\left(\log n\right)}^{1+\varepsilon }.$$

Introducing the event *C*_*i*_:= “there is a collision when pairing the *i*th half‐edge,” we can write

(5.3) $$q_{n}(v)\leq E\left[\sum_{1\leq i<j\leq N}{⊮}_{\left\{C_{i},C_{j},{\hat{U}}_{\leq k_{n}^{+}}(v)\cap {\text{Core}}_{n}=\emptyset \right\}}\right]$$

$$=\sum_{1\leq i<j\leq 3{\left(\log n\right)}^{1+\varepsilon }}P(C_{i},C_{j},j\leq N,{\hat{U}}_{\leq k_{n}^{+}}(v)\cap {\text{Core}}_{n}=\emptyset ).$$

Let *E*_*ℓ*_ be the event that the first *ℓ* half‐edges are paired to vertices with degree $\leq {\left(\log n\right)}^{\sigma }$ (ie, the graph obtained after pairing the first *ℓ* half‐edges is disjoint from Core_*n*_). Then

(5.4) $$P(C_{i},C_{j},j\leq N,{\hat{U}}_{\leq k_{n}^{+}}(v)\cap {\text{Core}}_{n}=\emptyset )\leq P(C_{i},C_{j},E_{j-1})$$

$$=P(E_{i-1})\;P(C_{i}\;|\;E_{i-1})\;P(C_{j}\;|\;C_{i},E_{j-1}).$$

On the event *E*_*i* − 1_, before pairing the *i*th half‐edge, the graph is composed by at most *i* − 1 vertices, each with degree at most ${\left(\log n\right)}^{\sigma }$, hence, for $i\leq 3{\left(\log n\right)}^{1+\varepsilon }$,

$$P(C_{i}\;|\;E_{i-1})\leq \frac{(i-1){\left(\log n\right)}^{\sigma }}{\ell_{n}-2i+1}\leq \frac{3{\left(\log n\right)}^{1+\varepsilon }{\left(\log n\right)}^{\sigma }}{\ell_{n}-6{\left(\log n\right)}^{1+\varepsilon }}\leq c\;\frac{{\left(\log n\right)}^{\sigma +1+\varepsilon }}{n},$$

for some *c* ∈ (0,*∞*), thanks to *ℓ*_*n*_ = *nμ*(1 + *o*(1)) (recall (3.5)). The same arguments show that

$$P(C_{j}\;|\;C_{i},E_{j-1})\leq c\;\frac{{\left(\log n\right)}^{\sigma +1+\varepsilon }}{n}.$$

Looking back at (5.3)‐(5.4), we obtain

$$\underset{v\in [n]}{\sup}q_{n}(v)\leq \sum_{1\leq i<j\leq 3{\left(\log n\right)}^{1+\varepsilon }}c^{2}\;\frac{{\left(\log n\right)}^{2(\sigma +1+\varepsilon )}}{n^{2}}\leq 9\;c^{2}\;\frac{{\left(\log n\right)}^{2\sigma +4(1+\varepsilon )}}{n^{2}}=o(\frac{1}{n}),$$

which completes the proof.

Corollary 5.4 (Large boundaries) Under the assumptions of Theorem 1.3 and on the event ${\hat{U}}_{\leq k_{n}^{+}}(v)\cap {\text{Core}}_{n}=\emptyset ,$ with high probability, the boundary ${\hat{U}}_{=k_{n}^{+}}(v)$ of the $k_{n}^{+}$‐exploration graph of any vertex *v* ∈ [*n*] contains at least $(d_{\min}-2){\left(d_{\min}-1\right)}^{k_{n}^{+}-1}\geq \frac{1}{2}{\left(\log n\right)}^{1+\varepsilon }$ vertices, each one with at least two unpaired half‐edges.

By Proposition 5.3, with high probability, every $k_{n}^{+}$‐exploration graph has at most one collision before hitting Core_*n*_. The worst case is when the collision happens immediately, that is, a half‐edge incident to *v* is paired to another half‐edge incident to *v*: in this case, removing both half‐edges, the $k_{n}^{+}$‐exploration graph becomes a tree with $(d_{\min}-2){\left(d_{\min}-1\right)}^{k_{n}^{+}-1}$ vertices on its boundary, each of which has at least (*d*_min_ − 1) ≥ 2 yet unpaired half‐edges. Since $(d_{\min}-2)/(d_{\min}-1)\geq \frac{1}{2}$ for *d*_min_ ≥ 3, and moreover ${\left(d_{\min}-1\right)}^{k_{n}^{+}}={\left(\log n\right)}^{1+\varepsilon }$ by (5.1), we obtain the claimed bound.

If the collision happens at a later stage, that is, for a half‐edge incident to a vertex different from the starting vertex *v*, then we just remove the branch from *v* to that vertex, getting a tree with $(d_{\min}-1){\left(d_{\min}-1\right)}^{k_{n}^{+}-1}$ vertices on its boundary. The conclusion follows.

Together, Proposition 5.3 and Corollary 5.4 prove Statement 2.5. □

### 5.2 Proof of Statement 2.6

Consider the $k_{n}^{+}$‐exploration graph $\hat{U}={\hat{U}}_{\leq k_{n}^{+}}(v)$ of a fixed vertex *v* ∈ [*n*], as in Definition 5.1, and let *x*_1_,…,*x*_*N*_ be the (random) vertices on its boundary. We stress that, by Corollary 5.4, with high probability $N\geq \frac{1}{2}{\left(\log n\right)}^{1+\varepsilon }$. Set

(5.7) $$h_{n}=\lceil B\log\log\log n+\;C\rceil ,$$

where *B*,*C* are fixed constants, to be determined later on.

Henceforth we fix a realization *H* of $\hat{U}={\hat{U}}_{\leq k_{n}^{+}}(v)$ and we work *conditionally* on the event $\left\{\hat{U}=H\right\}$. By Remark 3.1, we can complete the construction of the configuration model CM_*n*_ by pairing uniformly all the yet unpaired half‐edges. We do this as follows: for each vertex *x*_1_,…,*x*_*N*_ on the boundary of $\hat{U}$, we explore its neighborhood, looking for *fresh* vertices with higher and higher degree, up to distance *h*_*n*_ (we call a vertex *fresh* if it is connected to the graph for the first time, hence it only has one paired half‐edge). We now describe this procedure in detail:

Definition 5.5 (Exploration procedure) Let x_1_,…,x_N_ denote the vertices on the boundary of a $k_{n}^{+}$‐exploration graph $\hat{U}={\hat{U}}_{\leq k_{n}^{+}}(v)$. We start the exploration procedure from x_1_.

- Step 1. We set $v_{0}^{\left(1\right)}:=x_{1}$ and we pair all its unpaired half‐edges. *Among the fresh vertices* to which $v_{0}^{\left(1\right)}$ has been connected, we call *v*_1_ the one with maximal degree.
- When there are no fresh vertices at some step, the procedure for x_1_ stops.
- Step 2. Assuming we have built $v_{1}^{\left(1\right)},$ we pair all its unpaired half‐edges: among the fresh connected vertices, we denote by $v_{2}^{\left(1\right)}$ the vertex with maximal degree.
- We continue in this way for (at most) h_n_ steps, defining $v_{j}^{\left(1\right)}$ for 0 ≤ j ≤ h_n_ (recall (5.5)).

After finishing the procedure for x_1_, we perform the same procedure for x_2_,x_3_,…,x_N_, defining the vertices $v_{0}^{\left(i\right)},v_{1}^{\left(i\right)},\text{⋯},v_{h_{n}}^{\left(i\right)}$ starting from $v_{0}^{\left(i\right)}=x_{i}$.

Definition 5.6 (Success) Let x_1_,…,x_N_ be the vertices on the boundary of a $k_{n}^{+}$‐exploration graph $\hat{U}={\hat{U}}_{\leq k_{n}^{+}}(v)$. We define the event $S_{x_{i}}:=$ *“x*_*i*_ is a *success”* by

$$S_{x_{i}}:=\{\{v_{0}^{\left(i\right)},v_{1}^{\left(i\right)},\text{⋯},v_{h_{n}}^{\left(i\right)}\}\cap {\text{Core}}_{n}\neq \emptyset \}=\{d_{v_{j}^{\left(i\right)}}>{\left(\log n\right)}^{\sigma }\;\text{for some}\;0\leq j\leq h_{n}\}.$$

Here is the key result, proved below:

Proposition 5.7 (Hitting the core quickly) There exists a constant *η* > 0 such that, for every *n* ∈ N and for every realization *H* of $\hat{U},$

(5.6) $$P(S_{x_{1}}\;|\;\hat{U}=H)\geq \eta ,$$

and, for each *i* = 2,…,*N*,

(5.7) $$P(S_{x_{i}}\;|\;\hat{U}=H,S_{x_{1}}^{c},\text{⋯},S_{x_{i-1}}^{c})\geq \eta .$$

This directly leads to the proof of Statement 2.6, as the following corollary shows:

Corollary 5.8 (Distance between periphery and Core_n_) Under the hypotheses of Theorem , with high probability, the distance of every vertex in the graph from Core_*n*_ is at most

(5.8) $$(1+\varepsilon )\frac{\log\log n}{\log(d_{\min}-1)}+o\left(\log\log n\right).$$

By Corollary 5.4, with high probability, every vertex *v* ∈ [*n*] either is at distance at most $k_{n}^{+}$ from Core_*n*_, or has a $k_{n}^{+}$‐exploration graph $\hat{U}={\hat{U}}_{\leq k_{n}^{+}}(v)$ with at least $N\geq \frac{1}{2}{\left(\log n\right)}^{1+\varepsilon }$ vertices on its boundary. It suffices to consider the latter case. Conditionally on $\hat{U}=H$, the probability that none of these vertices is a success can be bounded by Proposition 5.7:

(5.9) $$P(S_{x_{1}}^{c}\cap \text{⋯}\cap S_{x_{N}}^{c}\;|\;\hat{U}=H)=P(S_{x_{1}}^{c}\;|\;\hat{U}=H)\prod_{j=2}^{N}P(S_{x_{j}}^{c}\;|\;\hat{U}=H,S_{x_{1}}^{c},\text{⋯},S_{x_{j-1}}^{c})$$

$$\leq {\left(1-\eta \right)}^{N}\leq {\left(1-\eta \right)}^{\frac{1}{2}{\left(\log n\right)}^{1+\varepsilon }}=o(1/n).$$

This is uniform over *H*, hence the probability that no vertex is a success, without conditioning, is still *o*(1/*n*). It follows that, with high probability, every *v* ∈ [*n*] has at least one successful vertex on the boundary of its $k_{n}^{+}$‐exploration graph. This means that the distance of every vertex *v* ∈ [*n*] from Core_*n*_ is at most $k_{n}^{+}+h_{n}=k_{n}^{+}+o(\log\log n)$, by (5.7). Recalling (5.1), we have completed the proof of Corollary 5.8 and thus of Statement 2.6.

To prove Proposition 5.7, we need the following technical (but simple) result:

Lemma 5.9 (High‐degree fresh vertices) Consider the process of building a configuration model CM_*n*_ as described in Remark 3.1. Let 𝒢_*l*_ be the random graph obtained after *l* pairings of half‐edges and let *V*_*l*_ be the random vertex incident to the half‐edge to which the *l*th half‐edge is paired. For all *l*,*n* ∈ N and *z* ∈ [0,*∞*) such that

(5.10) $$l\leq \frac{n}{4}(1-F_{d,n}(z)),$$

the following holds:

(5.11) $$P\left(\left.d_{V_{l+1}}>z,V_{l+1}\notin G_{l}\;\right|G_{l}\right)\geq z[1-F_{d,n}(z)]\frac{n}{2\ell_{n}}.$$

In particular, when Conditions 1.1 and 1.2 hold, for every *ζ* > 0 there are *c* > 0, *n*_0_ < *∞* such that

(5.12) $$\forall \;n\geq n_{0},\;0\leq z\leq n^{1/3},\;l\leq n^{1/3}:\;P\left(\left.d_{V_{l+1}}>z,V_{l+1}\notin G_{l}\;\right|G_{l}\right)\geq \frac{c}{z^{\tau -2+\zeta }}.$$

By definition of CM_*n*_, the (*l* + 1)st half‐edge is paired to a uniformly chosen half‐edge among the *ℓ*_*n*_ − 2*l* − 1 that are not yet paired. Consequently

(5.13) $$P\left(\left.d_{V_{l+1}}>z,V_{l+1}\notin G_{l}\;\right|G_{l}\right)=\frac{1}{\ell_{n}-2l-1}\sum_{v\notin G_{l}}d_{v}{⊮}_{\left\{d_{v}>z\right\}}.$$

Since $\left|G_{l}|\leq 2l\leq \frac{n}{2}(1-F_{d,n}(z)\right)$ by (5.10), we obtain

(5.14) $$\frac{1}{\ell_{n}-2l-1}\sum_{v\notin G_{l}}d_{v}{⊮}_{\left\{d_{v}>z\right\}}\geq \frac{z}{\ell_{n}}(n(1-F_{d,n}(z))-|G_{l}|)\geq z(1-F_{d,n}(z))\frac{n}{2\ell_{n}},$$

which proves (5.11).

Assuming Conditions 1.1 and 1.2, we have *ℓ*_*n*_ = *μn*(1 + *o*(1)), with *μ* ∈ (0,*∞*), see (3.5), and there are *c*_1_ > 0 and *α* > 1/2 such that 1 − *F*_***d***,*n*_(*z*) ≥ *c*_1_ *z* ^−(*τ* − 1)^ for 0 ≤ *z* ≤ *n* ^*α*^. Consequently, for 0 ≤ *z* ≤ *n* ^1/3^, the right hand side of (5.10) is at least $\frac{n}{4}\frac{c_{1}}{n^{(\tau -1)/3}}$. Note that (*τ* − 1)/3 < 2/3 (because *τ* < 3), hence we can choose *n*_0_ so that $\frac{n}{4}\frac{c_{1}}{n^{(\tau -1)/3}}\geq n^{1/3}$ for all *n* ≥ *n*_0_. This directly leads to (5.12).

With Lemma 5.9 in hand, we are able to prove Proposition 5.7:

Proof of Proposition 5.7 We fix *v* ∈ [*n*] and a realization *H* of $\hat{U}={\hat{U}}_{\leq k_{n}^{+}}(v)$. We abbreviate

(5.15) $$P^{*}(\;\cdot \;):=P(\;\cdot \;|\;\hat{U}=H).$$

The vertices on the boundary of $\hat{U}$ are denoted by *x*_1_,…,*x*_*N*_. We start proving (5.6), hence we focus on *x*_1_ and we define $v_{0}^{\left(1\right)},v_{1}^{\left(1\right)},\text{⋯},v_{h_{n}}^{\left(1\right)}$ as in Definition 5.5, with $v_{0}^{\left(1\right)}=x_{1}$.

We first fix some parameters. Since 2 < *τ* < 3, we can choose *ζ*,*γ* > 0 small enough so that

(5.16) $$\xi :=1-e^{\gamma }(\tau -2+\zeta )>0.$$

Next we define a sequence ${\left(g_{\ell }\right)}_{\ell \in N_{0}}$ that grows *doubly exponentially* fast:

(5.17) $$g_{\ell }:=2^{e^{\gamma \ell }}=\exp((\log2)\exp(\gamma \;\ell )).$$

Then we fix *B* = 1/*γ* and C=log(σ/log2) in (5.7), where *σ* is the same constant as in Core_*n*_, see (2.8). With these choices, we have

(5.18) $$g_{h_{n}}=e^{\sigma e^{\left\lceil \log\log\log n\right\rceil }}>e^{\sigma \log\log n}={\left(\log n\right)}^{\sigma },\text{while}\;g_{h_{n}-1}<{\left(\log n\right)}^{\sigma }.$$

Roughly speaking, the idea is to show that, with positive probability, one has $d_{v_{j}^{\left(1\right)}}>g_{j}$. As a consequence, $d_{v_{h_{n}}^{\left(1\right)}}>g_{h_{n}}\geq {\left(\log n\right)}^{\sigma }$, that is $v_{h_{n}}^{\left(1\right)}$ belongs to Core_*n*_ and *x*_1_ is a success. The situation is actually more involved, since we can only show that $d_{v_{j}^{\left(1\right)}}>g_{j}$ before reaching Core_*n*_.

Let us make the above intuition precise. Recalling (5.15), let us set

$$H_{-1}:=\emptyset ,H_{0}:=H,H_{k}:=H\cup \{v_{1}^{\left(1\right)},\text{⋯},v_{k}^{\left(1\right)}\}\;\text{for}\;1\leq k\leq h_{n}.$$

Then we introduce the events

(5.19) $$T_{\ell }:=\overset{\ell }{\underset{k=0}{\cup }}\{d_{v_{k}^{\left(1\right)}}>{\left(\log n\right)}^{\sigma }\},W_{\ell }:=\overset{\ell }{\underset{k=0}{\cap }}\{d_{v_{k}^{\left(1\right)}}>g_{k},v_{k}^{\left(1\right)}\notin H_{k-1}\}.$$

In words, the event *T*_*ℓ*_ means that one of the vertices $v_{0}^{\left(1\right)},\text{⋯},v_{\ell }^{\left(1\right)}$ has already reached Core_*n*_, while the event *W*_*ℓ*_ means that the degrees of vertices $v_{0}^{\left(1\right)},\text{⋯},v_{\ell }^{\left(1\right)}$ grow at least like *g*_0_,…,*g*_*ℓ*_ and, furthermore, each *v*_*k*_ is a fresh vertex (this is actually already implied by Definition 5.5, otherwise *v*_*k*_ would not even be defined). We finally set

$$E_{0}:=W_{0},\;E_{j}:=T_{j-1}\cup W_{j}\;\text{for}\;\;1\leq j\leq h_{n}.$$

Note that $T_{h_{n}}$ coincides with $S_{x_{1}}=$ “*x*_1_ is a success.” Also note that $W_{h_{n}}\subseteq \{d_{v_{h_{n}}^{\left(1\right)}}>{\left(\log n\right)}^{\sigma }\}$, because $d_{v_{h_{n}}^{\left(1\right)}}>g_{h_{n}}>{\left(\log n\right)}^{\sigma }$ by (5.18), hence

$$E_{h_{n}}=T_{h_{n}-1}\cup W_{h_{n}}\subseteq T_{h_{n}-1}\cup \{d_{v_{h_{n}}^{\left(1\right)}}>{\left(\log n\right)}^{\sigma }\}=T_{h_{n}}=S_{x_{1}}.$$

Consequently, if we prove that $P^{*}(E_{h_{n}})\geq \eta$, then our goal $P^{*}(S_{x_{1}})\geq \eta$ follows (recall (5.6)).

The reason for working with the events *E*_*j*_ is that their probabilities can be controlled by an induction argument. Recalling (5.15), we can write

(5.20) $$P^{*}(E_{j+1})=P^{*}(T_{j})+P^{*}(T_{j}^{c}\cap W_{j+1})$$

$$=P^{*}(T_{j})+P(d_{v_{j+1}^{\left(1\right)}}>g_{j+1},v_{j+1}^{\left(1\right)}\notin H_{j}\;|\;\{\hat{U}=H\}\cap T_{j}^{c}\cap W_{j})\;P^{*}(T_{j}^{c}\cap W_{j}).$$

The key point is the following estimate on the conditional probability, proved below:

(5.21) $$P(d_{v_{j+1}^{\left(1\right)}}>g_{j+1},v_{j+1}^{\left(1\right)}\notin H_{j}\;|\;\{\hat{U}=H\}\cap T_{j}^{c}\cap W_{j})\;\geq \;1-\varepsilon_{j},\text{where}\;\varepsilon_{j}:=e^{-c{\left(g_{j}\right)}^{\xi }/2},$$

with *ξ* > 0 is defined in (5.16) and *c* > 0 is the constant appearing in relation (5.12). This yields

$$P^{*}(E_{j+1})\;\geq \;P^{*}(T_{j})+(1-\varepsilon_{j})\;P^{*}(T_{j}^{c}\cap W_{j})\;\geq \;(1-\varepsilon_{j})(P^{*}(T_{j})+\;P^{*}(T_{j}^{c}\cap W_{j}))$$

$$\;=\;(1-\varepsilon_{j})P^{*}(T_{j}\cup W_{j})\;\geq \;(1-\varepsilon_{j})P^{*}(T_{j-1}\cup W_{j})$$

$$\;=\;(1-\varepsilon_{j})P^{*}(E_{j}),$$

which leads us to

$$P^{*}(E_{h_{n}})\geq P^{*}(E_{0})\prod_{j=0}^{h_{n}-1}(1-\varepsilon_{j})\geq P^{*}(E_{0})\prod_{j=0}^{\infty }(1-\varepsilon_{j})=:\eta .$$

Since $\sum_{j\geq 0}\varepsilon_{j}<\infty$ and *ε*_*j*_ < 1 for every *j* ≥ 0, by (5.21) and (5.17), the infinite product is strictly positive. Also note that $P^{*}(E_{0})=P^{*}(d_{v_{0}^{\left(1\right)}}\geq 2)=1$, because *g*_0_ = 2 and $d_{v_{0}^{\left(1\right)}}\geq d_{\min}\geq 3$. Then *η* > 0, as required.

It remains to prove (5.21). To lighten notation, we rewrite the left hand side of (5.21) as

(5.22) $$q_{j+1}:=P(d_{v_{j+1}^{\left(1\right)}}>g_{j+1},v_{j+1}^{\left(1\right)}\notin H_{j}\;|\;D_{j}),\text{where}\;D_{j}:=\{\hat{U}=H\}\cap T_{j}^{c}\cap W_{j}.$$

Note that, on the event *D*_*j*_⊆*W*_*j*_, vertex $v_{j}^{\left(1\right)}$ is fresh (ie, it is connected to the graph for the first time), hence it has $m=d_{v_{j}^{\left(1\right)}}-1$ unpaired half‐edges. These are paired uniformly, connecting $v_{j}^{\left(1\right)}$ to (not necessarily distinct) vertices *w* ^(1)^,…,*w* ^(*m*)^. Let us introduce for 1 ≤ *ℓ* ≤ *m* the event

(5.23) $$C_{\ell }:=\overset{\ell }{\underset{k=1}{\cap }}\{d_{w^{\left(k\right)}}>g_{j+1},w^{\left(k\right)}\notin H_{j}{\;\}}^{c}.$$

By Definition 5.5, $v_{j+1}^{\left(1\right)}$ is the *fresh* vertex with maximal degree among them, hence

$$\{d_{v_{j+1}^{\left(1\right)}}>g_{j+1},v_{j+1}^{\left(1\right)}\notin H_{j}{\;\}}^{c}=C_{m}.$$

Since $m=d_{v_{j}^{\left(1\right)}}-1>g_{j}-1$ on *W*_*j*_⊆*D*_*j*_, the left hand side of (5.21) can be estimated by

(5.24) $$\begin{array}{ll} q_{j+1} & =1-P(C_{m}\;|\;D_{j})\geq 1-\prod_{k=1}^{g_{j}-1}P(C_{k}\;|\;D_{j}\cap C_{k-1})\;\\ & =1-\prod_{k=1}^{g_{j}-1}(1-P(d_{w^{\left(k\right)}}>g_{j+1},w^{\left(k\right)}\notin H_{j}\;|\;D_{j}\cap C_{k-1})).\; \end{array}$$

We claim that we can apply relation (5.12) from Lemma 5.9 to each of the probabilities in the last line of (5.24). To justify this claim, we need to look at the conditioning event $D_{j}\cap C_{k-1}$, recalling (5.23), (5.22) and (5.19). In order to produce it, we have to do the following:

- First we build the $k_{n}^{+}$‐exploration graph ${\hat{U}}_{\leq k_{n}^{+}}(v)=H$, which requires to pair at most $O({\left(d_{\min}-1\right)}^{k_{n}^{+}})=O({\left(\log n\right)}^{1+\varepsilon })$ half‐edges (recall Definition 5.1);
- Next, starting from the boundary vertex *x*_1_, we generate the fresh vertices $v_{0}^{\left(1\right)},\text{⋯},v_{j}^{\left(1\right)}$ *all outside* Core_*n*_, because we are on the event $T_{j}^{c}$, and this requires to pair a number of half‐edges which is at most ${\left(\log n\right)}^{\sigma }j\leq {\left(\log n\right)}^{\sigma }h_{n}=O({\left(\log n\right)}^{\sigma +1})$;
- Finally, in order to generate *w* ^(1)^,…,*w* ^(*k* − 1)^, we pair exactly *k* − 1 half‐edges, and note that $k-1\leq g_{j}-1\leq g_{h_{n}}-1=O({\left(\log n\right)}^{\sigma })$ (always because *v*_*j*_∉Core_*n*_).

It follows that the conditioning event $D_{j}\cap C_{k-1}$ is in the *σ*‐algebra generated by 𝒢_*l*_ for $l\leq O({\left(\log n\right)}^{1+\sigma +\varepsilon })$ (we use the notation of Lemma 5.9). In particular, *l* ≤ *n* ^1/3^. Also note that *z* = *g*_*j* + 1_≤ $g_{h_{n}}=O({\left(\log n\right)}^{\sigma })$, see (5.18), hence also *z* ≤ *n* ^1/3^. Applying (5.12), we get

(5.25) $$\begin{array}{ll} q_{j+1} & \geq 1-(1-\frac{c}{{\left(g_{j+1}\right)}^{\tau -2+\zeta }}{\;)}^{g_{j}-1}\geq 1-\exp(-c\frac{g_{j}-1}{{\left(g_{j+1}\right)}^{\tau -2+\zeta }})\;\\ & \geq 1-\exp(-\frac{c}{2}\;\frac{g_{j}}{{\left(g_{j+1}\right)}^{\tau -2+\zeta }})\; \end{array}$$

because 1 − *x* ≤ e^−*x*^ and *n* − 1 ≥ *n*/2 for all *n* ≥ 2 (note that *g*_*j*_ ≥ *g*_0_ = 2). Since $g_{j+1}={\left(g_{j}\right)}^{e^{\gamma }}$, by (5.17), we finally arrive at

(5.26) $$q_{j+1}\geq 1-\exp(-\frac{c}{2}\;{\left(g_{j}\right)}^{1-e^{\gamma }(\tau -2+\zeta )})=1-e^{-c\;{\left(g_{j}\right)}^{\xi }/2},$$

which is precisely (5.21). This completes the proof of (5.6).

In order to prove (5.7), we proceed in the same way: for any fixed 2 ≤ *i* ≤ *N*, we start from the modification of (5.15) given by $P^{*}(\;\cdot \;):=P(\;\cdot \;|\;\hat{U}=H,S_{x_{1}}^{c},\text{⋯},S_{x_{i-1}}^{c})$ and we follow the same proof, working with the vertices $v_{1}^{\left(i\right)}$, …, $v_{h_{n}}^{\left(i\right)}$ instead of $v_{1}^{\left(1\right)},\text{⋯},v_{h_{n}}^{\left(1\right)}$ (recall Definition 5.5). We leave the details to the reader.

## 6 UPPER BOUND FOR PREFERENTIAL ATTACHMENT MODEL

In this section we prove Statements 2.5 and 2.6 for the preferential attachment model. By the discussion in Section 2.2, this completes the proof of the upper bound in Theorem 1.5, because the proof of Statement 2.4 is already known in the literature, as explained below Statement 2.4.

### 6.1 Proof of Statement 2.5

Recall the definition of Core_*t*_ in (2.8). It is crucial that in Core_*t*_, we let *D*_*t*/2_(*v*) be large. We again continue to define what a *k*‐exploration graph and its collisions are, but this time for the preferential attachment model:

Definition 6.1 (k‐exploration graph) Let (PA_t_)_t ≥ 1_ be a preferential attachment model. For v ∈ [t], we call the *k‐exploration graph* of *v* to be the subgraph of PA_*t*_, where we consider the *m* edges originally incident to *v*, and the *m* edges originally incident to any other vertex that is connected to *v* in this procedure, up to distance *k* from *v*.

Definition 6.2 (Collision) Let (PA_t_)_t ≥ 1_ be a preferential attachment model with m ≥ 2, and let v be a vertex. We say that we have a *collision* in the *k*‐exploration graph of *v* when one of the *m* edges of a vertex in the *k*‐exploration graph of *v* is connected to a vertex that is already in the *k*‐exploration graph of *v*.

Now we want to show that every *k*‐exploration graph has at most a finite number of collisions before hitting the Core_*t*_, as we did for the configuration model. The first step is to use Dommers and coworkers [9, Lemma 3.9]:

Lemma 6.3 (Early vertices have large degree) Fix *m* ≥ 1. There exists *a* > 0 such that

(6.1) $$P(\min_{i\leq t^{a}}D_{t}(i)\geq {\left(\log t\right)}^{\sigma })\to 1$$

for some *σ* > 1/(3 − *τ*). As consequence, [*t* ^*a*^]⊆Core_*t*_ with high probability.

In agreement with (2.10) (see also (4.12)), we set

(6.2) $$k_{t}^{+}=(1+\varepsilon )\frac{\log\log t}{\log m}.$$

We want to prove that the exploration graph ${\hat{U}}_{\leq k_{t}^{+}}(v)$ has at most a finite number of collisions before hitting Core_*t*_, similarly to the case of CM_*n*_, now for PA_*t*_. As it is possible to see from (2.8), Core_*t*_⊆[*t*/2], that is, is a subset defined in PA_*t*_ when the graph has size *t*/2. As a consequence, we do not know the degree of vertices in [*t*/2] when the graph has size *t*. However, in Dommers and coworkers [9, Appendix A.4] the authors prove that at time *t* all the vertices *t*/2 + 1,…,*t* have degree smaller than ${\left(\log t\right)}^{\sigma }$.

We continue by giving a bound on the degree of vertices that are not in Core_*t*_. For vertices *i* ∈ [*t*/2]∖Core_*t*_ we know that $D_{t/2}(i)<{\left(\log t\right)}^{\sigma }$, see (2.8), but in principle their degree *D*_*t*_(*i*) at time *t* could be quite high. We need to prove that this happens with very small probability. Precisely, we prove that, for some *B* > 0,

(6.3) $$P\left(\max_{i\in [t/2]\setminus {\text{Core}}_{t}}D_{t}(i)\geq (1+B){\left(\log t\right)}^{\sigma }\right)=o(1).$$

This inequality implies that when a degree is at most ${\left(\log t\right)}^{\sigma }$ at time *t*/2, then it is unlikely to grow by $B{\left(\log t\right)}^{\sigma }$ between time *t*/2 and *t*. This provides a bound on the cardinality of incoming neighborhoods that we can use in the definition of the exploration processes that we will rely on, in order to avoid Core_*t*_. We prove (6.3) in the following lemma that is an adaptation of the proof of Dommers and coworkers [9, Lemma A.4]. Its proof is deferred to [6, Appendix B]:

Lemma 6.4 (Old vertex not in Core_*t*_) There exists *B* ∈ (0,*∞*) such that, for every *i* ∈ [*t*/2],

(6.4) $$P\left(D_{t}(i)\geq (1+B){\left(\log t\right)}^{\sigma }|D_{t/2}(i)<{\left(\log t\right)}^{\sigma }\right)=o(1/t).$$

We can now get to the core of the proof of Statement 2.5, that is we show that there are few collisions before reaching Core_*t*_:

Lemma 6.5 (Few collisions before hitting the core) Let (PA_*t*_)_*t* ≥ 1_ be a preferential attachment model, with *m* ≥ 2 and *δ* ∈ ( − *m*,0). Fix *a* ∈ (0,1) and *l* ∈ ℕ such that *l* > 1/*a*. With $k_{t}^{+}$ as in (), the probability that there exists a vertex *v* ∈ [*t*] such that its $k_{t}^{+}$‐exploration graph has at least *l* collisions before hitting ${\text{Core}}_{t}\cup [t^{a}]$ is *o*(1).

Next we give a lower bound on the number of vertices on the boundary of a $k_{n}^{+}$‐exploration graph. First of all, for any fixed *a* ∈ (0,1), we notice that the probability of existence of a vertex in [*t*]∖[*t* ^*a*^], that has only self loops is *o*(1). Indeed, the probability that a vertex *s* has only self‐loops is $O(\frac{1}{s^{m}})$. Thus, the probability that there exists a vertex in [*t*]∖[*t* ^*a*^] that has only self‐loops is bounded above by

(6.5) $$\sum_{s>t^{a}}O\left(\frac{1}{s^{m}}\right)=O(t^{-a(m-1)})=o(1),$$

since we assume that *m* ≥ 2. We can thus assume that no vertex in [*t*]∖[*t* ^*a*^] has only self‐loops. This leads us formulate the following Lemma, whose proof is also deferred to [6, Appendix B].

Lemma 6.6 (Lower bound on boundary vertices) Let (PA_*t*_)_*t* ≥ 1_ be a preferential attachment model, with *m* ≥ 2 and *δ* ∈ ( − *m*,0). For *a* ∈ (0,1), consider a vertex $v\in [t]\setminus ({\text{Core}}_{t}\cup [t^{a}])$ and its *k*‐exploration graph. If there are at most *l* collisions in the *k*‐exploration graph, and no vertex in [*t*]∖[*t* ^*a*^] has only self loops, then there exists a constant *s* = *s*(*m*,*l*) > 0 such that the number of vertices in the boundary of the *k*‐exploration graph is at least *s*(*m*,*l*)*m* ^*k*^.

Together, Lemmas 6.3, 6.5 and 6.6 complete the proof of Statement 2.5.

The rest of this section is devoted to the proof of Lemma 6.5. We first need to introduce some notation, in order to be able to express the probability of collisions. We do this in the next subsection.

#### 6.1.1 Ulam‐Harris notation for trees

Define

$$W_{\ell }:={\left[m\right]}^{\ell },W_{\leq k}:=\overset{k}{\underset{\ell =0}{\cup }}W_{\ell },$$

where $W_{0}:=\emptyset$. We use *W*_≤*k*_ as a universal set to label any regular tree of depth *k*, where each vertex has *m* children. This is sometimes called the *Ulam‐Harris notation* for trees.

Given *y* ∈ *W*_*ℓ*_ and *z* ∈ *W*_*m*_, we denote by (*y*,*z*) ∈ *W*_*ℓ* + *m*_ the concatenation of *y* and *z*. Given *x*,*y* ∈ *W*_≤*k*_, we write *y*≽*x* if *y* is a descendant of *x*, that is *y* = (*x*,*z*) for some *z* ∈ *W*_≤*k*_.

Given a finite number of points *z*_1_,…,*z*_*m*_ ∈ *W*_≤*k*_, abbreviate ${\vec{z}}_{m}=(z_{1},\text{⋯},z_{m})$, and define $W_{\leq k}^{\left({\vec{z}}_{m}\right)}$ to be the tree obtained from *W*_≤*k*_ by cutting the branches starting from any of the *z*_*i*_'s (including the *z*_*i*_'s themselves):

(6.6) $$W_{\leq k}^{\left({\vec{z}}_{k}\right)}:=\{x\in W_{\leq k}:\;x≽/z_{1},\text{⋯},x≽/z_{m}\}.$$

Remark 6.7 (Total order) The set W_≤k_ comes with a natural total order relation, called *shortlex order*, in which shorter words precede longer ones, and words with equal length are ordered lexicographically. More precisely, given *x* ∈ *W*_*ℓ*_ and *y* ∈ *W*_*m*_, we say that *x* precedes *y* if either *ℓ* < *m*, or if *ℓ* = *m* and *x*_*i*_ ≤ *y*_*i*_ for all 1 ≤ *i* ≤ *ℓ*. We stress that this is a *total* order relation, unlike the descendant relation ≽ which is only a partial order. (Of course, if *y*≽*x*, then *x* precedes *y*, but not vice versa).

#### 6.1.2 Collisions

We recall that, given *z* ∈ [*t*] and *j* ∈ [*m*], the *j*th half‐edge starting from vertex *z* in PA_*t*_ is attached to a random vertex, denoted by *ξ*_*z*,*j*_. We can use the set *W*_≤*k*_ to label the exploration graph ${\hat{U}}_{\leq k}(v)$, as follows:

(6.7) $${\hat{U}}_{\leq k}(v)=\{V_{z}{\;\}}_{z\in W_{\leq k}},$$

where $V_{\emptyset }=v$ and, iteratively, $V_{z}=\xi_{V_{x},j}$ for *z* = (*x*,*j*) with *x* ∈ *W*_≤*k* − 1_ and *j* ∈ [*m*].

The first vertex generating a *collision* is $V_{Z_{1}}$, where the random index *Z*_1_ ∈ *W*_≤*k*_ is given by

$$Z_{1}:=\min\{z\in W_{\leq k}:\;V_{z}=V_{y}\;\text{for some}\;y\;\text{which precedes}\;z\},$$

where “min” refers to the total order relation on *W*_≤*k*_ as defined in Remark 6.7.

Now comes a tedious observation. Since $V_{Z_{1}}=V_{y}$ for some *y* which precedes *Z*_1_, by definition of *Z*_1_, then all descendants of *Z*_1_ will coincide with the corresponding descendants of *y*, that is $V_{\left(Z_{1},r\right)}=V_{\left(y,r\right)}$ for all *r*. In order not to over count collisions, in defining the second collision index *Z*_2_, we avoid exploring the descendants of index *Z*_1_, that is we only look at indices in $W_{\leq k}^{\left(Z_{1}\right)}$, see (6.6). The second vertex representing a (true) collision is then $V_{Z_{2}}$, where we define

$$Z_{2}:=\min\{z\in \overset{\left(Z_{1}\right)}{\underset{\leq k}{W}}:z\;\text{follows}\;Z_{1},\text{ie,}\;\;V_{z}=V_{y}\;\text{for some}\;y\;\text{which precedes}\;z\},$$

Iteratively, we define

$$Z_{i+1}:=\min\{z\in \overset{\left({\vec{Z}}_{i}\right)}{\underset{\leq k}{W}}:z\;\text{follows}\;Z_{i},\text{ie,}\;V_{z}=V_{y}\;\text{for some}\;y\;\text{which precedes}\;z\},$$

so that $V_{Z_{i}}$ is the *i*th vertex that represents a collision. The procedure stops when there are no more collisions. Denoting by *C* the (random) number of collisions, we have a family

$$Z_{1},Z_{2},\text{⋯},Z_{C}$$

of random elements of *W*_≤*k*_, such that ${\left(V_{Z_{i}}\right)}_{1\leq i\leq C}$ are the vertices generating the collisions.

#### 6.1.3 Proof of Lemma 6.5

Recalling (6.7) and (6.6), given arbitrarily *z*_1_,…,*z*_*l*_ ∈ *W*_≤*k*_, we define

(6.8) $${\hat{U}}_{\leq k}^{\left({\vec{z}}_{l}\right)}(v)=\{V_{z}{\;\}}_{z\in W_{\leq k}^{\left({\vec{z}}_{l}\right)}},$$

that is, we consider a subset of the full exploration graph ${\hat{U}}_{\leq k}(v)$, consisting of vertices *V*_*z*_ whose indexes *z* ∈ *W*_≤*k*_ are not descendants of *z*_1_,…,*z*_*l*_. The basic observation is that

(6.9) $${\hat{U}}_{\leq k}(v)={\hat{U}}_{\leq k}^{\left({\vec{z}}_{l}\right)}(v)\;\text{on the event}\;\{C=l,Z_{1}=z_{1},\text{⋯},Z_{l}=z_{l}\}.$$

In words, this means that to recover the full exploration graph ${\hat{U}}_{\leq k}(v)$, it is irrelevant to look at vertices *V*_*z*_ for *z* that is a descendant of a collision index *z*_1_,…,*z*_*l*_.

We will bound the probability that there are *l* collisions before reaching ${\text{Core}}_{t}\cup [t^{a}]$, occurring at specified indices *z*_1_,…,*z*_*l*_ ∈ *W*_≤*k*_, for $k=k_{t}^{+}$ as in (6.2), as follows:

(6.10) $$\begin{array}{ll} P(C=l,Z_{1}=z_{1},\text{⋯},Z_{l}=z_{l},{\hat{U}}_{\leq k}(v)\cap ({\text{Core}}_{t}\cup [t^{a}])=\emptyset )\;\leq \;\alpha {\left(t\right)}^{l}, & \; \end{array}$$

where for the constant *B* given by Lemma 6.4, we define

(6.11) $$\alpha (t)=\frac{4(1+B)}{m}\frac{{\left(\log t\right)}^{\sigma +1+\varepsilon }}{t^{a}}.$$

Summing (6.10) over *z*_1_,…,*z*_*l*_ ∈ *W*_≤*k*_ we get

$$P(C=l,{\hat{U}}_{\leq k}(v)\cap ({\text{Core}}_{t}\cup [t^{a}])=\emptyset )\leq \alpha {\left(t\right)}^{l}\;|W_{\leq k}{|}^{l}\;.$$

Since, for $k=k_{t}^{+}$ as in (6.2), we can bound

(6.12) $$|W_{\leq k}|=\frac{m^{k+1}-1}{m-1}\leq 2\;m^{k}\leq 2\;{\left(\log t\right)}^{1+\varepsilon },$$

the probability of having at least *l* collisions, before reaching ${\text{Core}}_{t}\cup [t^{a}]$, is $O(\alpha {\left(t\right)}^{l}{\left(\log t\right)}^{2l})=o(1/t)$, because *l* > 1/*a* by assumption. This completes the proof of Lemma 6.5. It only remains to show that (6.10) holds true.

#### 6.1.4 Proof of (6.10): case l = 1

We start proving (6.10) for one collision. By (6.9), we can replace ${\hat{U}}_{\leq k}(v)$ by ${\hat{U}}_{\leq k}^{\left(z_{1}\right)}(v)$ in the left hand side of (6.10), that is, we have to prove that

(6.13) $$P(C=1,Z_{1}=z_{1},{\hat{U}}_{\leq k}^{\left(z_{1}\right)}(v)\cap ({\text{Core}}_{t}\cup [t^{a}])=\emptyset )\;\leq \;\alpha (t).$$

Since *v*,*k* and *z*_1_ are fixed, let us abbreviate, and recalling (6.8),

(6.14) $$W:=W_{\leq k}^{\left(z_{1}\right)}(v),\hat{U}:={\hat{U}}_{\leq k}^{\left(z_{1}\right)}(v)=\{V_{z}{\;\}}_{z\in W}.$$

Note that $V_{z_{1}}$ is the only collision precisely when $\hat{U}$ is a tree and $V_{z_{1}}\in \hat{U}$. Then (6.13) becomes

(6.15) $$P(\hat{U}\;\text{is a tree},V_{z_{1}}\in \hat{U},\hat{U}\cap ({\text{Core}}_{t}\cup [t^{a}])=\emptyset )\leq \alpha (t)\;.$$

We will actually prove a stronger statement: for any fixed *deterministic* labeled directed tree *H*⊆[*t*] and for any *y* ∈ *H*,

(6.16) $$P(\hat{U}=H,V_{z_{1}}=y,H\cap ({\text{Core}}_{t}\cup [t^{a}])=\emptyset )\leq \frac{\alpha (t)}{2{\left(\log t\right)}^{1+\varepsilon }}\;P(\hat{U}=H,V_{z_{1}}\notin H).$$

This yields (6.15) by summing over *y* ∈ *H*—note that $|H|\leq |W_{\leq k}|\leq 2{\left(\log t\right)}^{1+\varepsilon }$ by (6.12)—and then summing over all possible realizations of *H*.

It remains to prove (6.16). We again use the notion of a *factorizable event,* as in the proof of the lower bound. Since the events in (6.16) are not factorizable, we will specify the incoming neighborhood (*y*) (recall (4.51)) of all *y* ∈ *H*. More precisely, by labeling the vertices of *H*, see (6.14), as

(6.17) $$H={\left\{v_{s}\right\}}_{s\in W}\;\text{and}\;y=v_{\bar{s}},\text{for some}\;\;\bar{s}\in W,$$

we can consider the events $\left\{N(v_{s})=N_{v_{s}}\right\}$ where $N_{v_{s}}$ are (deterministic) disjoint subsets of [*t*] × [*m*]. We say that the subsets ${\left(N_{v_{s}}\right)}_{s\in W}$ are *compatible* with the tree *H* when $(v_{s^{\prime }},j)\in N_{v_{s}}$ whenever *s* = (*s* ^*′*^,*j*) with *s*,*s* ^*′*^ ∈ , *j* ∈ [*m*]. Then we can write

(6.18) $$\{\hat{U}=H\}=\underset{\text{compatible}\;{\left(N_{v_{s}}\right)}_{s\in W}}{\cup }\{N(v_{s})=N_{v_{s}}\;\text{for every}\;s\in W\}.$$

Since the degree of vertex *v*_*s*_ equals $D_{t}(v_{s})=m+|N_{v_{s}}|$, we can ensure that $H\cap ({\text{Core}}_{t}\cup [t^{a}])=\emptyset$ by restricting the union in (6.18) to those $N_{v_{s}}$ satisfying the constraints

(6.19) $$v_{s}>t^{a}\;\text{and}\;|N_{v_{s}}|\leq (1+B){\left(\log t\right)}^{\sigma }-m,\forall s\in W.$$

Finally, if we write

(6.20) $$z_{1}=(x,j)\;\text{for some}\;x\in W,j\in [m],$$

then, since $V_{z_{1}}=\xi_{V_{x},j}$, the event $\left\{V_{z_{1}}=v_{\bar{s}}\right\}$ amounts to require that

(6.21) $$(v_{x},j)\in N_{v_{\bar{s}}}.$$

Let us summarize where we now stand: When we fix a family of ${\left(N_{v_{s}}\right)}_{s\in W}$ that is compatible and satisfies the constraints (6.19) and (6.21), in order to prove (6.16) it is enough to show that

(6.22) $$\begin{array}{ll} & P(N(v_{s})=N_{v_{s}}\;\text{for every}\;s\in W)\;\\ & \;\leq \frac{\alpha (t)}{2{\left(\log t\right)}^{1+\varepsilon }}\;P(N(v_{s})=N_{v_{s}}\;\text{for every}\;s\in W\setminus \{\bar{s}\},\;N(v_{\bar{s}})=N_{v_{\bar{s}}}\setminus \{(v_{x},j)\}).\; \end{array}$$

Let us set

(6.23) $$N:=\underset{s\in W}{\cup }N_{v_{s}}\subseteq [t]\times [m].$$

The probability on the left‐hand side of (6.22) can be factorized, using conditional expectations and the tower property, as a product of two kinds of terms:

- For every edge (*u*,*r*) ∈ *N*—say $(u,r)\in N_{v_{s}}$, with *s* ∈ 𝒲—we have the term
  (6.24) $$\frac{D_{u,r-1}(v_{s})+\delta }{c_{u,r}}$$
  corresponding to the fact that the edge needs to be connected to *v*_*s*_;
- On the other hand, for every edge (*u*,*r*)∉*N*, we have the term
  (6.25) $$1-\frac{D_{u,r-1}(H)+|H\cap [u-1]|\delta }{c_{u,r}},$$
  corresponding to the fact that the edge may not connect to any vertex in *H*.

(We emphasize that all the degrees *D*_·,·_(·) appearing in (6.24) and (6.25) are *deterministic*, since they are fully determined by the realizations of the incoming neighborhoods ${\left(N_{v_{s}}\right)}_{s\in W}$.)

We can obtain the right‐hand side in (6.22) by replacing some terms in the product.

- Among the edges (*u*,*r*) ∈ *N*, whose contribution is (6.24), we have the one that creates the collision, namely (*v*_*x*_,*j*). If we want this edge to be connected *outside H*, as in the right‐hand side in (6.22), we need to divide the left hand side of (6.22) by
  (6.26) $$\left(\frac{D_{v_{x},j-1}(v_{\bar{s}})+\delta }{c_{v_{x},j}}\right){\left(1-\frac{D_{v_{x},j-1}(H)+|H\cap [v_{x}-1]|\delta }{c_{v_{x},j}}\right)}^{-1}.$$
  We also have to replace some other terms corresponding to edges $(u,r)\in N_{v_{\bar{s}}}$, because the degree of vertex $v_{\bar{s}}$ is decreased by one after connecting (*v*_*x*_,*j*) outside *H*. More precisely, for every edge $(u,r)\in N_{v_{\bar{s}}}$ that is younger than (*v*_*x*_,*j*), that is (*u*,*r*) > (*v*_*x*_,*j*), we can reduce the degree of $v_{\bar{s}}$ by one by dividing the left‐hand side of (6.22) by
  (6.27) $$\prod_{\left(u,r)\in N_{v_{\bar{s}}},(u,r)>(v_{x},j\right)}\frac{D_{u,r-1}(v_{\bar{s}})+\delta }{D_{u,r-1}(v_{\bar{s}})-1+\delta }=\frac{D_{t}(v_{\bar{s}})+\delta }{D_{v_{x},j-1}(v_{\bar{s}})+\delta }.$$
  Finally, the contribution of the edges $(u,r)\in N_{v_{s}}$ for $s\neq \bar{s}$ is unchanged.
- For every edge (*u*,*r*)∉*N*, the probability that such edge is not attached to *H*, after we reconnect the edge (*v*_*x*_,*j*), becomes larger, since the degree of *H* is reduced by one.

It follows that the inequality (6.22) holds with $\alpha (t)/(2{\left(\log t\right)}^{1+\varepsilon })$ replaced by *β*, defined by

(6.28) $$\begin{array}{ll} \beta & =\left(\frac{D_{v_{x},j-1}(v_{\bar{s}})+\delta }{c_{v_{x},j}}\right){\left(1-\frac{D_{v_{x},j-1}(H)+|H\cap [v_{x}-1]|\delta }{c_{v_{x},j}}\right)}^{-1}\frac{D_{t}(v_{\bar{s}})+\delta }{D_{v_{x},j-1}(v_{\bar{s}})+\delta }\;\\ & =\left(\frac{D_{t}(v_{\bar{s}})+\delta }{c_{v_{x},j}}\right){\left(1-\frac{D_{v_{x},j-1}(H)+|H\cap [v_{x}-1]|\delta }{c_{v_{x},j}}\right)}^{-1}\;\\ & \leq \left(\frac{D_{t}(v_{\bar{s}})}{c_{v_{x},j}}\right){\left(1-\frac{D_{v_{x},j-1}(H)}{c_{v_{x},j}}\right)}^{-1}=:\beta^{\prime }, \end{array}$$

because *δ* ≤ 0. We only need to show that $\beta^{\prime }\leq \alpha (t)/(2{\left(\log t\right)}^{1+\varepsilon })$.

Since *c*_*v*,*j*_ ≥ *m*(*v* − 1), the first relation in (6.19) yields

$$c_{v_{x},j}\geq t^{a}.$$

Hence, since $D_{t}(v_{\bar{s}})\leq (1+B){\left(\log t\right)}^{\sigma }$ by the second relation in (6.19), we can bound

$$\left(\frac{D_{t}(v_{\bar{s}})}{c_{v_{x},j}}\right)\leq \frac{(1+B){\left(\log t\right)}^{\sigma }}{mt^{a}}.$$

Likewise, since $D_{t}(H)\leq |H|(1+B){\left(\log t\right)}^{\sigma }$, for $k=k_{t}^{+}$ we get, by (6.12),

$${\left(1-\frac{D_{v_{x},j-1}(H)}{c_{v_{x},j}}\right)}^{-1}\leq {\left(1-\frac{2{\left(\log t\right)}^{1+\varepsilon }(1+B){\left(\log t\right)}^{\sigma }}{t^{a}}\right)}^{-1}\leq 2,$$

where the last inequality holds for *t* large enough. Recalling (6.11),

$$\beta^{\prime }\leq 2\frac{(1+B){\left(\log t\right)}^{\sigma }}{mt^{a}}=\frac{\alpha (t)}{2{\left(\log t\right)}^{1+\varepsilon }}.$$

This completes the proof of (6.22), and hence of (6.10), in the case where *l* = 1. □

#### 6.1.5 Proof of (6.10): general case l ≥ 2

The proof for the general case is very similar to that for *l* = 1, so we only highlight the (minor) changes.

In analogy with (6.13), we can replace ${\hat{U}}_{\leq k}(v)$ by ${\hat{U}}_{\leq k}^{\left({\vec{z}}_{l}\right)}(v)$ in the left‐hand side of (6.10), thanks to (6.9). Then, as in (6.14), we write

(6.29) $$W:=W_{\leq k}^{\left({\vec{z}}_{l}\right)}(v),\hat{U}:={\hat{U}}_{\leq k}^{\left({\vec{z}}_{l}\right)}(v)=\{V_{z}{\;\}}_{z\in W}.$$

The extension of (6.16) becomes that for any fixed *deterministic* labeled directed tree *H*⊆[*t*] and for all *y*_1_,…,*y*_*l*_ ∈ *H*,

(6.30) $$\begin{array}{ll} & P(\hat{U}=H,V_{z_{1}}=y_{1},\text{⋯},V_{z_{l}}=y_{l},H\cap ({\text{Core}}_{t}\cup [t^{a}])=\emptyset )\;\\ & \;\;\leq {\left(\frac{\alpha (t)}{2{\left(\log t\right)}^{1+\varepsilon }}\right)}^{l}\;P(\hat{U}=H,V_{z_{1}}\notin H,V_{z_{2}}\notin H,\text{⋯},V_{z_{l}}\notin H).\; \end{array}$$

As in (6.17), we can write

$$H={\left\{v_{s}\right\}}_{s\in W}\;\text{and}\;y_{1}=v_{{\bar{s}}_{1}},\text{⋯},y_{l}=v_{{\bar{s}}_{l}}\;\text{for some}\;\;{\bar{s}}_{1},\text{⋯},{\bar{s}}_{l}\in W.$$

To obtain a factorizable event, we must specify the incoming neighborhoods $N_{v_{s}}=N_{v_{s}}$ for all *s* ∈ , which must be compatible with *H* and satisfy the constraint (6.19). If we write

$$z_{1}=(x_{1},j_{1}),\text{⋯},z_{l}=(x_{l},j_{l}),\text{for some}\;x_{1},\text{⋯},x_{l}\in W,j_{1},\text{⋯},j_{l}\in [m],$$

then we also impose the constraint that obviously generalizes (6.21), namely

$$(v_{x_{1}},j_{1})\in N_{v_{{\bar{s}}_{1}}},\text{⋯},(v_{x_{l}},j_{l})\in N_{v_{{\bar{s}}_{l}}}.$$

The analogue of (6.22) then becomes

(6.31) $$\begin{array}{ll} & P(N(v_{s})=N_{v_{s}}\;\text{for every}\;s\in W)\;\\ & \;\leq {\left(\frac{\alpha (t)}{2{\left(\log t\right)}^{1+\varepsilon }}\right)}^{l}\;P(N(v_{s})=N_{v_{s}}\;\text{for every}\;s\in W\setminus \{{\bar{s}}_{1},\text{⋯},{\bar{s}}_{l}\},\\ & N(v_{{\bar{s}}_{i}})=N_{v_{{\bar{s}}_{i}}}\setminus \{(v_{x_{i}},j_{i})\}\text{for every}\;i=1,\text{⋯},l).\; \end{array}$$

When we define *N* as in (6.23), the probability in the left‐hand side of (6.31) can be factorized in a product of terms of two different types, which are given precisely by (6.24) and (6.25). In order to obtain the probability in the right‐hand side of (6.31), we have to divide the left‐hand side by a product of factors analogous to (6.26) and (6.27). More precisely, (6.26) becomes

(6.32) $$\prod_{i=1}^{l}\left(\frac{D_{v_{x_{i}},j_{i}-1}(v_{{\bar{s}}_{i}})+\delta }{c_{v_{x_{i}},j_{i}}}\right){\left(1-\frac{D_{v_{x_{i}},j_{i}-1}(H)+|H\cap [v_{x_{i}}-1]|\delta }{c_{v_{x_{i}},j_{i}}}\right)}^{-1},$$

while (6.27) becomes

$$\prod_{i=1}^{l}\frac{D_{t}(v_{{\bar{s}}_{i}})+\delta }{D_{v_{x_{i}},j_{i}-1}(v_{{\bar{s}}_{i}})+\delta }.$$

We define *β* accordingly, namely we take the product for *i* = 1,…,*l* of (6.28) with $x,j,\bar{s}$ replaced respectively by $x_{i},j_{i},{\bar{s}}_{i}$. Then it is easy to show that

$$\beta \leq {\left(\frac{\alpha (t)}{2{\left(\log t\right)}^{1+\varepsilon }}\right)}^{l},$$

arguing as in the case *l* = 1. This completes the proof of (6.31). □

### 6.2 Proof of Statement 2.6

The next step is to prove that the boundaries of the $k_{t}^{+}$‐exploration graphs are at most at distance

(6.33) $$h_{t}=\lceil B\log\log\log t+C\rceil$$

from Core_*t*_, where *B*,*C* are constants to be chosen later on. Similarly to the proof in Section 5.2, we consider a $k_{t}^{+}$‐exploration graph, and we enumerate the vertices on the boundary as *x*_1_,…,*x*_*N*_, where $N\geq s(m,l)m^{k_{t}^{+}}$ from Lemma 6.6 and *l* is chosen as in Lemma 6.5. We next define what it means to have a success:

Definition 6.8 (Success) Consider the vertices x_1_,…,x_N_ on the boundary of a $k_{t}^{+}$‐exploration graph. We say that x_i_ is a *success* when the distance between *x*_*i*_ and Core_*t*_ is at most 2*h*_*t*_.

The next lemma is similar to Lemma 5.7 (but only deals with vertices in [*t*/2]):

Lemma 6.9 (Probability of success) Let (PA_*t*_)_*t* ≥ 1_ be a preferential attachment model, with *m* ≥ 2 and *δ* ∈ ( − *m*,0). Consider *v* ∈ [*t*/2]∖Core_*t*_ and its $k_{t}^{+}$‐exploration graph. Then there exists a constant *η* > 0 such that

(6.34) $$P\left(S_{x_{1}}|{\text{PA}}_{t/2}\right)\geq \eta ,$$

and for all *j* = 2,…,*N*,

(6.35) $$P\left(S_{x_{1}}|{\text{PA}}_{t/2},S_{x_{1}}^{c},\text{⋯},S_{x_{j-1}}^{c}\right)\geq \eta .$$

The aim is to define a sequence of vertices *w*_0_,…,*w*_*h*_ that connects a vertex *x*_*i*_ on the boundary with Core_*t*_. In order to do this, we need some preliminar results. We start with the crucial definition of a *t*‐connector:

Definition 6.10 (t‐connector) Let (PA_t_)_t ≥ 1_ be a preferential attachment model, with m ≥ 2. Consider two subsets A,B⊆[t/2], with A∩B=∅. We say that a vertex j ∈ [t]∖[t/2] is a *t‐connector for A and B* if at least one of the edges incident to *j* is attached to a vertex in *A* and at least one is attached to a vertex in *B*.

The notion of *t*‐connector is useful, because, unlike in the configuration model, in the preferential attachment model typically two high‐degree vertices are not directly connected. From the definition of the preferential attachment model, it is clear that the older vertices have with high probability large degree, and the younger vertices have lower degree. When we add a new vertex, this is typically attached to vertices with large degrees. This means that, with high probability, two vertices with high degree can be connected by a young vertex, which is the *t*‐connector.

A further important reason for the usefulness of *t*‐connectors is that we have effectively *decoupled* the preferential attachment model at time *t*/2 and what happens in between times *t*/2 and *t*. When the sets *A* and *B* are appropriately chosen, then each vertex will be a *t*‐connector with reasonable probability, and the events that distinct vertices are *t*‐connectors are close to being independent. Thus, we can use comparisons to binomial random variables to investigate the existence of *t*‐connectors. In order to make this work, we need to identify the structure of PA_*t*/2_ and show that it has sufficiently many vertices of large degree, and we need to show that *t*‐connectors are likely to exist. We start with the latter.

In more detail, we will use *t*‐connectors to generate the sequence of vertices *w*_1_,…,*w*_*h*_ between the boundary of a $k_{n}^{+}$‐exploration graph and the Core_*t*_, in the sense that we use a *t*‐connector to link the vertex *w*_*i*_ to the vertex *w*_*i* + 1_. (This is why we define a vertex *x*_*i*_ to be a success if its distance from Core_*t*_ is at most 2*h*_*t*_, instead of *h*_*t*_.) We rely on a result implying the existence of *t*‐connectors between sets of high total degree:

Lemma 6.11 (Existence of t‐connectors) Let (PA_*t*_)_*t* ≥ 1_ be a preferential attachment model, with *m* ≥ 2 and *δ* ∈ ( − *m*,0). There exists a constant *μ* > 0 such that, for every *A*⊆[*t*/2], and *i* ∈ [*t*/2]∖*A*,

(6.36) $$P(∄j\in [t]\setminus [t/2]:j\;\text{is a}\;t\text{‐connector for}\;i\;\text{and}\;A|{\text{PA}}_{t/2})\leq \exp\left(-\frac{\mu D_{t/2}(A)\;D_{t/2}(i)}{t}\right),$$

where $D_{t/2}(A)=\sum_{v\in A}D_{t/2}(v)$ is the total degree of *A* at time *t*/2.

The proof of this lemma is present in the proof of Dommers and coworkers [9, Proposition 3.2].

Remark 6.12 *Notice that this bound depends on the fact that the number of possible t‐connectors is of order t*.

A last preliminary result that we need is a technical one, which plays the role of Lemma 5.9 for the configuration model and shows that at time *t*/2 there are sufficiently many vertices of high degree, uniformly over a wide range of what ‘large’ could mean:

Lemma 6.13 (Tail of degree distribution) Let (PA_*t*_)_*t* ≥ 1_ be a preferential attachment model, with *m* ≥ 2 and *δ* ∈ ( − *m*,0). Then, for all *ζ* > 0 there exists a constant *c* = *c*(*ζ*) such that, for all $1\leq x\leq {\left(\log t\right)}^{q},$ for any *q* > 0, and uniformly in *t*,

(6.37) $$P_{\geq x}(t)=\frac{1}{t}\sum_{v\in [t]}{⊮}_{\left\{D_{t}(v)\geq x\right\}}\geq cx^{-(\tau -1+\zeta )}.$$

The degree distribution sequence (*p*_*k*_)_*k* ∈ ℕ_ in (1.12) satisfies a power law with exponent *τ* ∈ (2,3). As a consequence, for all *ζ* > 0 there exists a constant $\bar{c}=\bar{c}(\zeta )$ such that

(6.38) $$p_{\geq x}:=\sum_{k\geq x}p_{k}\geq \bar{c}x^{-(\tau -1+\zeta )}.$$

We now use a concentration result on the empirical degree distribution (for details, see [13, Theorem 8.2]), which assures us that there exists a second constant *C* > 0 such that, with high probability, for every *x* ∈ ℕ,

(6.39) $$\left|P_{\geq x}-p_{\geq x}\right|\leq C\sqrt{\frac{\log t}{t}}.$$

Fix now *ζ* > 0, then from this last bound we can immediately write, for a suitable constant $\bar{c}$ as in (6.38),

(6.40) $$P_{\geq x}\geq p_{\geq x}-C\sqrt{\frac{\log t}{t}}\geq \bar{c}x^{-(\tau -1+\zeta )}-C\sqrt{\frac{\log t}{t}}\geq \frac{\bar{c}}{2}x^{-(\tau -1+\zeta )},$$

if and only if

(6.41) $$C\sqrt{\frac{\log t}{t}}=o\left(x^{-(\tau -1+\zeta )}\right).$$

This is clearly true for $x\leq {\left(\log t\right)}^{q}$, for any positive *q*. Taking $c=\bar{c}/2$ completes the proof.

With the above tools, we are now ready to complete the proof of Lemma 6.9:

Proof of Lemma 6.9 As in the proof of Proposition 5.7, we define the super‐exponentially growing sequence *g*_*ℓ*_ as in (5.17), where *γ* > 0 is chosen small enough, as well as *ζ* > 0, so that (5.16) holds. The constants *B* and *C* in the definition (6.33) of *h*_*t*_ are fixed as prescribed below (5.17).

We will define a sequence of vertices *w*_0_,…,*w*_*h*_ such that, for *i* = 1,…,*h*, *D*_*t*_(*w*_*i*_)(*t*) ≥ *g*_*i*_ and *w*_*i* − 1_ is connected to *w*_*i*_. For this, we define, for *i* = 1,…,*h* − 1,

(6.42) $$H_{i}=\{u\in [t]:D_{t/2}(u)\geq g_{i}\}\subseteq [t/2],$$

so that we aim for *w*_*i*_ ∈ *H*_*i*_.

We define the vertices recursively, and start with *w*_0_ = *x*_1_. Then, we consider *t*‐connectors between *w*_0_ and *H*_1_, and denote by *w*_1_ the vertex in *H*_1_ with minimal degree among the ones that are connected to *w*_0_ by a *t*‐connector. Recursively, consider *t*‐connectors between *w*_*i*_ and *H*_*i* + 1_, and denote by *w*_*i* + 1_ the vertex in *H*_*i* + 1_ with minimal degree among the ones that are connected to *w*_*i*_ by a *t*‐connector. Recall (5.18) to see that $g_{h_{t}}\geq {\left(\log t\right)}^{\sigma }$, where *h*_*t*_ is defined in (6.33). The distance between *w*_0_ and Core_*t*_ is at most $2h_{t}=2\lceil B\log\log\log t+C\rceil$. If we denote the event that there exists a *t* connector between *w*_*i* − 1_ and *H*_*i*_ by {*w*_*i* − 1_∼*H*_*i*_}, then we will bound from below

(6.43) $$P(S_{x_{1}}|{\text{PA}}_{t/2})\geq E[\prod_{i=1}^{h_{t}}{⊮}_{\left\{w_{i-1}∼H_{i}\right\}}|{\text{PA}}_{t/2}].$$

In Lemma 6.11, the bound on the probability that a vertex *j* ∈ [*t*]∖[*t*/2] is a *t*‐connector between two subsets of [*t*] is independent of the fact that the other vertices are *t*‐connectors or not. This means that, with $F_{i}$ the *σ*‐field generated by the path formed by *w*_0_,…,*w*_*i*_ and their respective *t*‐connectors,

(6.44) $$E[{⊮}_{\left\{w_{i-1}∼H_{i}\right\}}|{\text{PA}}_{t/2},F_{i-1}]\geq 1-e^{-\mu D_{t/2}(w_{i-1})D_{t/2}(H_{i})/t},$$

where $D_{t}(H_{i})=\sum_{u\in H_{i}}D_{t/2}(u)$. This means that

(6.45) $$E[\prod_{i=1}^{h_{t}}{⊮}_{\left\{w_{i-1}∼H_{i}\right\}}|{\text{PA}}_{t/2}]\geq \prod_{i=1}^{h_{t}}(1-e^{-\mu D_{t/2}(w_{i-1})D_{t/2}(H_{i})/t}).$$

We have to bound every term in the product. Using Lemma 6.13, for *i* = 1,

(6.46) $$1-e^{-\mu D_{t/2}(w_{0})D_{t/2}(H_{1})/t}\geq 1-e^{-\mu D_{t/2}(w_{0})g_{1}P_{\geq g_{1}}(t/2)},$$

while, for *i* = 2,…,*h* − 1

(6.47) $$1-e^{-\mu D_{t/2}(w_{i-1})D_{t/2}(H_{i})/t}\geq 1-e^{-\mu g_{i-1}g_{i}P_{\geq g_{i}}(t/2)}.$$

Applying (6.37) and recalling (5.25)–(5.26), the result is

(6.48) $$\begin{array}{cc} P(S_{x_{1}}|{\text{PA}}_{t}) & \geq (1-e^{-\mu D_{t/2}(w_{0})g_{1}P_{\geq g_{1}}(t/2)})\prod_{i=2}^{h_{t}}(1-e^{-\mu g_{i-1}g_{i}P_{\geq g_{i}}(t/2)})\\ & \geq (1-e^{-\mu mg_{1}P_{\geq g_{1}}(t/2)})\prod_{i=2}^{\infty }(1-e^{-\tilde{c}\;{\left(g_{i}\right)}^{\xi }}), \end{array}$$

for some constant $\tilde{c}$. Since $h_{t}=\lceil B\log\log\log t+C\rceil$, and

(6.49) $$P_{\geq g_{1}}(t/2)\to \sum_{k\geq g_{1}}p_{k}>0$$

with high probability as *t*→*∞*, we can find a constant *η* such that

(6.50) $$(1-e^{-\eta mg_{1}P_{\geq g_{1}}(t/2)})\prod_{i=2}^{h_{t}}(1-e^{-\tilde{c}\;{\left(g_{i}\right)}^{\xi }})>\eta >0,$$

which proves (6.34).

To prove (6.35), we observe that all the lower bounds that we have used on the probability of existence of *t*‐connectors only depend on the existence of sufficiently many potential *t*‐connectors. Thus, it suffices to prove that, on the event $S_{x_{1}}^{c}\cap \text{⋯}\cap S_{x_{j-1}}^{c}$, we have not used too many vertices as *t*‐connectors. On this event, we have used at most *h*_*t*_·(*j* − 1) vertices as *t*‐connectors, which is *o*(*t*). Thus, this means that, when we bound the probability of $S_{x_{j}}$, we still have *t* − *h*_*t*_·(*j* − 1) possible *t*‐connectors, where *j* is at most ${\left(\log t\right)}^{1+\varepsilon }$. Thus, with the same notation as before,

(6.51) $$E[{⊮}_{\left\{w_{i-1}∼H_{i}\right\}}|{\text{PA}}_{t/2},S_{x_{1}}^{c},\text{⋯},S_{x_{j-1}}^{c}]\geq 1-e^{-\mu D_{t/2}(w_{i-1})D_{t/2}(H_{i})/t},$$

so that we can proceed as we did for $S_{x_{1}}$. We omit further details.

We are now ready to identify the distance between the vertices outside the core and the core:

Proposition 6.14 (Distance between periphery and Coret) Let (PA_*t*_)_*t* ≥ 1_ be a preferential attachment model with *m* ≥ 2 and *δ* ∈ ( − *m*,0). Then, with high probability and for all *v* ∈ [*t*]∖Core_*t*_,

(6.52) $${\text{dist}}_{{\text{PA}}_{t}}(v,{\text{Core}}_{t})\leq k_{t}^{+}+2h_{t}.$$

We start by analyzing *v* ∈ [*t*/2]. By Lemma 6.3, with high probability there exists *a* ∈ (0,1] such that [*t* ^*a*^]⊆Core_*t*_. Consider *l* > 1/*a*, and fix a vertex *v* ∈ [*t*/2]. Then, by Lemma 6.5 and with high probability, the $k_{t}^{+}$‐exploration graph starting from *v* has at most *l* collisions before hitting Core_*t*_. By Lemma 6.6 and with high probability, the number of vertices on the boundary of the $k_{t}^{+}$‐exploration graph is at least $N=s(m,l){\left(\log t\right)}^{1+\varepsilon }$. It remains to bound the probability that none of the *N* vertices on the boundary is a success, meaning that it does not reach Core_*t*_ in at most $2h_{t}=2\lceil B\log\log t+C\rceil$ steps.

By Lemma 6.9,

(6.53) $$P(S_{x_{1}}^{c}\cap \text{⋯}\cap S_{x_{N}}^{c}|{\text{PA}}_{t/2})\leq {\left(1-\eta \right)}^{N}=o(1/t),$$

thanks to the bound $N\geq s(m,l){\left(\log t\right)}^{1+\epsilon }$. This means that the probability that there exists a vertex *v* ∈ [*t*/2] such that its $k_{n}^{+}$‐exploration graph is at distance more than Alogloglogt from Core_*t*_ is *o*(1). This proves the statement for all *v* ∈ [*t*/2].

Next, consider a vertex *v* ∈ [*t*]∖[*t*/2]. Lemma 6.5 implies that the probability that there exists a vertex *v* ∈ [*t*]∖[*t*/2] such that its $k_{t}^{+}$‐exploration graph contains more than one collision before hitting ${\text{Core}}_{t}\cup [t/2]$ is *o*(1). As before, the number of vertices on the boundary of a $k_{t}^{+}$‐exploration graph starting at *v* ∈ [*t*]∖[*t*/2] is at least $N\geq s(m,1)m^{k_{n}^{+}}=s(m,1){\left(\log t\right)}^{1+\varepsilon }$. We denote these vertices by *x*_1_,…,*x*_*N*_. We aim to show that, with high probability,

(6.54) $$\Delta_{N}=\sum_{i=1}^{N}{⊮}_{\left(x_{i}\in [t/2]\right)}\geq N/4.$$

For every *i* = 1,…,*N*, there exists a unique vertex *y*_*i*_ such that *y*_*i*_ is in the $k_{t}^{+}$‐exploration graph and it is attached to *x*_*i*_. Obviously, if *y*_*i*_ ∈ [*t*/2] then also *x*_*i*_ ∈ [*t*/2], since *x*_*i*_ has to be older than *y*_*i*_. If *y*_*i*_∉[*t*/2], then

(6.55) $$P\left(x_{i}\in [t/2]|{\text{PA}}_{y_{i}-1}\right)=P\left(y_{i}\to [t/2]|{\text{PA}}_{y_{i}-1}\right)\geq \frac{1}{2},$$

and this bound does not depend on the attaching of the edges of the other vertices {*y*_*j*_:*j* ≠ *i*}.

This means that we obtain the stochastic domination

(6.56) $$\Delta_{N}\geq \sum_{i=1}^{N}{⊮}_{\left(x_{i}\in [t/2]\right)}≽\text{Bin}\left(N,\frac{1}{2}\right),$$

where we write that *X*≽*Y* when the random variable *X* is stochastically larger than *Y*. By concentration properties of the binomial, $\text{Bin}(N,\frac{1}{2})\geq N/4$ with probability at least

(6.57) $$1-e^{-N/4}=1-e^{-s(m,1){\left(\log t\right)}^{1+\varepsilon }/4}=1-o(1/t).$$

Thus, the probability that none of the vertices on the boundary intersected with [*t*/2] is a success is bounded by

(6.58) $$P(S_{x_{1}}^{c}\cap \text{⋯}\cap S_{x_{\Delta_{N}}}^{c}|{\text{PA}}_{t/2})\leq {\left(1-\eta \right)}^{N/4}+o(1/t)=o(1/t).$$

We conclude that the probability that there exists a vertex in [*t*]∖[*t*/2] such that it is at distance more than $k_{t}^{+}+2h_{t}$ from Core_*t*_ is *o*(1).

This completes the proof of Statement 2.6, and thus of Theorem 1.5.

## ACKNOWLEDGEMENTS

We thank the referees for their detailed comments, which improved the paper considerably. The work of FC was partially supported by the ERC Advanced Grant 267356 VARIS and by the PRIN 20155PAWZB “Large Scale Random Structures.” The work of AG was partially supported by University of Milano Bicocca through an EXTRA scholarship that sponsored his visit to Eindhoven University of Technology to complete his master project in March‐May 2014. The work of AG and RvdH is supported by the Netherlands Organisation for Scientific Research (NWO) through the Gravitation NETWORKS Grant 024.002.003. The work of RvdH is also supported by NWO through VICI grant 639.033.806.
